# Health behaviour modelling for prenatal diagnosis in Australia: a geodemographic framework for health service utilisation and policy development

**DOI:** 10.1186/1472-6963-6-109

**Published:** 2006-09-01

**Authors:** Evelyne E Muggli, David McCloskey, Jane L Halliday

**Affiliations:** 1 Public Health Genetics, Murdoch Childrens Research Institute, Royal Children's Hospital, Parkville, Victoria, 3052, Australia; 2 Pathfinder Solutions (Australia) P/L, 3/461 Bourke St, Melbourne, Victoria, 3000, Australia; 3 Department of Paediatrics, University of Melbourne, Victoria, 3010, Australia; 4 Birth Defects Register, Perinatal Data Collection Unit, Public Health Group, Victorian Government Department of Human Services, 15/50 Lonsdale St, Melbourne, Victoria, 3000, Australia

## Abstract

**Background:**

Despite the wide availability of prenatal screening and diagnosis, a number of studies have reported no decrease in the rate of babies born with Down syndrome. The objective of this study was to investigate the geodemographic characteristics of women who have prenatal diagnosis in Victoria, Australia, by applying a novel consumer behaviour modelling technique in the analysis of health data.

**Methods:**

A descriptive analysis of data on all prenatal diagnostic tests, births (1998 and 2002) and births of babies with Down syndrome (1998 to 2002) was undertaken using a Geographic Information System and socioeconomic lifestyle segmentation classifications.

**Results:**

Most metropolitan women in Victoria have average or above State average levels of uptake of prenatal diagnosis. Inner city women residing in high socioeconomic lifestyle segments who have high rates of prenatal diagnosis spend 20% more on specialist physician's fees when compared to those whose rates are average. Rates of prenatal diagnosis are generally low amongst women in rural Victoria, with the lowest rates observed in farming districts. Reasons for this are likely to be a combination of lack of access to services (remoteness) and individual opportunity (lack of transportation, low levels of support and income). However, there are additional reasons for low uptake rates in farming areas that could not be explained by the behaviour modelling. These may relate to women's attitudes and choices.

**Conclusion:**

A lack of statewide geodemographic consistency in uptake of prenatal diagnosis implies that there is a need to target health professionals and pregnant women in specific areas to ensure there is increased equity of access to services and that all pregnant women can make informed choices that are best for them. Equally as important is appropriate health service provision for families of children with Down syndrome. Our findings show that these potential interventions are particularly relevant in rural areas.

Classifying data to lifestyle segments allowed for practical comparisons of the geodemographic characteristics of women having prenatal diagnosis in Australia at a population level. This methodology may in future be a feasible and cost-effective tool for service planners and policy developers.

## Background

Despite the wide availability of prenatal diagnosis and recent advances in antenatal screening to identify pregnancies at high risk for Down syndrome (DS), the extent to which prenatal screening and diagnostic techniques have resulted in changes in live birth prevalence of DS has been variable. A number studies have reported an unchanged live birth prevalence of DS or suggest that a substantial proportion of cases with DS continues to result in a birth, [1-5] while others have found a decrease in the live birth prevalence of DS [6] or that there is variation across countries [7]. Maternal socioeconomic and demographic factors will be contributing to these findings through their role in influencing the utilisation of prenatal diagnosis [8-12].

Victoria, Australia has experienced a relatively stable birth rate over the last 20 years with around 62 000 births annually. Approximately 75% occur in metropolitan Melbourne and one quarter in rural regions. Second trimester maternal serum screening and routine ultrasound are available to all pregnant women. Pregnant women aged 37 years or over, and younger women with an increased risk screening test for chromosomal abnormalities are eligible for prenatal diagnosis free of charge. First trimester combined maternal serum and nuchal translucency screening was introduced in Victoria in 2001 and is currently available to women through private providers with minimal government subsidy. Pregnant women under the age of 37 years who wish to have prenatal diagnosis without a recognised indication can do so through a private provider at their own expense. While second trimester maternal serum screening is available throughout Victoria, all pregnant women residing in rural regions have few options but to travel to a metropolitan centre to have a prenatal diagnostic test.

However, access is only one issue playing a role in the uptake of prenatal diagnosis or the live birth prevalence of DS. The aim of the present study is to use a combination of actual and modelled data to investigate a range of sociodemographic characteristics of women who have prenatal diagnosis in Victoria and to assess a potential relationship between uptake of diagnostic testing and DS birth prevalence, using novel a consumer behaviour modelling technique in the analysis. We incorporate a comparison of an expected rate of DS at term, based on the actual maternal age distribution, with the observed rate of babies born with DS.

Data are investigated by means of a Geographic Information System (GIS) using lifestyle segmentation classifications. Lifestyle segmentation classifications are an example of geocoded data that can identify specific combinations of demographic characteristics linked to locations. Typically, data have been applied in this way by commercial organisations as a method to build models that predict the likely behaviour of customers but more recently these mapping techniques have been adapted for use in disease surveillance and public health management and practice [13-15].

## Methods

### Datasets

#### Births/confinements

The Perinatal Data Collection Unit, Public Health, Victorian Government Department of Human Services (PDCU) has mandatory reporting of every birth (including pregnancy terminations) at or after 20 weeks gestation and data are collected from all maternity hospitals (and homebirths) in Victoria on the Perinatal Statistics Form. Double-entry and other ongoing validation activities ensure data of the highest quality and reliability [16].

#### Birth Defects Register

The Victorian Birth Defects Register has multiple sources of notification: It collects information on all births from the PDCU where there has been a reported birth defect and includes data on pregnancy terminations for birth defects before and after 20 weeks. Additional data are provided by the cytogenetic laboratories and hospital inpatient lists. The completeness of data on chromosome abnormalities has been validated in a number of studies and is considered excellent [17,18].

#### Prenatal diagnosis (PNDx)

There is a long-standing arrangement between the four cytogenetics laboratories in the State and Public Health Genetics at the Murdoch Childrens Research Institute and the PDCU to collect data on every amniocentesis and chorionic villus sampling (CVS) in Victoria. Annual reports collating all data are routinely produced.

### Study population

All women residing in Victoria who had amniocentesis or CVS in 1998 or 2002 and whose expected date of delivery was also in that year were included in the study. This was done to ensure that the births and prenatal tests occurred within the study period and to facilitate record linkage. The years were chosen because second trimester maternal serum screening was made available to all pregnant women in the public health sector in Victoria in 1996/1997 and became well established in 1998. The 2002 data were the most current available and incorporate diagnostic tests prompted by combined first trimester screening for women in the private health care sector, introduced in Victoria in 2001. Denominator data were obtained from all births (to calculate a Down syndrome live birth ratio) or confinements (to estimate uptake of prenatal diagnosis) recorded at the PDCU in 1998 and 2002. Data on births of babies with Down syndrome were obtained from the Birth Defects Register for the years of 1997 to 2002 and included all babies born 20 weeks gestation and later (live births, still births and neonatal deaths), excluding all terminations of pregnancies. Ethics approval for this study was obtained from the Victorian Government Department of Human Services Human Research Ethics Committee.

### Geodemographic segments

The geographic identifier used in this study was the mother's postcode of residence. The allocation of postcodes in Victoria underwent a major change in 1996 followed by the creation of new Local Government Areas (LGAs) which have remained stable since. Where a number of postcodes are divided between two or more different LGAs, allocation to a specific LGA was assigned proportionally by the PDCU on the basis of Australian Bureau of Statistics census data. There are currently 79 LGAs in Victoria, each is made up of a number of Census Collection Districts. The Census Collection District (CCD) is the smallest geographic area as defined by the Australian Standard Geographical Classification and correct allocation to a CCD is only achieved by using a street address. As this variable is not routinely collected by the PDCU, it was necessary to interpolate the LGA data and re-distribute this data to the component CCDs in each LGA. The modelling for this involved first taking the rate of testing that applied in each LGA and applying this standard rate to each component CCD. The modelled information was then aggregated into 58 geodemographic lifestyle segments across the State. These segments are derived from comprehensive household data tabulated by the Australian Census of Population and Housing in 2001 and extensive consumer surveys and were used under the licence of Pathfinder Solutions (Australia) Pty Ltd (Pathfinder Segments of Australia©). Basic segment descriptions used to showcase customer behaviour profiles for commercial purposes, are available as online content (see Additional file 1).

### Data analysis

Women in the prenatal diagnosis and birth cohorts were assigned to two groups according to their age at expected date of delivery: (1) 37 years and over in keeping with Victorian public sector policy on entitlement to publicly funded prenatal diagnosis, and (2) 36 years and younger. Four geodemographic segments, which are predominantly non-residential and 11 segments containing less than 1% of all births in the region (less than 500 births per metropolitan segment and less than 100 births per rural segment) were subsequently excluded. This resulted in 27 metropolitan and 16 rural geodemographic segments. These were ranked by average household income (where 1 was defined as the lowest weekly average household income) to display the data in a simplified socioeconomic (SES) gradient (Table 1).

**Table 1:** Geodemographic segment by increasing SES (average household income)

**Legend to Figues 1–3^§^**	**Graded household income^†^**	**Segment **(© Pathfinder Solutions (Australia) P/L)	**Births **(% of region)
**Metropolitan Victoria**			
1	1.00	Single Parent Public Housing	5.2
2	1.03	Unskilled Battlers	2.5
3	1.20	High Rise Rentals	2.1
4	1.21	Repeat Movers	2.5
5	1.23	Young Single Parent Families	1.2
6	1.24	Living Alone	2.8
7	1.26	Vietnamese Migrant Enclaves	4.6
8	1.32	Ageing Suburban Areas	2.5
9	1.36	Multicultural Mix	2.2
10	1.37	Established Italian Migrants	5.3
11	1.41	Moving Down	6.4
12	1.45	Dual Occupancy Hopefuls	2.3
13	1.49	Established Greek Communities	2.9
14	1.51	Maturing Housing Estates	4.5
15	1.51	Struggling City Fringe	4.9
16	1.51	Settled Trades and Manufacturing	5.7
17	1.55	New Housing Estates	11.8
18	1.58	Prosperous Trades	1.9
19	1.60	Fringe Lifestyle	2.9
20	1.62	Older Money and Asset Rich	3.0
21	1.65	Mature Families	1.1
22	1.67	Inner Suburban Lifestyle Seekers	4.8
23	1.70	Professionals With Young Families	2.5
24	1.71	Rising Wealth	1.5
25	1.71	Moving Up	2.1
26	1.74	Comfortable and Owned Outright	2.4
27	2.00	Asset Rich, Income Rich	2.9
**Rural Victoria**			
a	1.00	Low Income and Ageing Coastal	4.9
b	1.03	Regional Battlers	2.2
c	1.08	Ageing Regional Towns	15.3
d	1.09	Struggling Country Towns	9.7
e	1.19	Young Regional Families	9.7
f	1.20	Forestry, Fishing and Farming	2.0
g	1.24	Sheep Runs	1.9
h	1.25	Regional Mix	3.6
i	1.26	Mixed Farming Areas	4.6
j	1.27	Wheat Farming	3.7
k	1.31	Cattle Country	1.3
l	1.31	Rising Country	9.5
m	1.40	Dairy Farming	9.1
n	1.53	Small Farms and Regional Lifestyle	4.3
o	1.59	Thriving Regional Living	13.4
p	1.70	Affluent Coastal Lifestyle	2.8

§ Note the numbers used in figure legends do not correspond with those in Additional file 1.

† Each 0.01 increase in the ratio of weekly household income reflects an additional A$6.50 compared to the lowest earning segment.

Expected numbers of live births with DS were calculated by assigning a maternal age-specific risk at term to each birth in 2002, according to the mother's age. Maternal age-specific risks were taken as published by Reynolds in 1994 [19]. Modelled and aggregated expected and observed numbers of DS were then tabulated for each geodemographic segment and expressed as a proportion of all DS births. Modelling of data resulted in artificially high numbers of observations and it was not feasible to undertake a valid statistical analysis. Consequently, a descriptive approach was taken.

**Figure 1:**
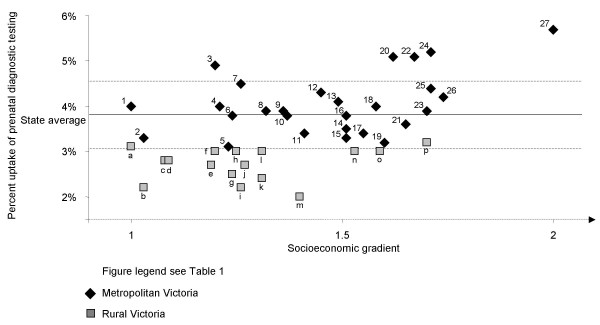
Percent uptake of prenatal diagnostic testing in women aged less than 37 years by geodemographic segment, 2002.

**Figure 2:**
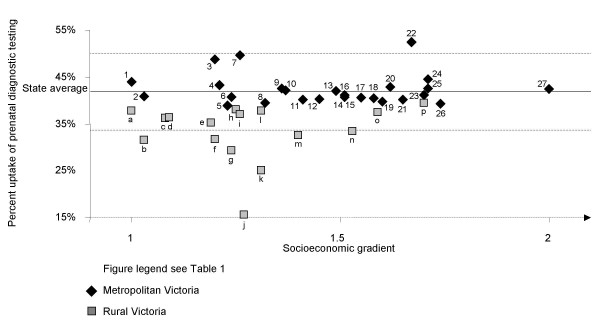
Percent uptake of prenatal diagnostic testing in women aged 37 years and over by geodemographic segment, 2002.

Modelled observed rates of prenatal diagnosis were compared to State average rates (expected) for both maternal age groups and visualised in Figures 1 and 2.

**Figure 3:**
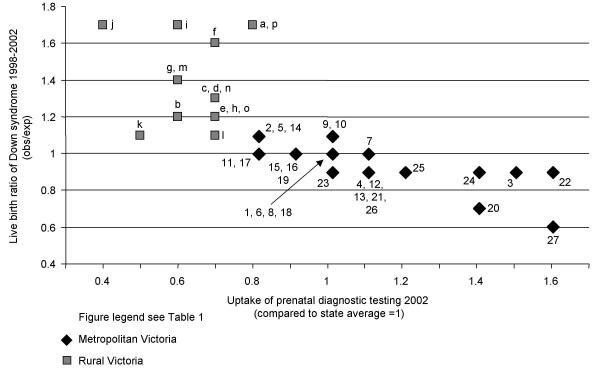
Live birth ratio of Down syndrome by uptake of prenatal diagnostic testing, 2002.

Modelled rates of prenatal diagnosis and confinements (all ages) for each geodemographic segment were expressed as a proportion of all confinements. A ratio for DS for each segment was created, where the observed rate being equal to the expected rate was defined as 1. A ratio above 1 indicated a higher than expected proportion of DS and a ratio below 1 indicated a lower than expected proportion of DS. This ratio was defined as the live birth ratio of DS. Similarly, a ratio for prenatal diagnosis was derived by comparing observed rates of uptake with the State average (all ages). Uptake of prenatal diagnostic testing was plotted against live birth ratio of DS as depicted in Figure 3.

## Results

### Description of data

Baseline data from the PDCU show that there were 61,080 births in 1998 and 61,883 births in 2002. Our study sample included 166 and 180 babies born with DS for the years of 1997–1999 and 2000–2002 respectively (Table 2). 9.5% of women giving birth in 1998 were aged 37 or over and this proportion rose significantly by 2002 to 11.7% (p < 0.001) (Table 2). The proportion of women aged 37 years and over who gave birth to a baby with DS rose from 27.7% in 1998 to 32.8% in 2002, a non-significant increase (p = 0.30). When the advanced maternal age cut off was set at 35 years as it is done in a number of other countries, 35.5% of babies with DS were born to mothers aged 35 years or older in 1998, increasing significantly to 46.7% in 2002 (p = 0.04, not shown).

**Table 2:** Distribution of all births and births of babies with DS, by year and maternal age group

	**1998**	**1997–1999**	**2002**	**2000–2002**
	**Births (%)**	**DS Births (%)**	**Births (%)**	**DS births (%)**

	**N = 61 080**	**N = 166**	**N = 61 883**	**N = 180**

**Maternal age group**				
0–34	81.6	64.5	78.6	53.3
35–36	8.9	7.8	9.7	13.9
37–39	6.9	15.7	8.2	17.8
40 plus	2.6	12.0	3.5	15.0
**Total <37**	**90.5**	**72.3**	**88.3**	**67.2**
**Total 37 and over**	**9.5**	**27.7**	*11.7	^NS^**32.8**

^NS ^p = 0.30 for the difference in the proportion between 1998 and 2002

* p < 0.001 for the difference in the proportion between 1998 and 2002

Table 3 shows that women who gave birth to a baby with DS did not differ from the overall cohort of women giving birth in Victoria in their region of birth or marital status in both study periods. Mothers of babies born with DS were more likely to have at least one previous child, and in the later study period, were more likely to give birth in a public hospital (when compared to private) and live in rural Victoria (when compared with metropolitan).

**Table 3:** Distribution of all births and births of babies with DS by year, hospital category, marital status, parity and mother's region of birth

	**1998**	**1997–1999**	**2002**	**2000–2002**
	**Births (%)**	**DS Births (%)**	**Births (%)**	**DS births (%)**

	**N = 61 080**	**N = 166**	**N = 61 883**	**N = 180**

**Marital status**				
No partner	12.3	7.8	12.5	12.8
Married or de facto	87.6	92.2	87.3	86.7
Unknown	0.1	0.0	0.1	0.5
	*p = 0.08		*p = 0.90	
**Parity**				
Primigravid	40.2	28.3	42.0	29.4
Multigravid	59.8	71.7	58.0	70.6
	*p < 0.01		*p < 0.001	
**Region of mother's birth**				
Australia	76.1	75.3	76.3	71.7
Overseas	23.7	24.7	23.5	28.3
Unknown	0.2	0.0	0.2	0.0
	*p = 0.81		*p = 0.15	
**Hospital category**				
Public	74.3	73.5	68.1	75.6
Private	25.7	26.5	31.9	24.4
	*p = 0.81		*p = 0.03	
**Region of mother's residence**				
Rural	27.7	25.3	26.3	32.8
Metropolitan	72.3	74.7	73.7	67.2
	*p = 0.49		*p = 0.05	

* p value for the difference between the proportion of babies born with DS and all births

The 2002 State average uptake of prenatal diagnosis in women aged less than 37 years was 3.8% (Figure 1). There were marked differences in the rates of uptake between metropolitan and rural geodemographic segments, ranging from 3.1% (Young Single Parent Families) to 5.7% (Asset Rich, Income Rich) and from 2.0% (Dairy Farming) to 3.2% (Affluent Coastal Lifestyle) respectively (Figure 1). The 2002 State average uptake of prenatal diagnosis in women of advanced maternal age (37 years and over) was 42.2% (Figure 2). Uptake in metropolitan Victoria ranged from 38.9% (Young Single Parent Families) to 52.5% (Inner Suburban Lifestyle Seekers) and in rural areas it ranged from 15.5% (Wheat Farmers) to 39.4% (Affluent Coastal Lifestyle). Most point estimates differed markedly from State average and there was no apparent association between increasing SES and levels of uptake, although the four highest uptake rates in younger mothers were seen amongst the eight highest SES segments of metropolitan Victoria. The level of prenatal diagnosis in younger mothers reflects uptake of prenatal screening or diagnostic testing paid for through the private health care sector [22].

Figure 3 depicts uptake of prenatal diagnosis across all ages compared to State average (8.4%) plotted against the modelled live birth ratio of DS. In the metropolitan segments, the live birth ratio of DS mostly lies within 20% of the expected rate with only two outliers, one of which was 40% lower than expected (Asset Rich, Income Rich). The other (Older Money & Asset Rich) was 30% lower than expected. Ten of the 16 rural segments however, show a live birth ratio of DS of >20% higher than expected. Figure 3 also shows an almost universal inverse relationship between rates of uptake of prenatal testing and live birth ratio of DS.

## Discussion

### Uptake of prenatal diagnosis (Figures 1 and 2)

Given that the results were based on modelled data, extrapolated from actual, we discuss only those segments with rates greater or less than 20% variation from State average, (above or below the line in figures) for each region.

Typical residents of the four metropolitan segments with the highest uptake in younger women (Asset Rich, Income Rich, Rising Wealth, Inner Suburban Lifestyle Seekers and Older Money & Asset Rich) are high income couples with or without children who are at least twice as likely to have a household income of A$100,000 per year compared to the rest of the population. They live in the inner suburbs, either own their home outright or are paying high mortgages and drive late model luxury cars. They have private health insurance and spend around 20% more on specialist physician's fees than the average Australian household. For women aged 37 years and over who have access to testing through the public system, increasing SES had no additional bearing on uptake rates. The "Inner Suburban Lifestyle Seekers" was the only segment of the four with a considerably higher than average uptake of prenatal diagnosis in women of advanced maternal age (52.5%).

In contrast, there were four high income segments in younger mothers that did not show above average uptake rates of prenatal diagnosis. Reasons for this were that the segments were defined by an over representation of professionals, associate professionals, advanced clerical and service workers (Moving Up), families with teenage children (Comfortable and Owned Outright, Mature Families) and couples aged 40–44 years old with young children aged around 5 to 14 years (Professionals with Young Families). In addition, people living in these segments also subscribe to private health insurance but do not spend extra money on specialist fees when compared to all of Australia.

There was a marked peak in the uptake rate by younger women in one lower socioeconomic rank (High Rise Rentals, 4.9%). As high rise rentals in metropolitan Melbourne are interspersed throughout the wealthy inner suburbs, the finding of a high uptake rate in women living in this segment is most likely related to an artefact of the data modelling, rather than an actual characteristic of this group. The segment is defined by a large proportion of residents born overseas (40%), with over 60% having arrived in Australia in the last ten years and a strong skew towards the 20 to 34 year old age groups. They are 30% less likely to have private health insurance than the average Australian.

There were also relatively high prenatal diagnosis uptake rates for both age groups in the "Vietnamese Migrant Enclaves". Although these did not vary more than 20% from State average, the peaks may be explained by the fact that there is a well established metropolitan community based antenatal clinic for Vietnamese women [20] and by a tendency to accept prenatal testing as advised by their doctors [21].

Uptake of prenatal diagnosis in both maternal age groups in rural areas was universally low and most segments were well below 20% less than the State average in the younger women. Segments such as "Affluent Coastal Lifestyle", "Thriving Regional Living", "Rising Country" and "Regional Mix" are predominantly located in regional centres and access to services may have played a role in their slightly higher rates of uptake, particularly in the older women. The "Regional Battlers" segment, while also defined as located in regional centres, mainly consists of one parent families, separated or widowed people and residents aged over 60. Household income and level of education are well below average and a high proportion of households do not have a car. Residents in this segment are 31% less likely to spend money on specialist physician's fees than average. The issue of access to testing for women in this segment more likely relates to a lack of individual opportunity, than their physical location.

Women residing in the heartland of farming in Victoria (Dairy Farmers, Wheat Farmers, Cattle Country and Sheep Runs) had the lowest rates of uptake of testing. Most residents in these areas were born in Australia and married couples with children are over represented. Although there is low unemployment, generally household incomes are below average. While private health insurance cover is common, people living in farming areas spend little on specialist physician's fees compared to the rest of Australia.

Interestingly, patterns of utilisation of prenatal diagnosis in rural women were not uniform in both maternal age groups. "Dairy farmers" had the lowest rates of prenatal diagnosis in the younger women, whereas older women in "Wheat farming" areas had exceptionally low rates of uptake. A similar discordance was observed for the "Mixed Farming" segment, which was very low in the younger women. Households with more than five people are over represented in farming areas and low uptake of testing in older mothers and, to an extent, in the younger mothers may be explained by a previous finding that women of higher parity are less likely to have testing [8]. A combination of this and access to testing, possibly further contribute to low uptake in younger women in the "Dairy Farming", "Mixed Farming" and "Cattle Country".

### Down syndrome live birth ratio (Figure 3)

We observed an almost universal inverse relationship between utilisation of prenatal testing and live birth ratio of DS. This is not surprising as approximately 75% of cases with DS are diagnosed prenatally in Victoria [22] and studies have shown that most pregnancies are terminated following diagnosis [23,24]. However, two of the four highest rates of testing in metropolitan Victoria did not result in equally low rates of babies born with DS. Women in the metropolitan "Rising Wealth" and "Inner Suburban Lifestyle Seekers" segments showed a high overall uptake of testing in Figure 3, whereas the live birth ratio of DS was less than 20% lower than expected. This finding is difficult to explain within the constraints of the data available, but an underlaying skewed maternal age distribution and the associated risk for DS may be a contributing factor. Table 2 shows that by 2002, one third of all babies with DS were born to the high risk but relatively small group of women of advanced maternal age and it is possible that testing is not reaching this particular high risk population in the most effective way. It has certainly been shown that prenatal screening is a more effective filter for prenatal diagnosis than advanced maternal age alone [22]. Consequently, the relationship between uptake of prenatal diagnosis and live birth rate of Down syndrome may be affected by the extent to which women use prenatal diagnosis with or without consideration of screening results [25]. In addition, a contribution to the departure from the general inverse relationship between uptake of prenatal diagnosis and live birth ratio of DS may be that some women in these segments are more likely than others to continue with their pregnancy in the event of a positive test result.

Table 3 shows that, in 2002, there were proportionally more babies born with DS in rural areas when compared to the overall birth rate. This prevalence appears to be directly related to levels of uptake of prenatal diagnosis (Figure 3), in particular in the farming segments. The live birth ratio of DS was highest in wheat and mixed farming areas with a rate 70% higher than expected. Only 26% of people in wheat and mixed farming areas and 31% in sheep farming areas live in inner regional districts and lack of access to services may partly contribute to these findings. The "Low income and ageing coastal" and the "Affluent coastal lifestyle" segments also showed a live birth ratio of DS of 70% higher than expected. These segments are at either end of the socioeconomic scale but largely co-exist in similar geographic locations and without a more specific geographic identifier, it is difficult to clarify how the characteristics of the segments may contribute to these findings.

In summary, the lack of statewide geodemographic consistency in uptake of prenatal diagnosis implies that there is a need to target health professionals and pregnant women in specific areas to ensure there increased equity of access to services and that all pregnant women can make informed choices that are best for them. Equally as important is the increased opportunity for reproductive choice and provision of appropriate health services for families of children with Down syndrome. Our findings show that these potential interventions are particularly relevant in rural areas.

## Limitations and strengths

The main limitation of this study lies in the geographic identifier. The data collections did not hold street addresses and as a consequence, our results are derived from modelled data. A small number of our findings, which may be counterintuitive or have no apparent explanation, may be due to an artefact of the modelling (eg high rate of uptake of testing in "High Rise Rentals"). We emphasise the importance of appropriate data collection (individual level street address) before this framework can be promoted as a contribution to epidemiological methodologies.

However, even if street addresses had been available, the geodemographic software system only allows for the study of groups of people. Effects of individual level socioeconomic factors cannot be disentangled from those factors related to place of residence. Our findings need to be interpreted within these limitations and inference about the behaviour and characteristics of individuals cannot be drawn.

## Conclusion

We have provided a plausible geodemographic framework for examining the uptake of prenatal testing at a population level and have put this framework into a broader context of the live birth prevalence of DS. Adaptation of techniques previously used in business consumer behaviour modelling, allowed for the development of insights into health behaviour in a cost-effective manner without extensive and costly surveys or cohort studies. This methodology uses data that are already available to health service organisations, the analysis is in line with the primary purpose of collection and can be used to identify variations in service use and health outcomes. In addition to the basic descriptors used in this study, a wide range of data are available for each geodemographic segment, which allows for greater understanding of health behaviour than regression analysis of traditional socioeconomic indices of disadvantage. Information about smoking behaviour, food and alcohol consumption, the likely use of web-based resources or the type of magazine or newspaper preferred by consumers in a defined geodemographic lifestyle segment may in future prove invaluable to focus health promotion programs and to allow for effective allocation of resources.

## Competing interests

EEM and JLH; none declared. DMcC holds stock in Pathfinder Solutions (Aust.) Pty Ltd, the developers of the Segments of Australia geodemographic classification, and is currently conducting research using the Segments of Australia classification. Pathfinder Solutions has received payment from the Murdoch Childrens Research Institute for use of this classification.

## Authors' contributions

EEM conceived the study, participated in the design, collected and cleaned the data, completed the analysis and led the writing. DMcC participated in the design and performed the geodemographic analysis. JLH assisted with the study, interpretation of results and critical review of the manuscript for intellectual content.

## Pre-publication history

The pre-publication history for this paper can be accessed here:



## Supplementary Material

Additional file 1 Pathfinder Segments of Australia (© Pathfinder Solutions (Australia) P/L). The file provides basic descriptions of the geodemographic segments used in the analysis. Click here for file
