# Supplementary material for: Health behaviour modelling for prenatal diagnosis in Australia: a geodemographic framework for health service utilisation and policy development
Source: BMC Health Serv Res. 2006 Sep 1;6:109. doi: 10.1186/1472-6963-6-109 (PMC1574302; doi:10.1186/1472-6963-6-109)
Supplement: Additional file 1 — Pathfinder Segments of Australia (© Pathfinder Solutions (Australia) P/L). The file provides basic descriptions of the geodemographic segments used in the analysis. [file 1472-6963-6-109-S1.pdf]

Pathfinder Solutions © Copyright 2003 /// phone: +61-3-9670 3030 /// email: info@pathfindersolutions.com.au

PATHFINDER SEGMENTS OF AUSTRALIA

01 Asset Rich, Income Rich

The top of the tree

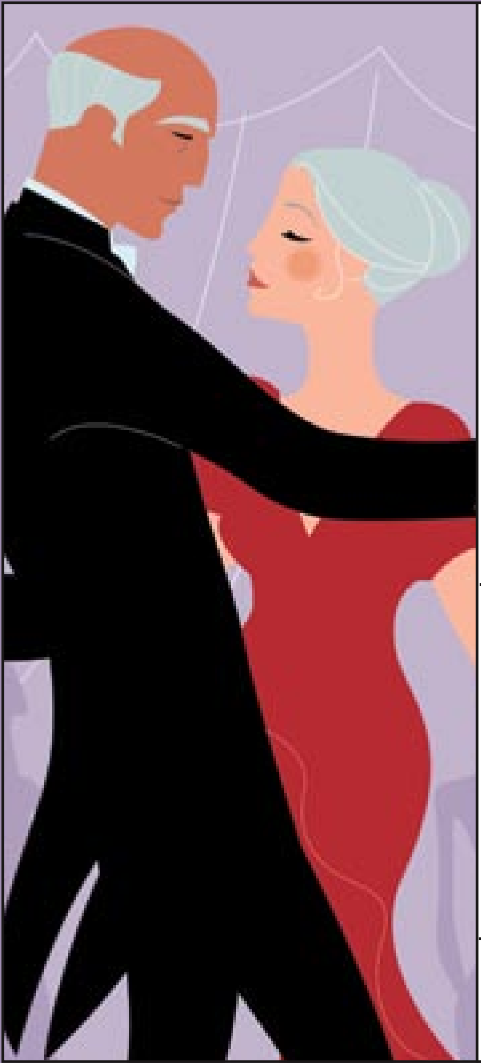

Segment Description

The crème de la crème of consumers, this is the market segment that luxury brand marketers salivate over; all marketers for that matter!

Individuals are four times more likely to have incomes of $75,000 or more per year, and they are asset rich too, with more than half owning their homes outright. We're not talking just your everyday mansion here; many have also been extended or renovated ten to twelve years ago. As expected, luxury cars are vastly over represented, and you are more than 3 times as likely to find drivers of late model vehicles such as Bentley, BMW, Jaguar, Mercedes Benz and Volvo here.

This is an e-savvy bunch with over half using the Internet daily (probably to check the state of their stocks). Not surprisingly, the old school tie is prevalent with over half having attended a non-catholic private school. Around a quarter have then gone on to University, which shows the proverbial piece of paper's not all you need to make it big.

This segment is three times more likely to read the Fin Review and twice as likely to be a Qantas Frequent Flyer. In fact, they love a trip O.S. especially to UK/Ireland, Europe, Canada/USA and Asia. They have a healthy interest in skiing, sailing, wine collecting, classical music (44%), live theatre and rugby union. They are also above average spenders on Pay TV subscription.

Defining Features

1. Four times the average level of personal income above $1500 per week
2. 95% of homes in this segment are within the top 10% of Australian house values
3. More than half of all homes are owned outright
4. More than a quarter of people over 15 y.o. have university qualifications

Key Expenditure

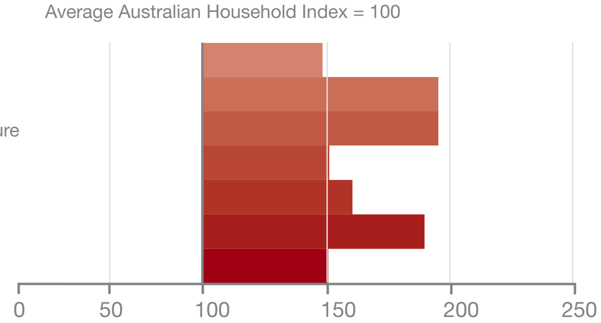

Population Profile

98% of the 751,410 people who live in this segment, live in the Major Cities.

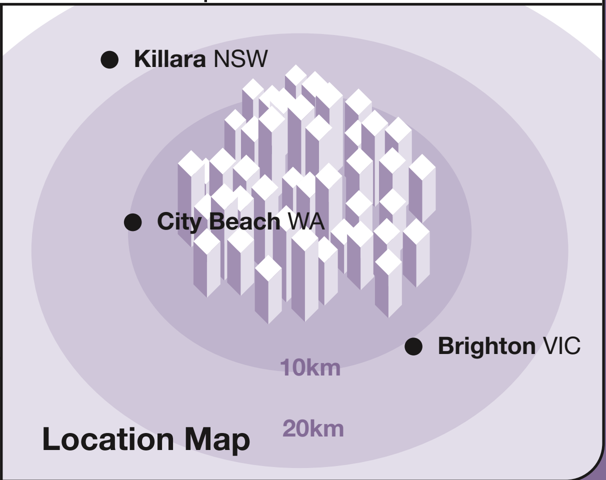

Location Map

Pathfinder Solutions © Copyright 2003 /// phone: +61-3-9670 3030 /// email: info@pathfindersolutions.com.au

PATHFINDER SEGMENTS OF AUSTRALIA

02 Affluent City Apartment Dwellers

Living the high life

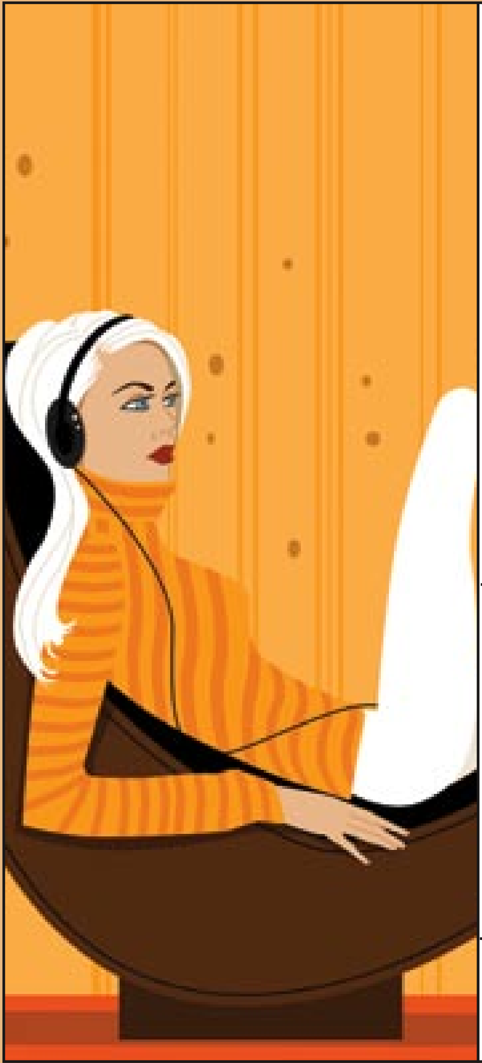

Segment Description

This confident young crowd can be found nestled in and around the capital cities. They reflect a change in the style of living in Australia. This is, however, as much a result of 'developers dollars' and government policy encouraging high-density living as it is a desire to live close to the action.

In terms of income, individuals are three times more likely to have incomes of $75,000 or more per year, and twice as likely to have incomes in the $50,000-$75,000 bracket. Luxury cars are over represented, and you are twice as likely to find drivers of late model vehicles such as BMW, Jaguar, MG, and Porsche here.

A learned clan, this group has the highest level of tertiary qualifications in Australia (65%). More than one third of the men have jobs in finance, insurance and business services and around three quarters are employed in professional, managerial or associate professional roles. Single person households and childless couples are the norm here, representing almost 80%, with only 8% of households being group or shared living arrangements.

These guys are so close to the action they frequently use public transport. You'll find them reading the Fin Review, BRW, Time and travel and finance magazines.

Defining Features

1. Weekly household incomes are strong in the $75,000+ p.a. range
2. 75% of households live in apartments
3. 80% of households are singles or couples only
4. This segment has the highest levels of tertiary education in Australia

Key Expenditure

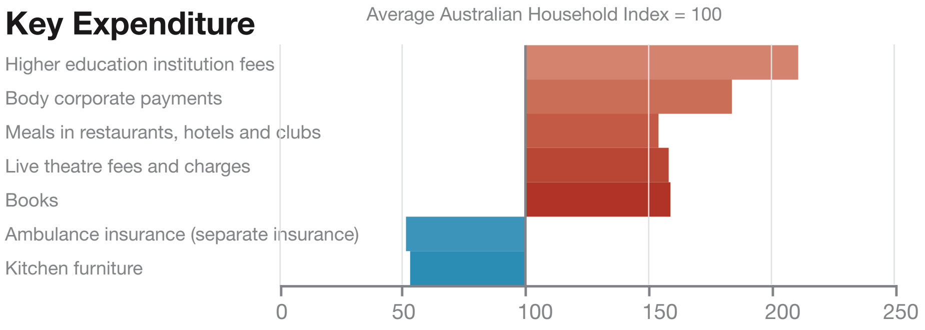

Population Profile

99% of the 263,869 people living in this segment live in Major Metropolitan Cities.

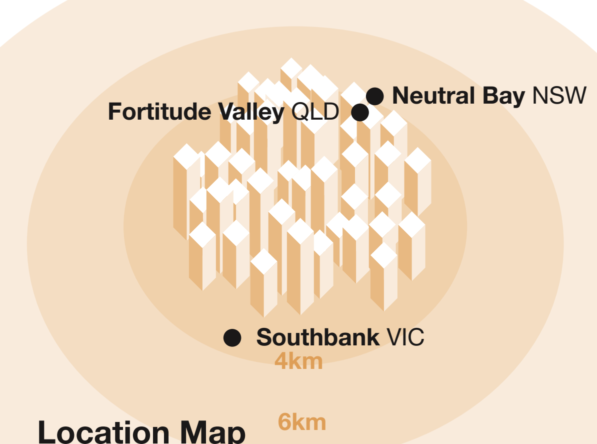

Location Map

Pathfinder Solutions © Copyright 2003 /// phone: +61-3-9670 3030 /// email: info@pathfindersolutions.com.au

PATHFINDER SEGMENTS OF AUSTRALIA

03Prestige New Housing

Nouveau riche

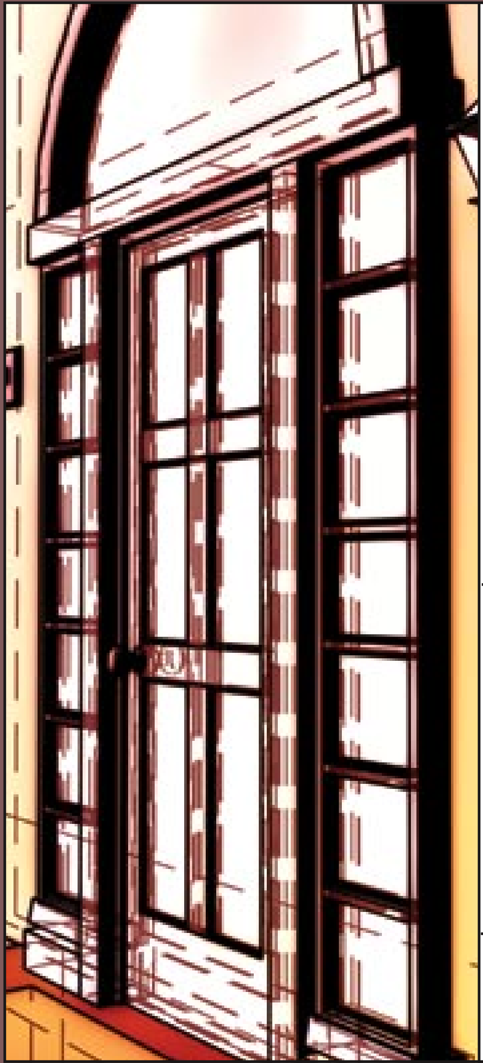

Segment Description

This is predominantly the suburban elite. Think brand-new mansions with pillars, think golf, think triple driveways and swimming pools. We're talking serious mansions here. Over 70% of homes in these areas are in the top two deciles for home value. We're also talking seriously wealthy. A significant number of households have two or three motor vehicles. Household income in this segment is twice as likely to be above $75,000 per year.

There is a very even mix of car types in this segment, and despite the relatively healthy level of household income, there is not the same presence of late model luxury cars as is found in segments one or two. Big spenders, they have a high level of investment home loans and spend lots on credit cards (always paid off in full every month, no worries there) and the second highest level of share ownership.

This group includes a large proportion of migrants with around 20% of people in these areas speaking a language other than English at home. A high proportion is Chinese with around 6% speaking that language at home.

They are partial to eating out, going to the movies and fashion clothing. Readership of the BRW and Sun Herald is above average as is their purchasing over the Internet and membership of the Qantas Frequent Flyer program.

Defining Features

1. Majority of the homes were built between 1994 and 1999
2. 20% of people speak a language other than English at home and the proportion of people speaking Chinese has increased by 77% from 1991 to 2001
3. Over 70% of homes fall in the top 2 deciles for home value
4. A significant number of households have 2 or 3 motor vehicles.

Key Expenditure

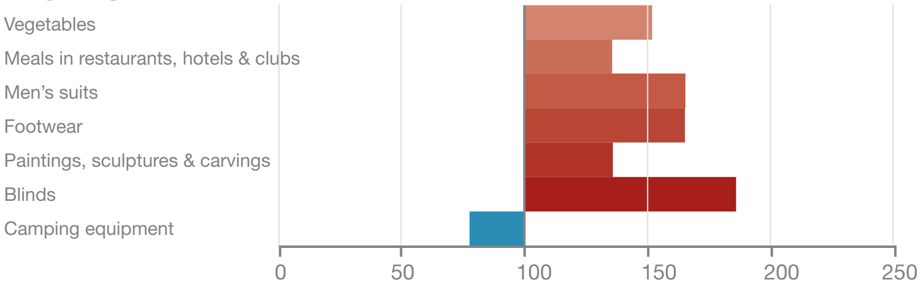

Population Profile

89% of the 227,276 people who live in this segment, live in Major Cities

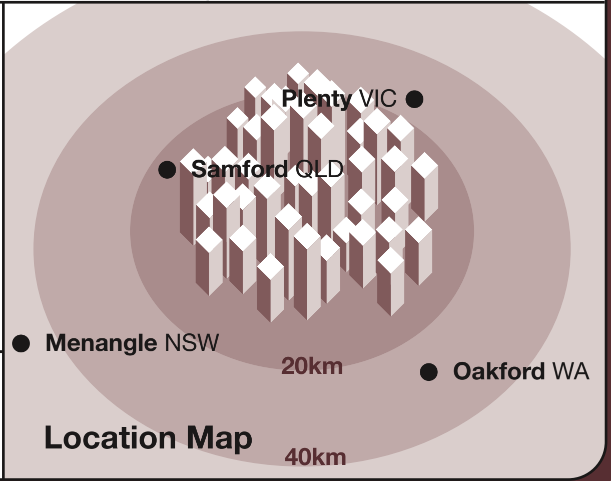

Location Map

Pathfinder Solutions © Copyright 2003 /// phone: +61-3-9670 3030 /// email: info@pathfindersolutions.com.au

PATHFINDER SEGMENTS OF AUSTRALIA

04 Inner Suburban Lifestyle Seekers

Movers and shakers

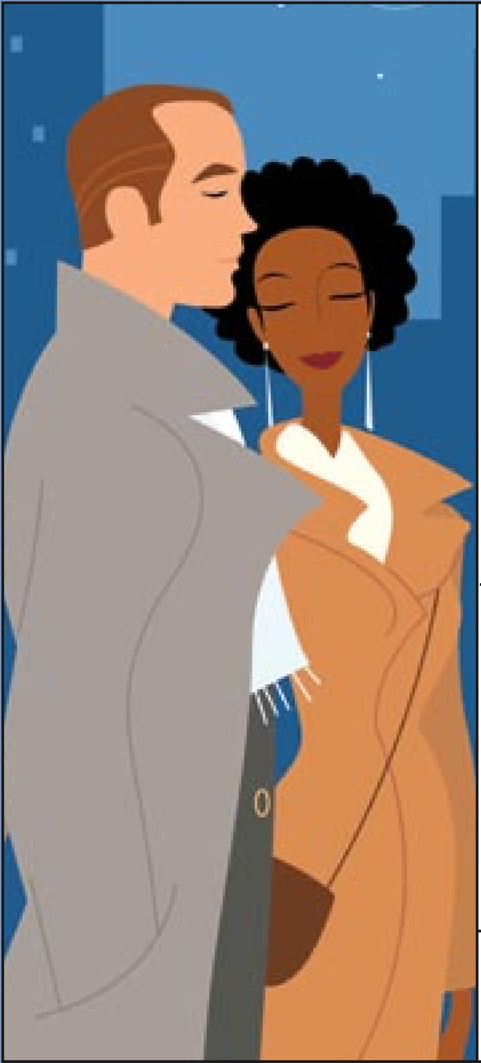

Segment Description

Here we're talking hip, happening, high-income couples and group households (15%) living in the inner suburbs of the capital cities. Over 40% are renting and they mostly live in groovy, old houses and apartment blocks predominantly built before the 1930s. Household income in this segment is twice as likely to be above $100,000 per year, and they spend their money on eating out, drinking, clothes, parking fines, making alterations to their homes and the finer things in life such as the arts, gourmet food and wine collecting.

Late model luxury cars are over represented, and you are twice as likely to find drivers of late vehicles such as Alpha Romeo, Citroen and MG here. Most are aged between 25 and 39 with around 10% being born overseas and having arrived in the last 2 years. They are e-savvy and use public transport and telephone banking. Employment in cultural and recreational services is high with around 8% working in cafes, restaurants and accommodation.

This segment is over represented in the proportion of home businesses providing professional services, with twice the national rate of home professional businesses found here. This is a forceful, independent bunch who have no need for religion (around 20% have no religion) or a boss. Don't try to tell them what to do; they'll do it their own way thanks!

Defining Features

1. Weekly household incomes are double the national rate in the$100,000 + p.a. range
2. Group households make up almost 15% of all households, with a strong skew to the 25-39 age bands
3. Around half the homes are semi-detached terraces or flats and apartments
4. These areas contain the highest proportion of homes built before 1911, and also contain many houses built before the 1930's.

Key Expenditure

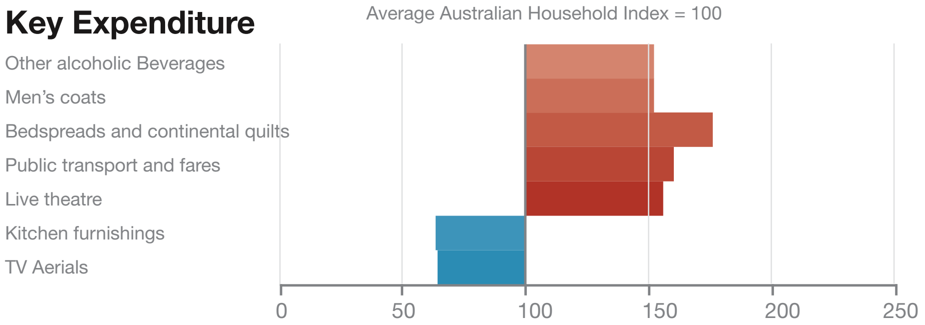

Population Profile

All of the 498,303 people living in this segment live in Major Metropolitan Cities

Location Map

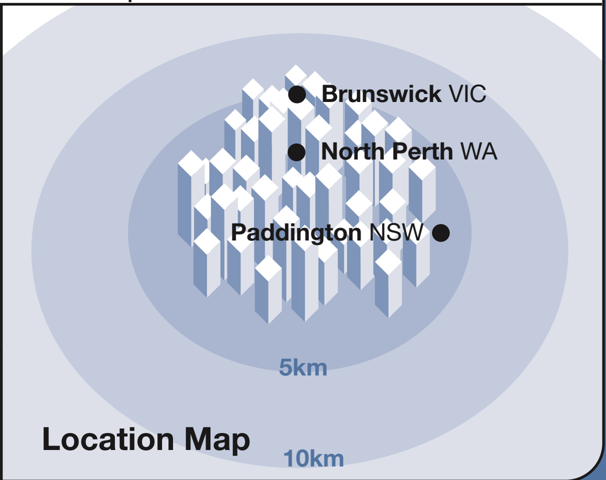

Pathfinder Solutions © Copyright 2003 /// phone: +61-3-9670 3030 /// email: info@pathfindersolutions.com.au

PATHFINDER
SEGMENTS
OF AUSTRALIA

05Rising Wealth

Upwardly mobile

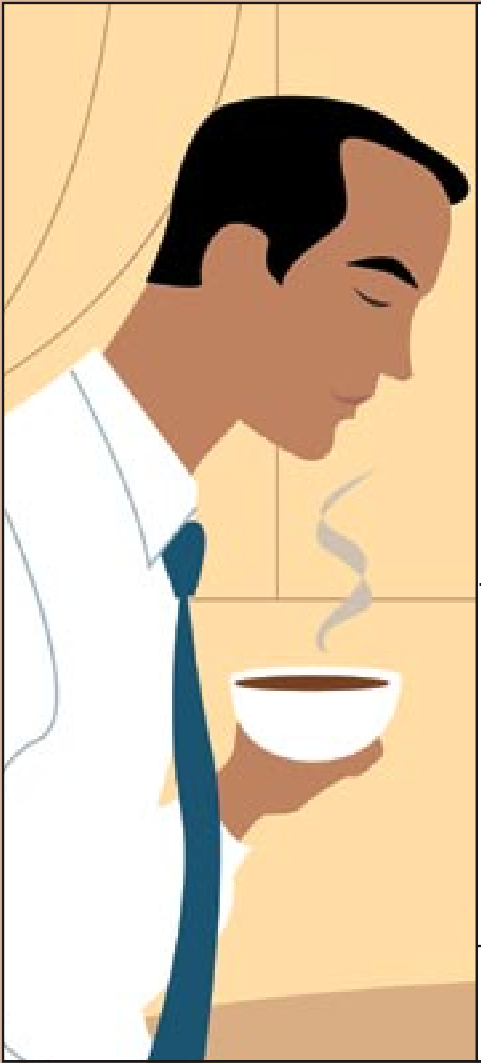

Segment Description

Here we're mainly talking about areas in the major cities which over the past ten years have had a serious boom in residents with incomes in the top bracket.

Essentially, for whatever reason, these areas suddenly became the place to be.

Most of the households are either high income couples or high income couples with kids (the old DINKS), and many have already renovated/extended their house (in the last ten years) and its worth over half a million dollars (average $578,000).

This segment is twice as likely to contain households that earn in excess of $100,000 per year. Their mortgage is healthy at around $1800 a month. Late model luxury cars are over represented, and you are twice as likely to find drivers of vehicles such as the Bentley and Lexus here.

Every year they go overseas on holidays and when they are at home they love to attend an event - the tennis, the footy grand final or any other big event. Dad probably plays golf, Mum does yoga.

Health cover is not a concern as access to these facilities is a priority and finances are not a worry.

Defining Features

1. Over the last 10 years this segment has had a substantial increase in people earning incomes in the top income quartiles
2. This segment has two times the average level of household income above $104,000 p.a.
3. The average house value is $578,000, with over a 3/4 of all households having mortgage repayments of $1,800 per month

Key Expenditure

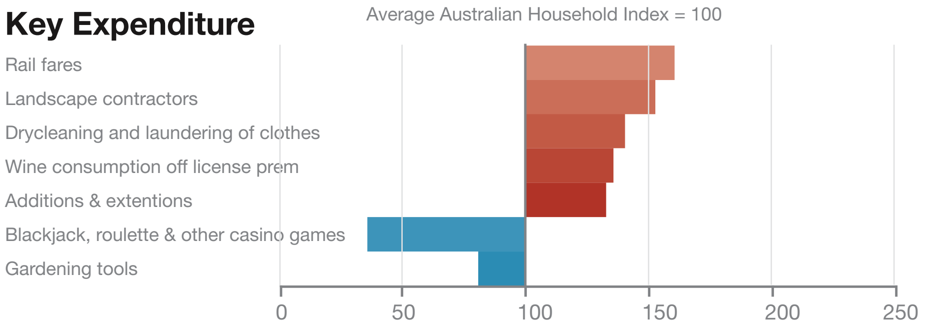

Population Profile

100% of the 223,490 people who live in this segment, live in Major Cities

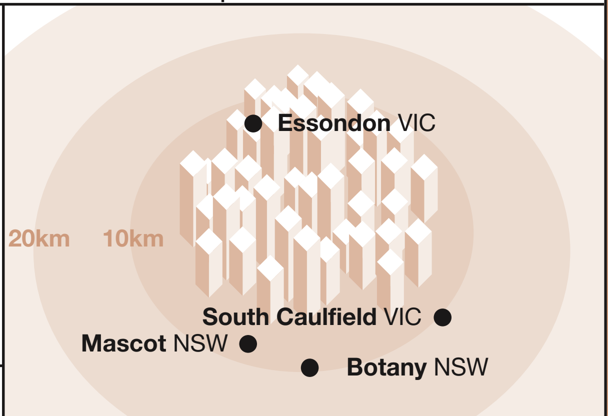

Location Map

Pathfinder Solutions © Copyright 2003 /// phone: +61-3-9670 3030 /// email: info@pathfindersolutions.com.au

PATHFINDER SEGMENTS OF AUSTRALIA

06 Older Money & Asset Rich

Solid wealth

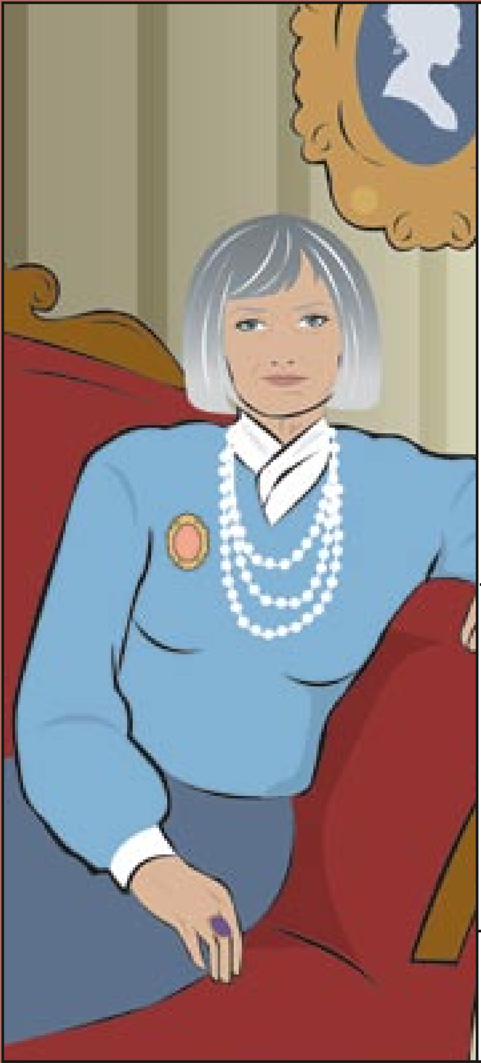

Segment Description

When you stop working, hopefully you'll be as comfortably off as these guys. Although, they may appear wealthier than they are.

Many are likely to be on the pension but this segment has the highest asset value of all people over 65. Around 20% of people in this segment are aged over 65, and the proportion of people over 85 is 3 times the national average. Despite the presence of many people aged over 65, this segment is also twice as likely to contain households that earn in excess of $100,000 per year.

To paint a picture, this is old money – the lady throwing the charity ball at her manor, the old gentleman sponsoring the rowing team at his old grammar school. They are twice as likely to have sent their kids to private schools and are probably now living alone, with one in three of all households being single person.

You are five times as likely to find drivers of late model luxury cars here.

They have downsized to flats, apartments and townhouses and spend much of their cash on body corporate payments and soft furnishings, as well as clothes, medicines and wine. Interestingly, this segment has the second highest rate of home businesses providing professional services in areas such as law and accountancy.

Defining Features

1. Around 21% of people in this segment are aged over 65
2. Around half the homes are semi-detached terraces or flats and apartments
3. Approximately 1/3 of all households are single person households
4. Household incomes over $100,000 p.a. are twice as common as the national average

Key Expenditure

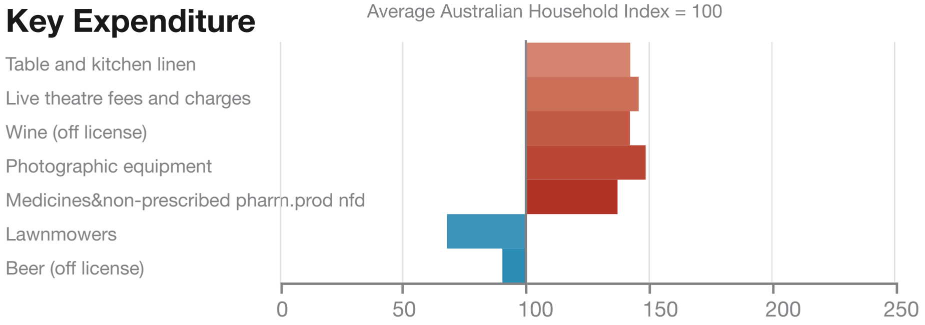

Population Profile

99% of the 387,993 people living in this segment live in Major Cities

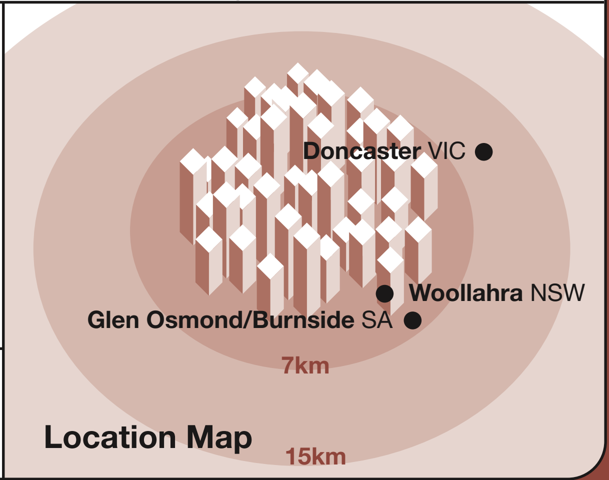

Location Map

PATHFINDER SEGMENTS OF AUSTRALIA

07 Moving Up

Working hard for their money

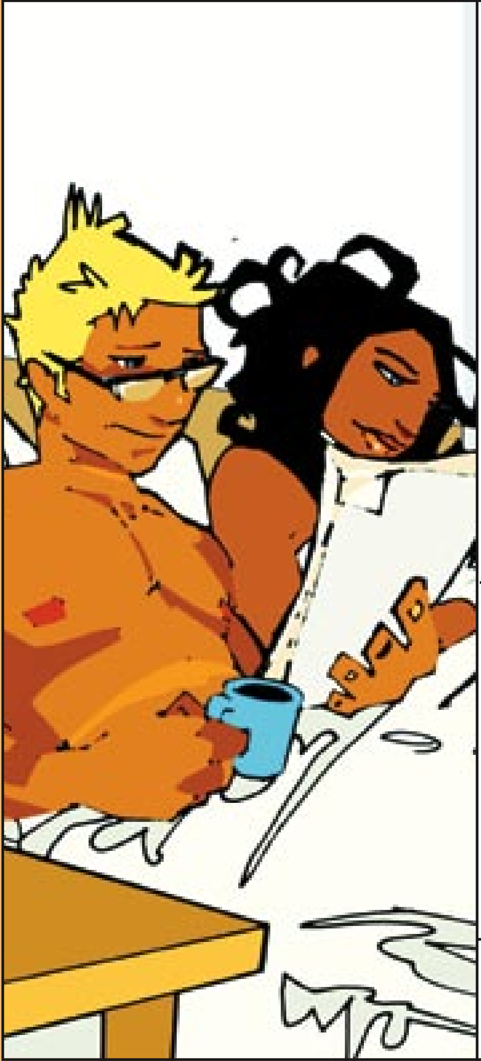

Segment Description

Professionals who are working hard but don't earn quite as much as the Rising Wealth (Segment 5) folk are likely to be in this group.

This segment is 1.7 times as likely to contain households earning over $100,000 per year. And their houses aren't as ostentatious, although around half are in the top three value deciles. Mind you, since 1991, the proportion of households in the top income quartile has increased by 24%, while the bottom quartile has decreased by 33%. This can probably be put down to changes in age distribution in these areas (the proportion of people over 65 decreased by 30 odd % between 1991 and 2001).

There appears to be an even distribution of motor vehicles in all classes and age.

Professionals, associate professionals, advanced clerical and service workers – these guys are basically the step down from the big shots. But the big shots had better watch out, as these hard-workers will take their place if they're not careful. Feathering the nest is a priority, with expenditure being mainly on jewellery, cleaning services, housekeeping and pets. Professional association and union membership is also a must.

Defining Features

1. Contains a high proportion of professionals, associate professionals and advanced clerical and service workers
2. This segment has 1.7 times the average level of household income above $104,000 p.a.
3. More than half the CCDs in this segment are in the top 3 house value deciles
4. Since 1991, the proportion of households in the top income quartiles has increased by 24%, while those in the bottom quartile decreased by 33%
5. The people aged over 65 has decreased by 29% from 1991 to 2001

Key Expenditure

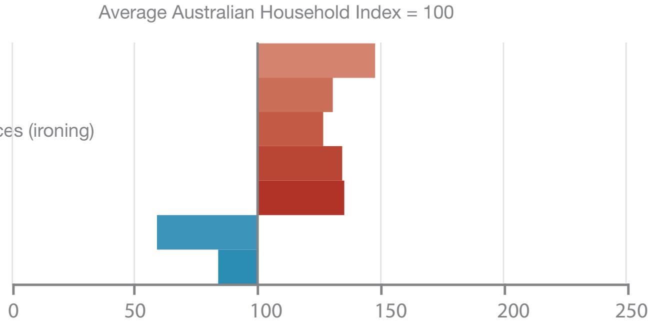

Population Profile

97% of the 307,609 people that live in this segment live in Major Cities

Location Map

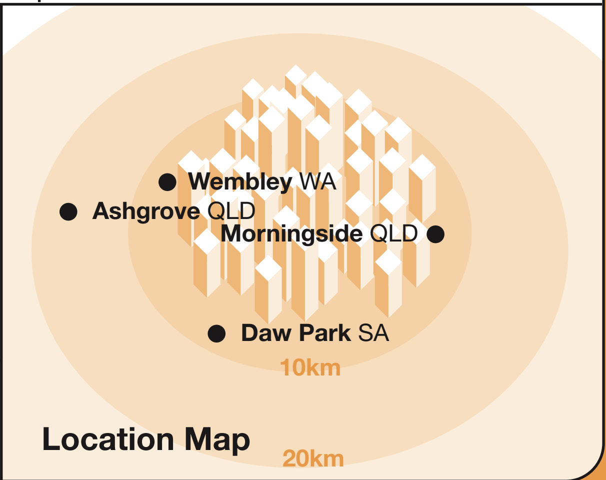

Pathfinder Solutions © Copyright 2003 /// phone: +61-3-9670 3030 /// email: info@pathfindersolutions.com.au

PATHFINDER SEGMENTS OF AUSTRALIA

08 Comfortable & Owned Outright

Sitting pretty

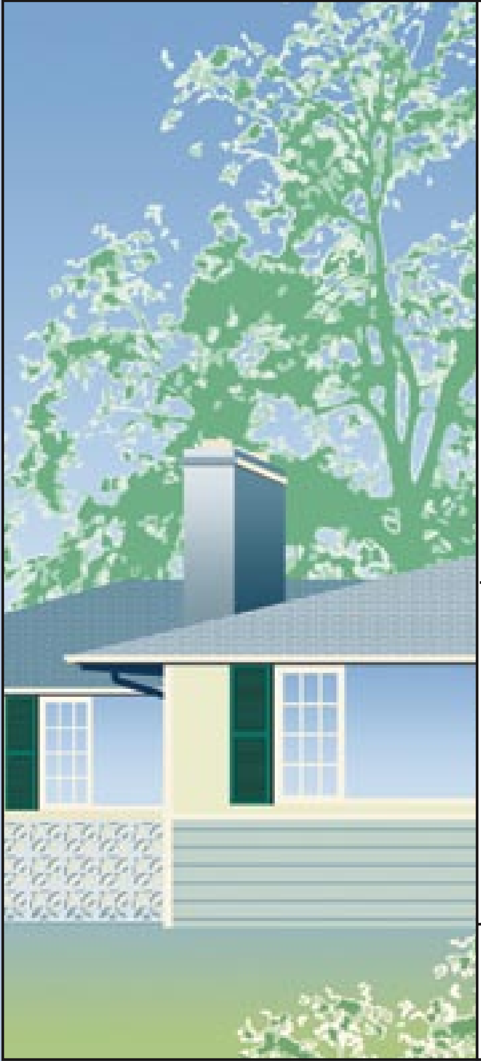

Segment Description

These guys have done their share of hard work and are now kicking back in a fat armchair and enjoying life.

Most are around 50-64 and well over half (57%) own their homes outright. You are nearly twice as likely to find household income levels in excess of $100,000 per year in this segment.

Yes, life's pretty smooth sailing from here on in.

These areas contain lots of families with dependent students aged 15-24 and sometimes full-time tertiary students who love to stay and live off their parents for as long as they can.

Many homes have 2-3 cars and 4-5 people living in them.

These families are really, really comfortable where they are, with over 60% having lived in the same address for over five years. Given all the people and cars it's hardly surprising they spend the odd dollar on carports and home extensions. The parents also spend all their wonderful surplus money on house and contents insurance, cars (surprise, surprise), film and photography (photos of the cars, no doubt) and sports lessons.

Defining Features

1. 57% of homes in this segment are owned outright
2. Over 60% of people have lived in the same address for over 5 years
3. Families with dependent children aged 15-24 are over represented
4. Many households have 2-3 cars and 4-5 people
5. Carports and extensions to family homes are very common

Key Expenditure

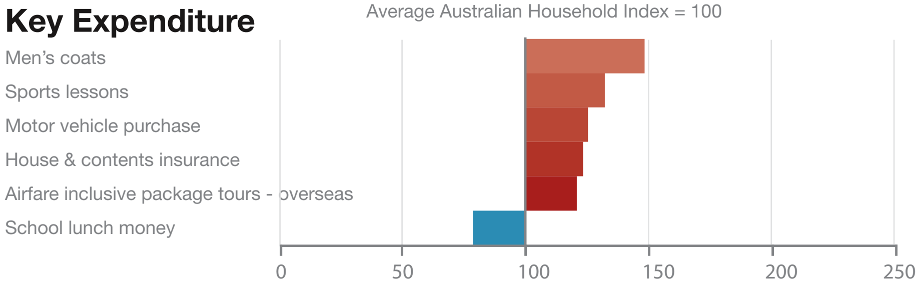

Population Profile

89% of the 390,510 people that live in this segment live in Major Cities and 10% live in Inner Regional areas.

Location Map

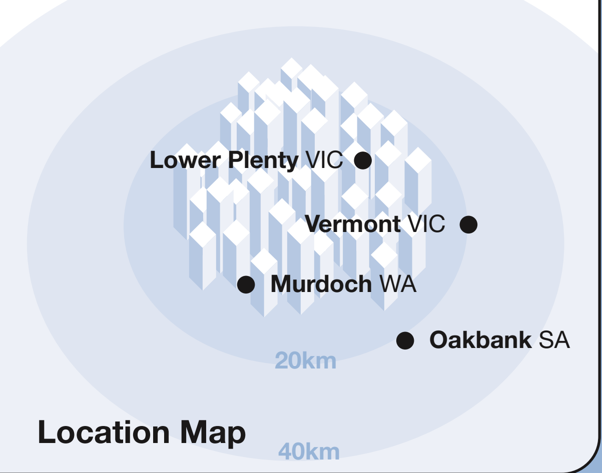

Pathfinder Solutions © Copyright 2003 /// phone: +61-3-9670 3030 /// email: info@pathfindersolutions.com.au

PATHFINDER SEGMENTS OF AUSTRALIA

09 Public Service Heartland

For God and country

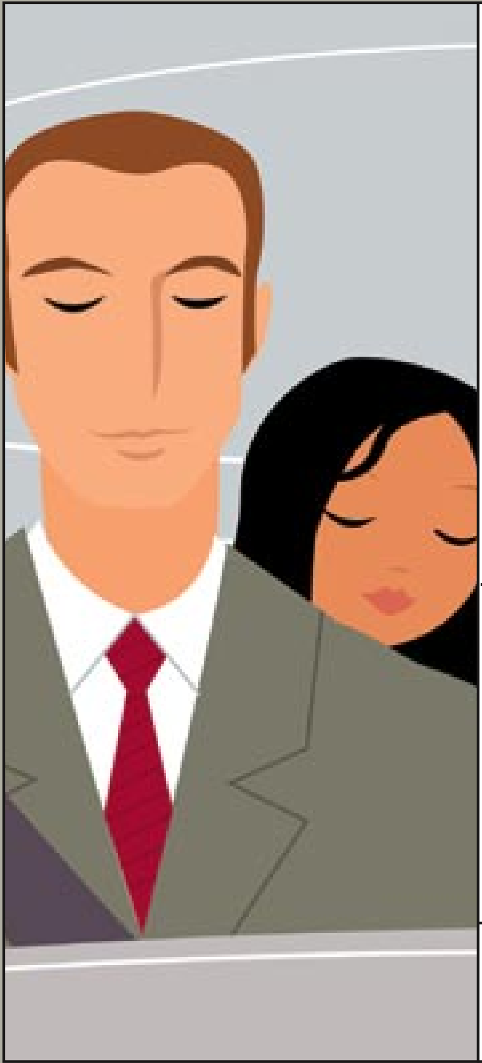

Segment Description

A significant proportion of the workforce is employed in the public sector.

These individuals earn around $40,000-50,000 per year and spend it on alcohol, clothing, footwear, gardening, child-care, home computers, interest on credit cards and donations.

This segment is 1.5 times as likely to contain households that have an income above $100,000 per year.

Located mostly in major cities and some regional areas, most of these people live in their own house and have a mortgage between $40,000 and $120,000. They're not afraid of technology and use computers and the "net" at home and at work.

For some reason Catholicism is prevalent, with use of Catholic primary and secondary schools over-represented by this segment.

Defining Features

1. Incomes are common at both ends of the scale (very high and very low)
2. 85% of dwellings are separate homes
3. Many households have older teenage children
4. More than a quarter of people over 15 years of age, have tertiary qualifications
5. 4 times the national average work in government or defence

Key Expenditure

Average Australian Household Index = 100

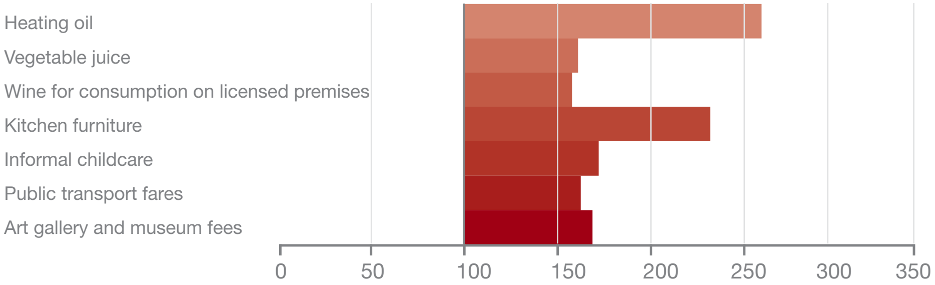

Population Profile

Of the 169,342 people living in this segment, 78% live in Major Cities, 11% live in Inner Regional areas and the remaining 10% live in Outer Regional areas.

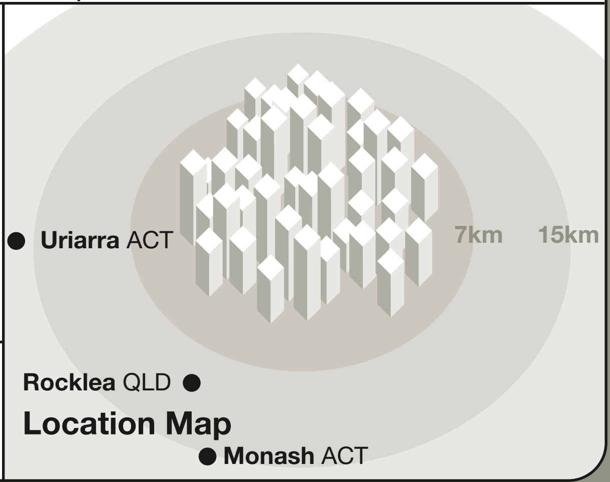

Location Map

Pathfinder Solutions © Copyright 2003 /// phone: +61-3-9670 3030 /// email: info@pathfindersolutions.com.au

PATHFINDER SEGMENTS OF AUSTRALIA

10 Affluent Coastal Lifestyle

Seachange

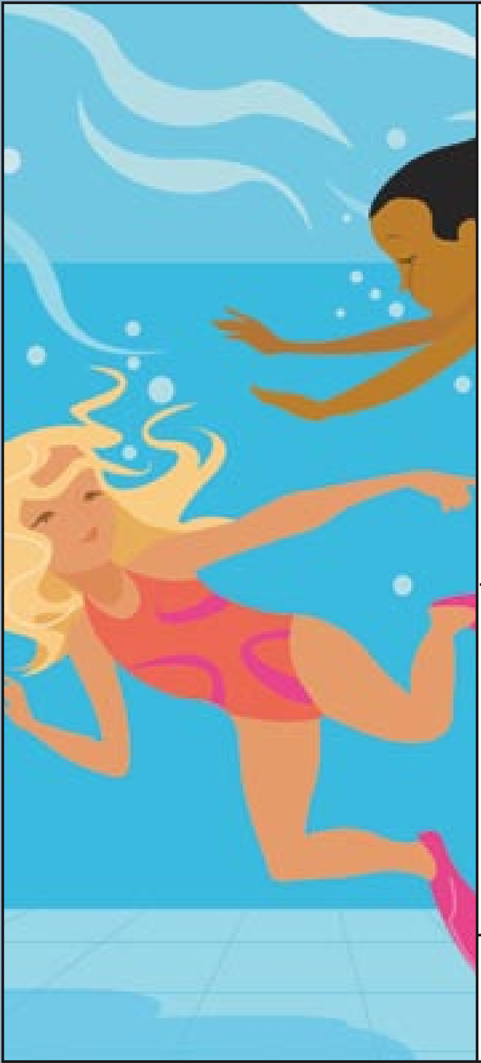

Segment Description

Within a few km's from the coast, these seaside dwellers sure set a high standard, with one in three homes in major city seaside towns being in the top house value decile, with the remainder in the second highest decile.

Median house price is very high and if that's not enough, they have very high rates of home extensions and subdivisions to get the most from their gorgeous properties.

Age groups are generally 10-19 and 40-64 with household income 1.6 times as likely to be in excess of $100,000 per year.

Not only do they have the sea lapping at their gates, but for the past twelve years, these high-flyers have been installing swimming pools at three times the national rate!

Alongside their pools, they usually have 2-3 cars and a few pets, and they typically read newspapers such as the Sun Herald. They send their kids to private schools and for those left over dollars they'll possibly put in a cheeky bet or two at the local TAB.

As you might expect, they have more than a passing interest in surfing and sailing and spending is high on TV aerials.

Defining Features

1. Median house values in this segment are very high with more than 1/3 of homes being in the top house value decile
2. This segment is 3 times more likely than the national rate to install swimming pools
3. Virtually all of these households can be found within a few kilometres of the coast
4. Ages 10-19 and 40-64 are common with few people speaking a language other than English at home

Key Expenditure

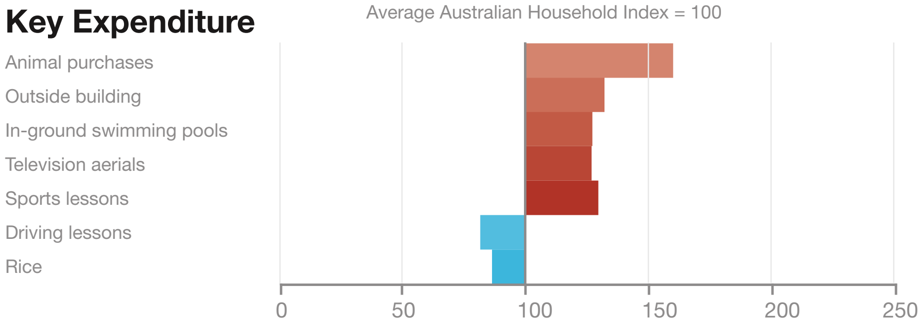

Population Profile

72% of the 355,020 people that live in these area segments live in Major Cities, 20% in Inner Regional and 8% live in Outer Regional.

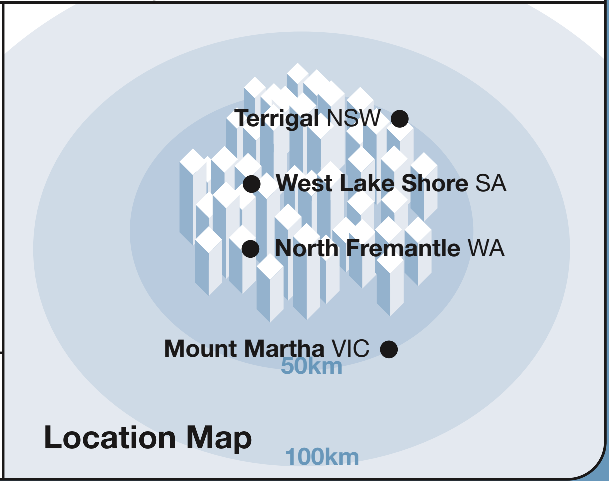

Pathfinder Solutions © Copyright 2003 /// phone: +61-3-9670 3030 /// email: info@pathfindersolutions.com.au

PATHFINDER SEGMENTS OF AUSTRALIA

11 Professionals with Young Families

The Brady bunch

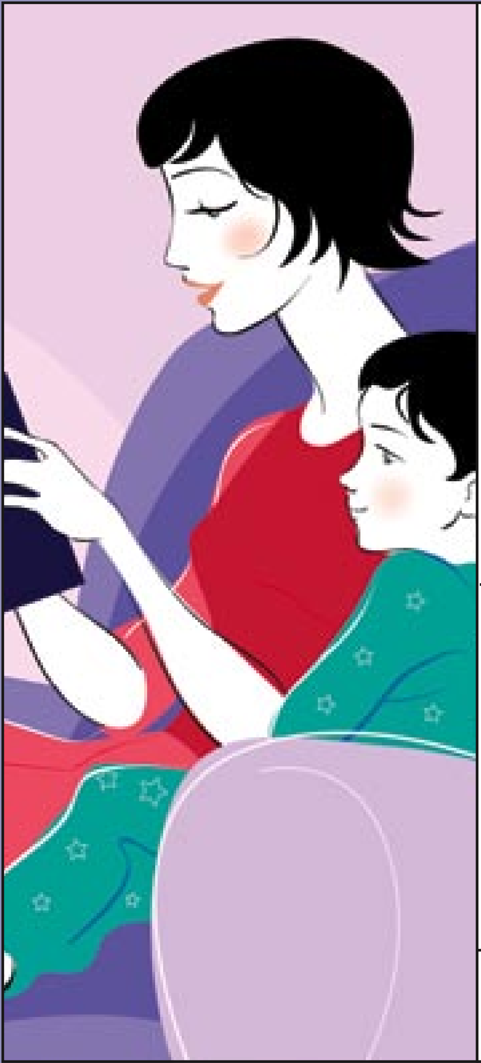

Segment Description

Happy young families - couples around 40-44 years old with younger kids around 5-14 nest around the major cities and inner regional areas. In fact, almost 60% of households are couples with kids.

Their homes were built around the 60s or 80s and are in the leafier suburbs.

They're more likely to have a mortgage than own their home outright (37% are being purchased).

Unemployment is not common and they earn enough to be comfy – around 1.5 times more likely to have a household income above $100,000 per year.

The extra cash these successful couples earn is spent on men's suits, kids' clothes, education, mortgage repayments and Foxtel to entertain the kids.

Defining Features

1. This segment has low levels of employment
2. Age ranges 5-14 years and 40-44 years over represented
3. Around 37% of all homes are being purchased with almost 60% of households being couple families with children
4. This segment has 1.4 times the average level of household income above $62,400 p.a.
5. Many homes in this segment were built in the 1960s, with another burst of construction in the 1980s.

Key Expenditure

Average Australian Household Index = 100

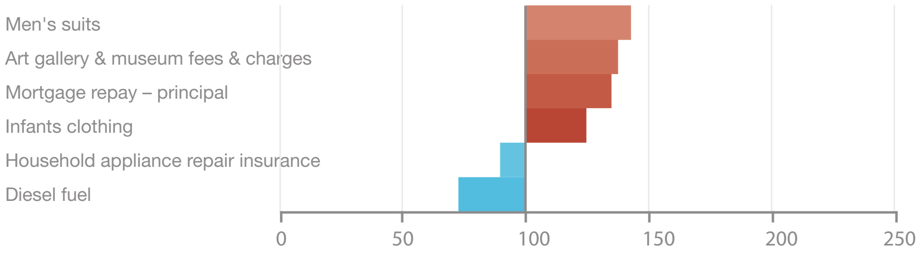

Population Profile

93% of the 382,697 people that live in this segment live in Major Cities.

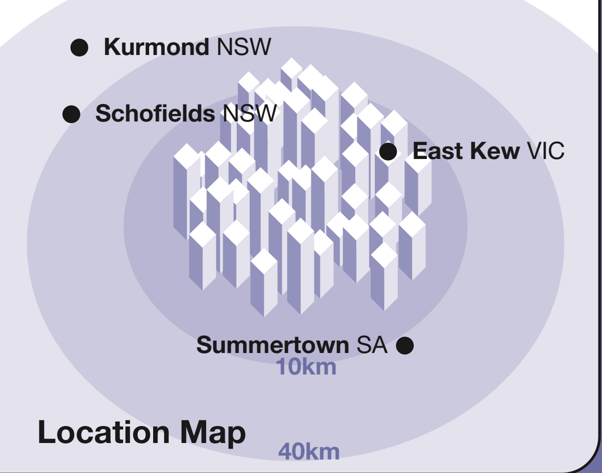

Location Map

Pathfinder Solutions © Copyright 2003 /// phone: +61-3-9670 3030 /// email: info@pathfindersolutions.com.au

PATHFINDER SEGMENTS OF AUSTRALIA

12 Mature Families

Teenage turf

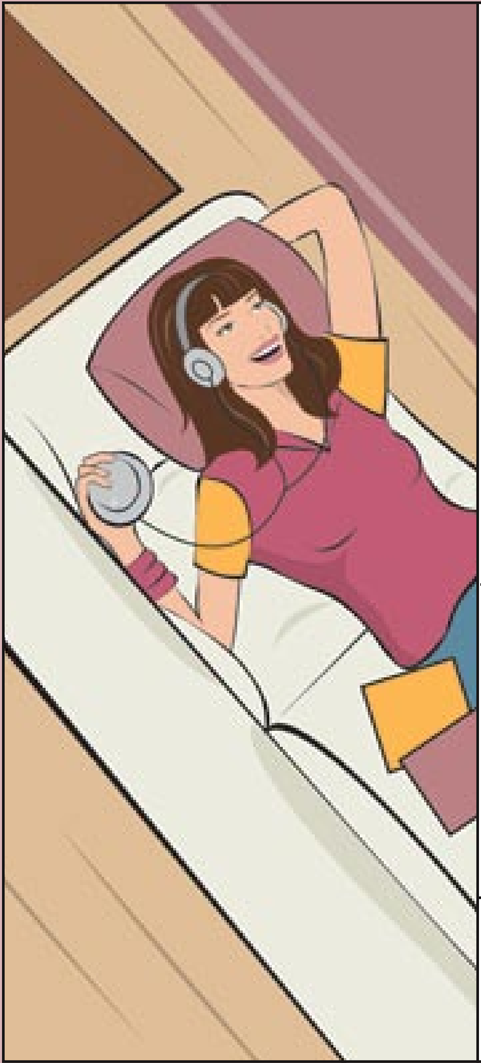

Segment Description

Here we're talking parents with grown up kids – either dependent or non-dependent - still living at home. Their age groups are as you'd expect – 45-54 years old and teenagers.

Typically, there are four to five people per household, two or three of which are probably teens listening to music their parents don't get.

Many homes are owned outright and were probably extended in the late eighties to mid nineties. Households with incomes above $75,000 per year are over represented (1.4 times as likely).

Three cars per household is common – one probably parked on the nature strip. Also common are pets, pergolas, garages, patios and swimming pools. A reasonable amount is also spent on vehicle servicing, repair and maintenance of household durables.

Defining Features

1. Teenagers and 45-54 y.o. are over represented in this segment
2. Many homes are owned outright with typical mortgage repayments around $1,200-$1,400 per month
3. Home extensions were common from the late 80s to mid 90s in this segment
4. There are average levels of o/s born people in this segment
5. Family income is over represented in $75,000+ bands and under represented in bands below $35,000 p.a.

Key Expenditure

Average Australian Household Index = 100

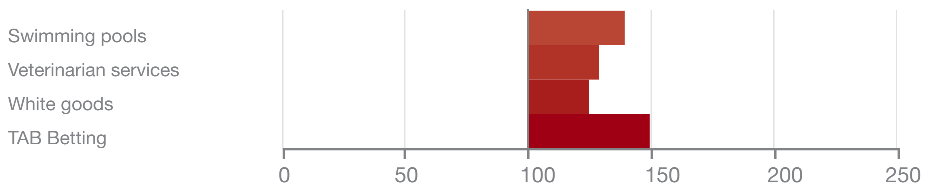

Population Profile

99% of the 251,834 people that live in this segment live in Major Cities.

Location Map

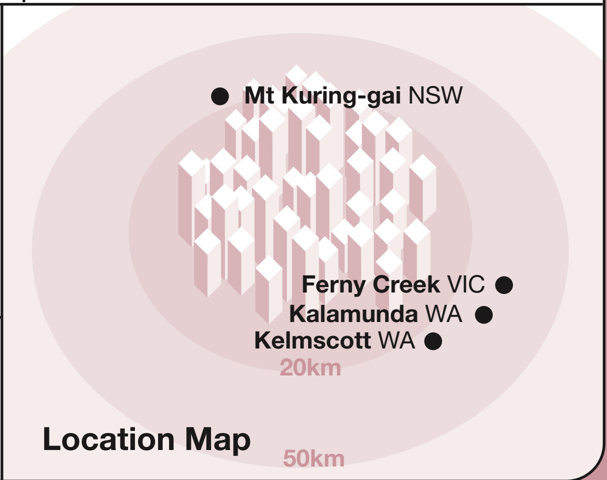

Pathfinder Solutions © Copyright 2003 /// phone: +61-3-9670 3030 /// email: info@pathfindersolutions.com.au

PATHFINDER
SEGMENTS
OF AUSTRALIA

13 Fringe Lifestyle

Away from the Big Smoke

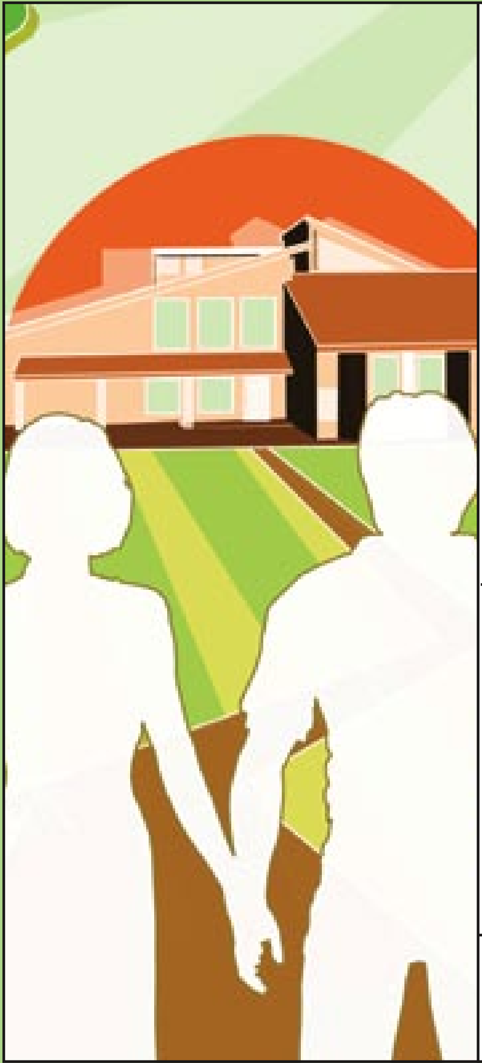

Segment Description

On the outskirts of major cities (and some inner regional), can be found a practically-minded bunch who like a bit more land for their dollar (average around eight hectares!), and space between themselves and the neighbours.

They're likely to be builders, engineers or teachers – good, practical careers. The 50-54 age group are over represented and they're likely to be migrants from the U.K, Netherlands and Germany. Mind you, they're hardly newcomers having been here for more than 20 years.

In terms of income, they are 1.2 times as likely to have household incomes above $60,000 per year. They're on the outskirts, so public transport is a rarity; consequently they drive trucks and cars to get to work with 2-3 cars per household being the norm. Their house might be new or built in the late 80s when there was a surge in new homes in these areas. But these areas have also seen some development activity in the last 2-3 years.

Wood-heating is common and their money is practically spent on coats and cardigans, ambulance insurance, LPG, camping gear and fuel. They're more likely to send their kids to public schools. If they do lash out it's probably on a swimming pool or home improvements.

Defining Features

1. This segment is typically found in larger allotments on the outskirts of major cities
2. Ages 50-54 are over represented, and migrants from the UK, Netherlands and Germany are common
3. This segment has 1.25 times the average level of household income above $62,400 p.a.
4. There has been substantial amounts of development occurring in this segment over the last 2-3years.

Key Expenditure

Average Australian Household Index = 100

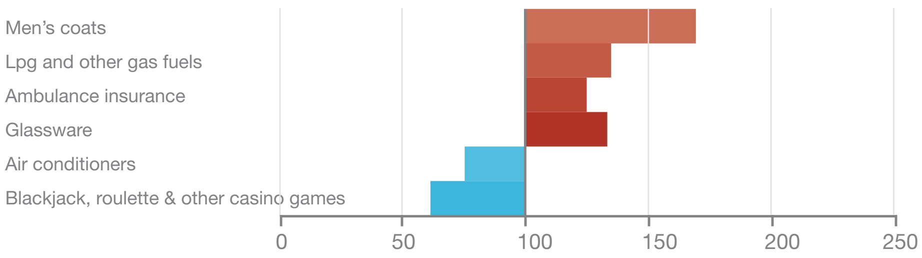

Population Profile

70% of the 281,746 people that live in this segment live in Major Cities, 27% live in Inner Regional areas and 3% in Outer Regional.

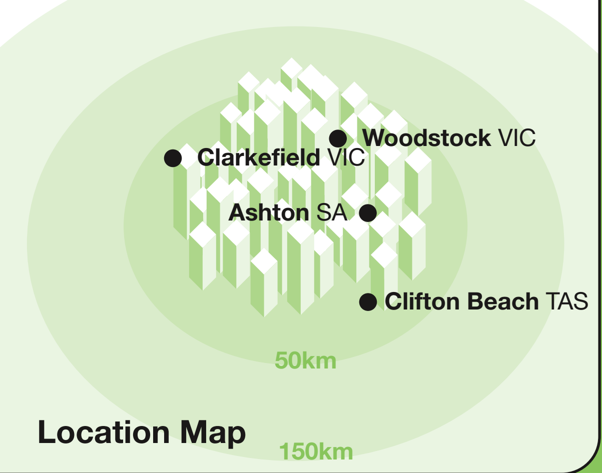

Location Map

Pathfinder Solutions © Copyright 2003 /// phone: +61-3-9670 3030 /// email: info@pathfindersolutions.com.au

PATHFINDER SEGMENTS OF AUSTRALIA

14 Lifestyle Allotments

Rancho relaxo

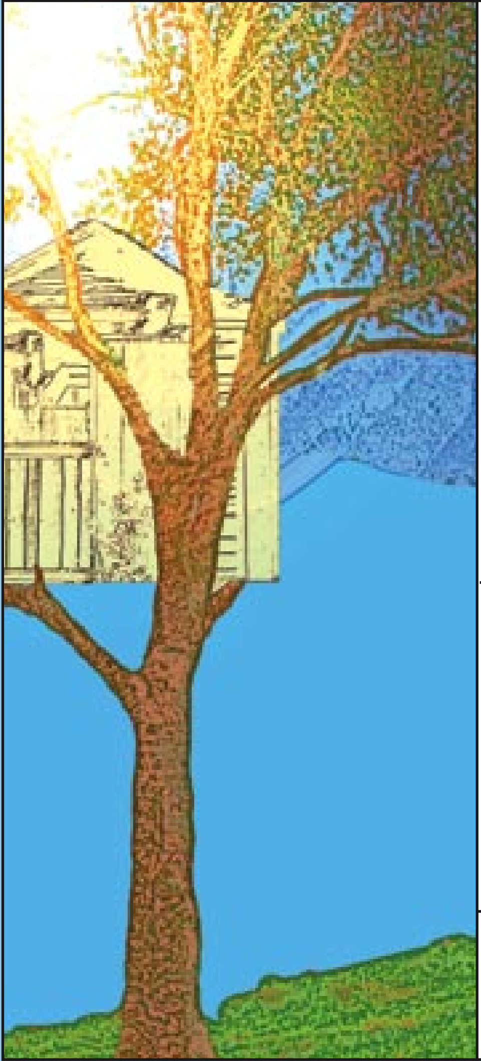

Segment Description

These guys are far enough out of the city to be in a regional area but close enough that they are not far! These are city/country folk, the small-time farmers. These areas have experienced a flurry of building activity since 2000, both commercial and residential, including extensions and swimming pools.

The age groups well represented are as you might expect 50-54 and 0-14. Many are from North-West Europe and send their kids to non Catholic private schools for primary and secondary education (above 50%).

You are 1.2 times as likely to find households earning more than $60,000 per year, with mortgages in the $1,200 to $2,000 per month bracket also over represented. They mainly work in construction and agriculture, but also in trades, clerical and other services.

This segment contains big families, with five and six person households accounting for 16%. They ride horses, read Australian Geographic and spend time and extra money on improving their estates. They also take the odd vitamin (usage is 50% above average) and spend money on kitchen and other furniture, glass ware, child care, lawnmowers, boats, pets, and insulation.

Defining Features

1. Over 25% of families in this segment have more than 4 people living at home
2. 12.5% of households have 3 or more motor vehicles and this segment is well under represented in travelling to work by public transport
3. This segment is 1.7 times as likely to work from home
4. Monthly loan repayments of over $1,000 are over represented in this group

Key Expenditure

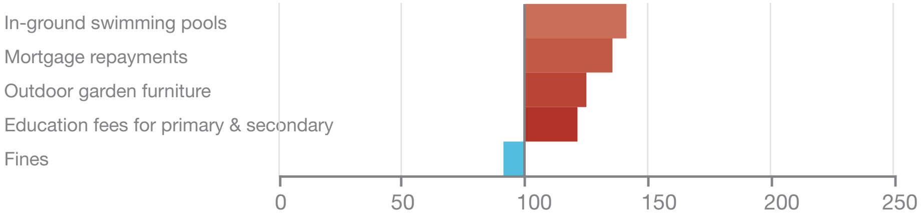

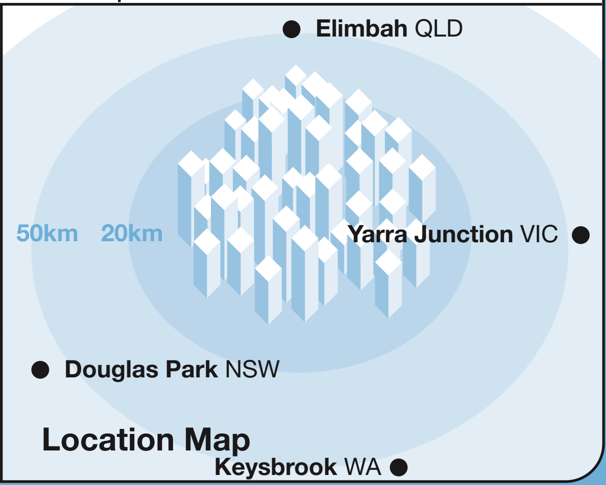

Location Map

Population Profile

52% of the 163,271 people that live in this segment live in Major Cities, 44% live in Inner Regional areas and 3% in Outer Regional.

Pathfinder Solutions © Copyright 2003 /// phone: +61-3-9670 3030 /// email: info@pathfindersolutions.com.au

PATHFINDER SEGMENTS OF AUSTRALIA

15 Prosperous Trades

Salt of the Earth

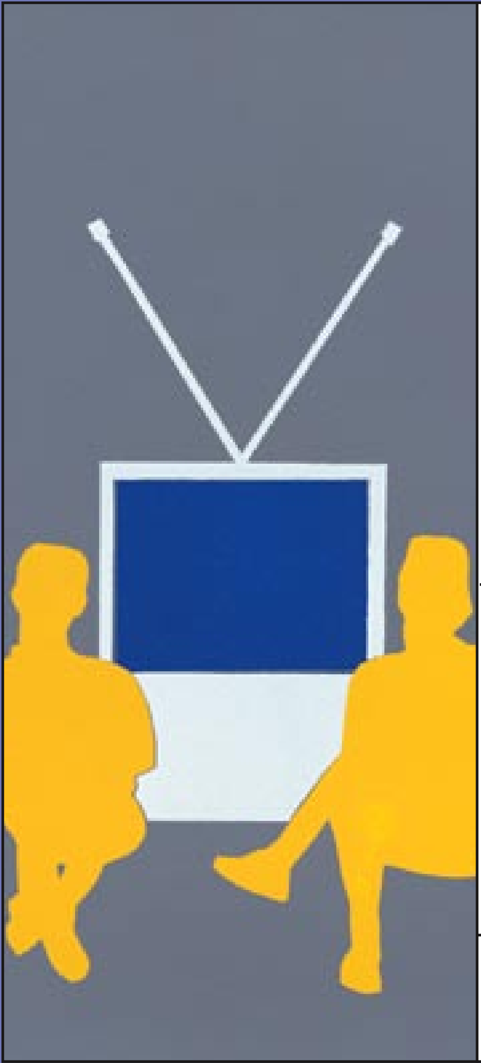

Segment Description

These are the manufacturing, construction and communications services people who work hard, earn a pretty buck, and then go home to pay TV.

Yearly household income is very likely to be in the range of $60,000 to $100,000. Interestingly, there are very few low income couples with children or couples without children. Many are couples with dependent and non-dependent kids.

This major city segment is interesting, in that while many use their cars to get to work, there is also high expenditure on public transport. In fact, those that rely on public transport, use 2 or more modes.

They are probably (10% more likely to be) migrants from Lebanon, Croatia, Egypt, Fiji, Sri Lanka or the Philippines who've arrived in the eighties.

Less likely to be renting, many fully own or have a mortgage on their home.

They often tuck into seafood and spend money on men's coats, vegetables, rail fares, blinds and footwear.

Defining Features

1. Household incomes are over represented in the upper bands
2. Households are 30% more likely to have non-dependant children
3. All age-groups over 65 are under-represented
4. Separate houses (and flats attached to houses) account for over 90% of homes
5. They are more likely than average to have been at the same address 5 years ago

Key Expenditure

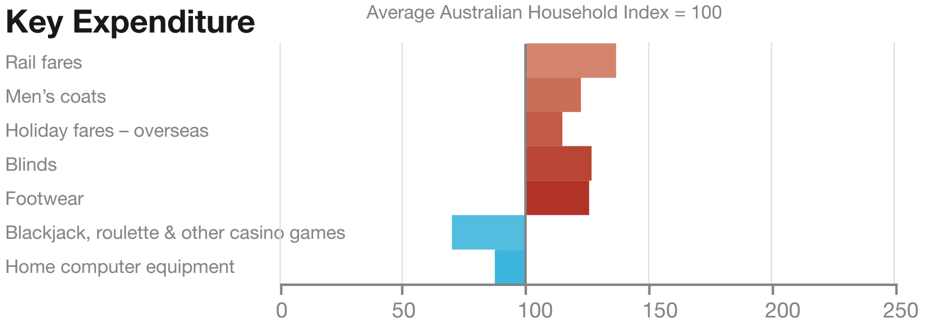

Population Profile

97% of the 319,194 people that live in this area segment live in Major Cities.

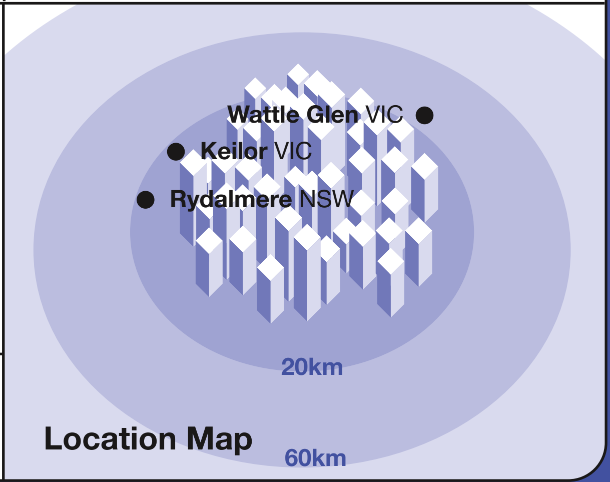

Location Map

Pathfinder Solutions © Copyright 2003 /// phone: +61-3-9670 3030 /// email: info@pathfindersolutions.com.au

PATHFINDER SEGMENTS OF AUSTRALIA

16 Dual Occupancy Hopefuls

Backyards for sale

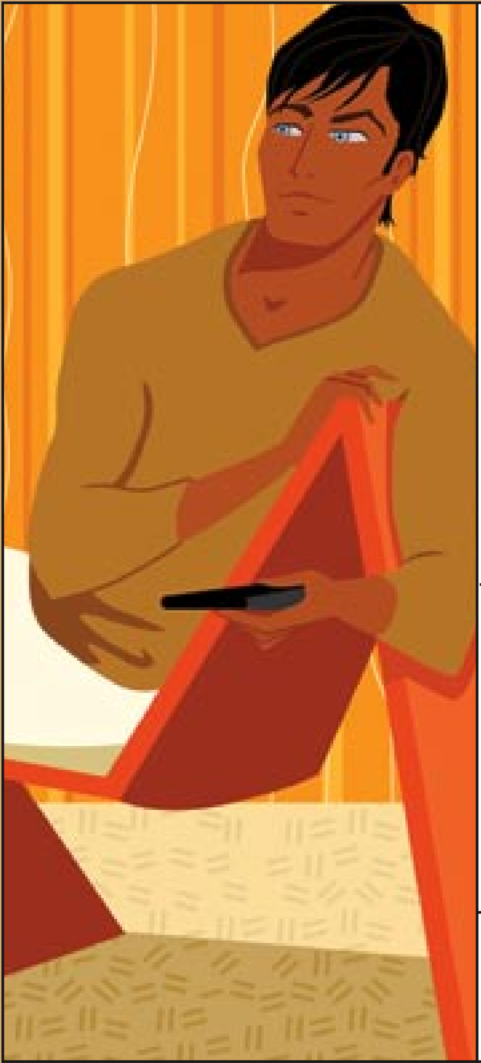

Segment Description

This is a culturally diverse segment with over a quarter of the occupants having been born in countries such as China, Hong Kong, Malaysia, Korea, Macedonia and Greece.

They are happy to sell off their backyard. In fact, they have the highest level of building approvals for dual occupancy developments and permits for construction of flats over the past ten years – two to three times the national rate.

A third are lone person households, but group households, older one parent families as well as couples without kids are also likely to be found.

There's a strong young 25-34 year old age group who work mainly in areas such as finance, insurance, cultural and recreational services. While there is also a predominance of the older 65-79 year old age group.

Household incomes are average across all bands, and if they are renting they are likely to be paying around $200-$299 per week in rent.

These guys aren't rich but get by on an average household income.

Defining Features

1. This segment has 2 to 3 times the number of building approvals for dual occupancy and flats over the last 10 years compared to the national average
2. 30% of households are lone households
3. More than 3/4 of the people in this segment were born overseas
4. Household incomes are average across all bands in this segment

Key Expenditure

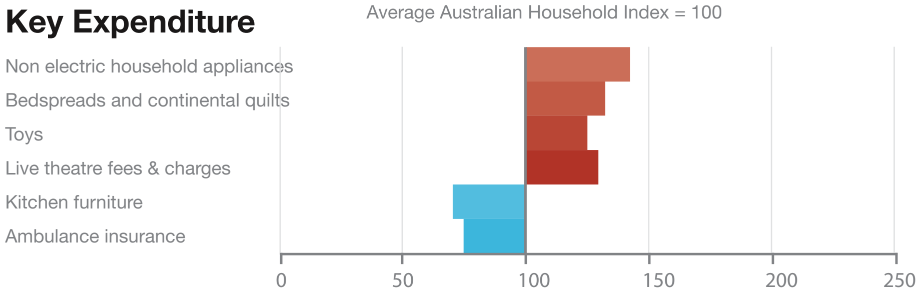

Population Profile

93% of the 325,136 people that live in this segment live in Major Cities.

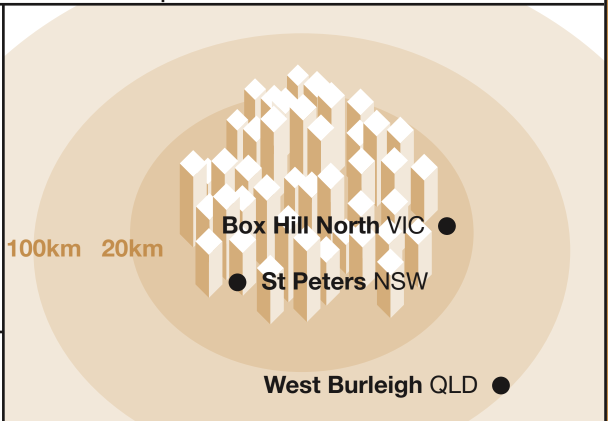

Location Map

Pathfinder Solutions © Copyright 2003 /// phone: +61-3-9670 3030 /// email: info@pathfindersolutions.com.au

PATHFINDER SEGMENTS OF AUSTRALIA

17 Established Greek Communities

It's all Greek to them!

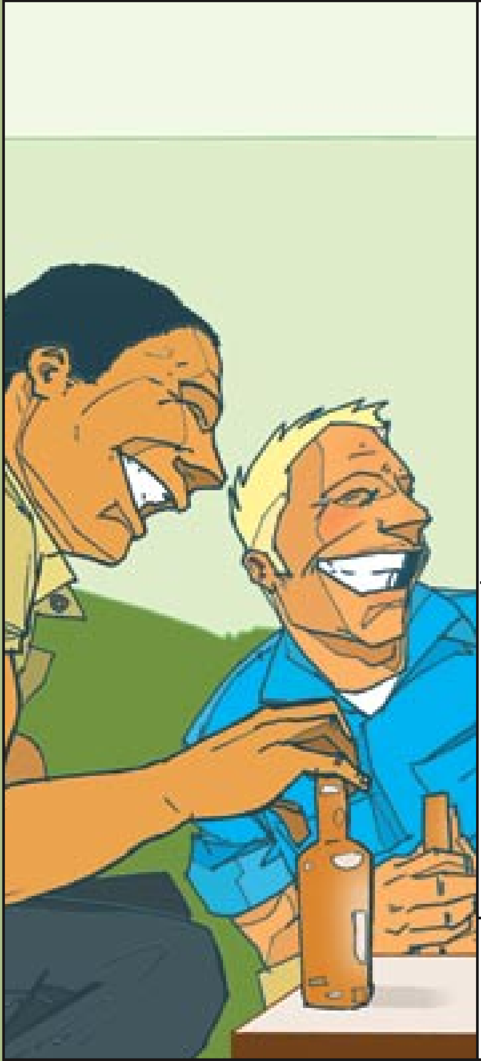

Segment Description

This major Greek city segment keeps true to its roots, with over a third speaking the language at home and over 20% being of Greek Orthodox faith.

Around two thirds of this segment has been in Australia for over 15 years and there are large groups of young 25-34 year olds and over 55s.

They don't like to live alone and you are 1.2 times as likely to find households that earn over $100,000 per year in this segment.

It's likely that they own their home outright (over half in fact) and they have made renovations (many were done in the early 80s).

This group love soccer, Optus Pay TV, Sunday newspapers and cook books, while use of the Commonwealth Bank is high.

They spend their 'drachmas' on a diverse basket of consumables including pasta, rice, seafood and fish, fresh fruit and vegetables, cardigans and jumpers, dry cleaning, medicines and pharmaceutical products. In the more durable range, they also spend on lounge and dining furniture, stoves, air conditioners and dishwashers.

Defining Features

1. 45% of people in this segment speak a language other than English at home, 1/3 of all people speak Greek
2. 20% of people in this segment are Greek Orthodox
3. 2/3 of migrants have been in Australia for over 15 years
4. Lone person households are unlikely in this segment & approximately 10% of households earn $104,000 p.a.

Key Expenditure

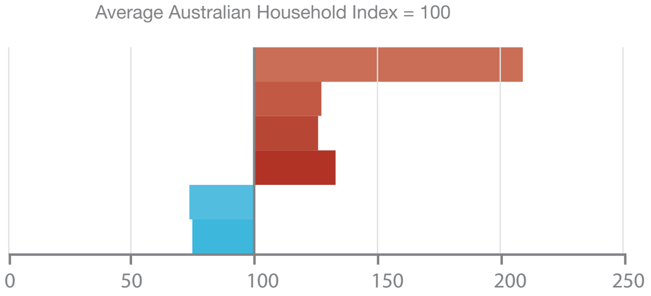

Population Profile

100% of the 230,134 people that live in this segment live in Major Cities.

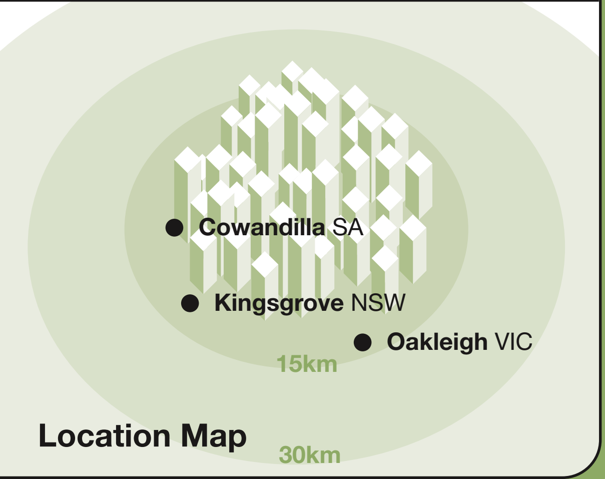

Location Map

Pathfinder Solutions © Copyright 2003 /// phone: +61-3-9670 3030 /// email: info@pathfindersolutions.com.au

PATHFINDER SEGMENTS OF AUSTRALIA

18 Struggling City Fringe

On the outer

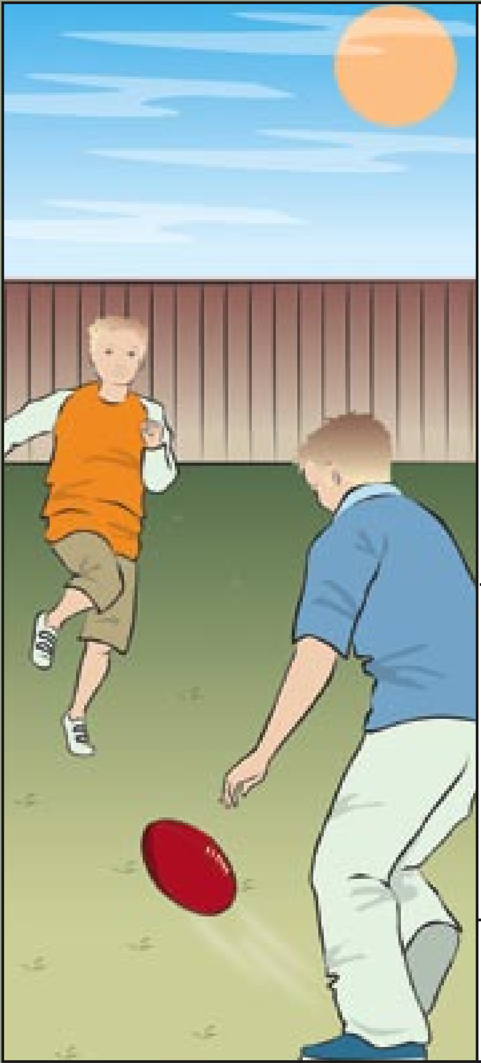

Segment Description

Around the major cities and inner regional areas are those who earn less and are more likely to work in trades.

This segment is over represented in the $60,000 to $75,000 per year household income band, but since 1991 there's been a growth in lower income groups and a decrease in higher groups (households in the second lowest income quartile increased 20% and the proportion in the top quartile decreased by 27%).

Of note is the fact that lone person households have increased by nearly 50% in this segment over the same period.

There are a number of young families with mortgages, the parents at around 45-54 years of age with kids around 10-19. The kids are likely to drop out of school at Year 10 or 11 and go to TAFE to study part time.

Homes are being purchased with the average mortgage payment in the $800 - $900 per month range.

Spend is high on ambulance insurance, men's coats and pullovers and LPG.

Defining Features

1. This segment has 1.3 times the average level of household income in the $60,000 to $70,000 p.a. bracket
2. Houses in the top quartile have declined by 27%
3. Since 1991 the % of households in the second lowest quartile has increased by 20%
4. Average mortgage payments are in the $800 - $900 per month range
5. There is a mixture of lone person and family households

Key Expenditure

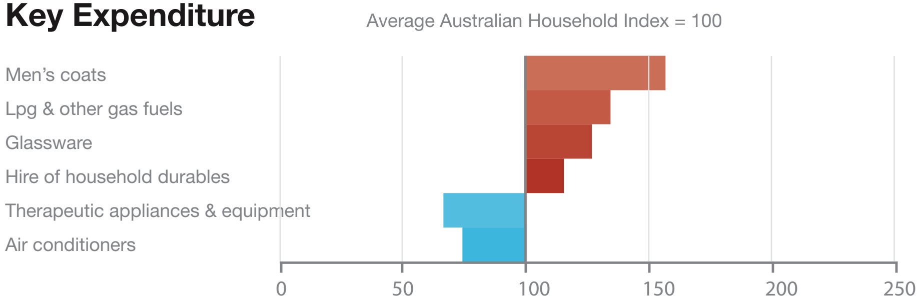

Population Profile

87% of the 454,966 people that live in this segment live in Major Cities and 12% live in Inner Regional areas.

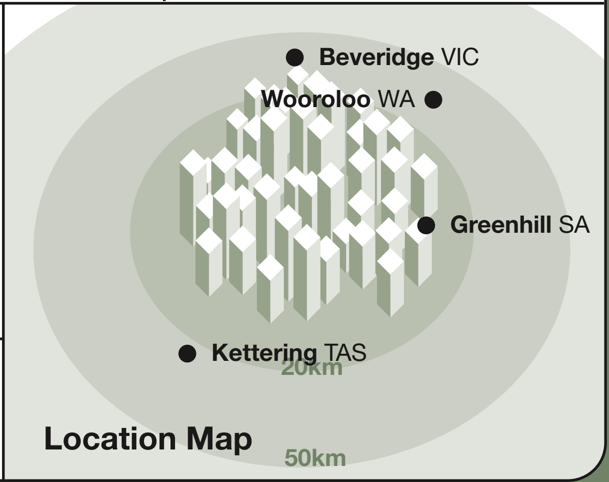

Location Map

Pathfinder Solutions © Copyright 2003 /// phone: +61-3-9670 3030 /// email: info@pathfindersolutions.com.au

PATHFINDER SEGMENTS OF AUSTRALIA

19 Middle Income Suburban Families

Meet the Joneses

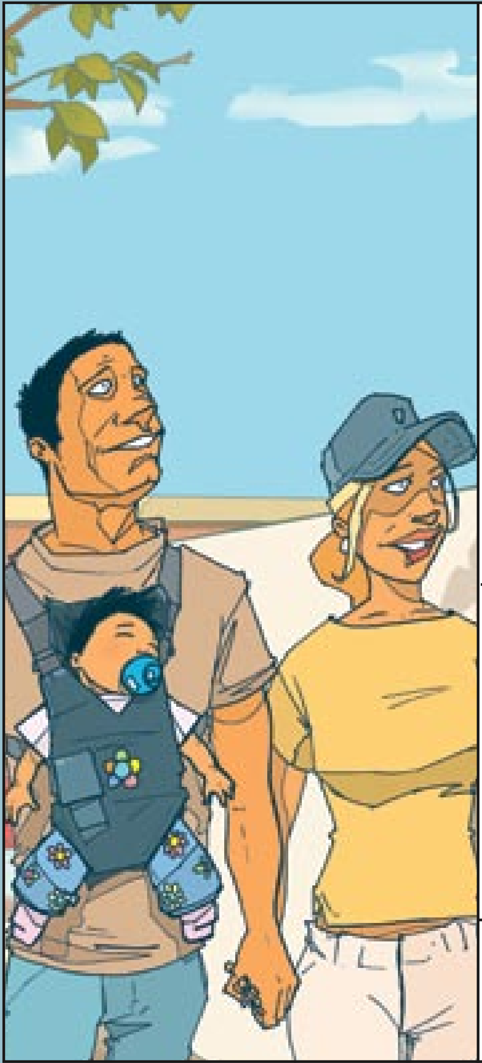

Segment Description

This is your average suburban family area.

Household income levels are average across all bands with the exception of $60,000-$75,000 which is over represented, and they tend to work in the public sector, health and education. People from all age groups live here (although the over 80s are under represented).

A great proportion is studying full or part-time at TAFE or University (25% above average), and people are likely to use the Internet at home.

It's a cosy group who are likely to own their homes or have a mortgage. 55% have lived at the same address for five or more years, and they're likely to spend money on swimming pools and extensions (construction of such additions peaked from 1988-1995) or carports and garages.

To complete the set these folk are likely to have pets with around 20% having three or more dogs!

Spending is high on whitegoods, kitchen furniture, repair and maintenance of household durables, child care fees, insulation, musical instruments, pets and jewellery.

Defining Features

1. 55% of people have lived at the same address for five years or more
2. Employment in the public sector, health and education are strong
3. This segment has average income levels across most income bands
4. While there is no dominant ethnic group - 30% of this segment is born overseas (in line with the Australian average)

Key Expenditure

Average Australian Household Index = 100

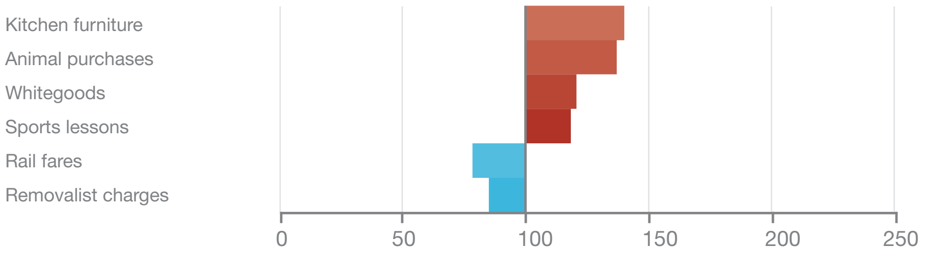

Population Profile

86% of the 236,046 people that live in this segment live in Major Cities, 10% live in Inner Regional areas and 4% in Outer Regional.

Location Map

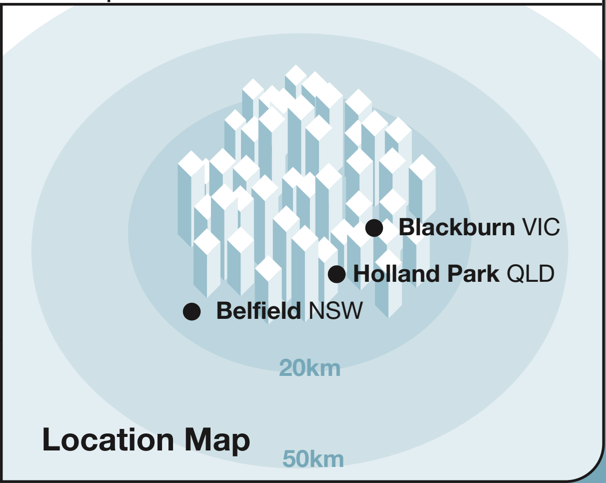

Pathfinder Solutions © Copyright 2003 /// phone: +61-3-9670 3030 /// email: info@pathfindersolutions.com.au

PATHFINDER SEGMENTS OF AUSTRALIA

20 Student Enclaves

Turn it down!!

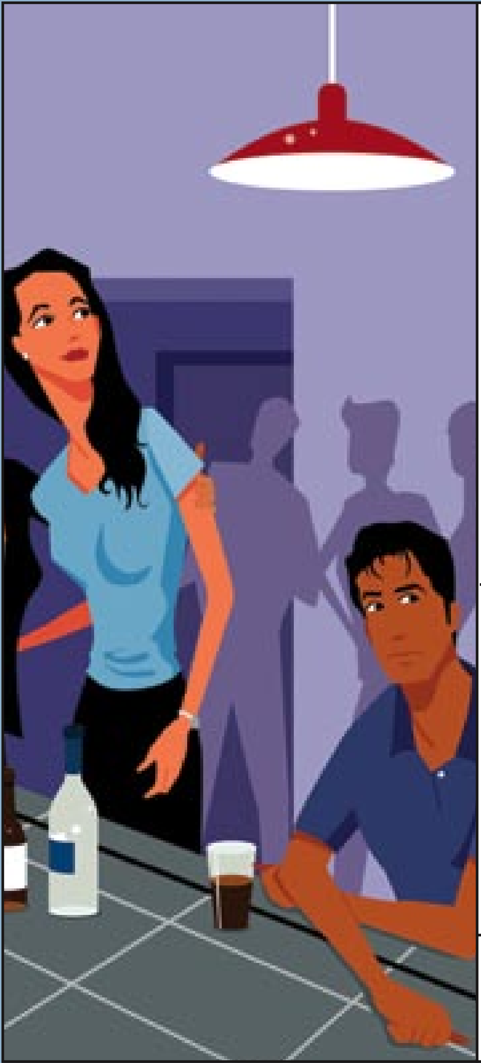

Segment Description

This bunch is nearly four times as likely to earn no income.

That's probably because around a third are studying full time at University. If they're earning money at all, it's likely to be from working part-time in cafes, restaurants or in education.

Yearly incomes below $10,000 are over represented, as is shared accommodation (and probably loud music!). More than 20% live in halls of residence or other student housing. Most rent their accommodation, with the weekly rent being around $200 to $400.

Around 30% were born overseas, with the proportion of Chinese speaking students in these areas increasing by almost 50% between 1991 and 2001.

Since 2000 there has been a significant increase in commercial development and the building of flats within these areas.

Interests of this segment are typically volleyball, roller blading, skating, scuba diving and working out in the gym, as well holidays in Asia.

Defining Features

1. 30% of people in this segment were born overseas
2. More than 20% live in student housing provided by universities and other tertiary institutions
3. Part time employment is high in cafes, restaurant, hotels and education is common
4. Twice as many homes are flats/apartments, compared with the Australian average
5. Most people use the Internet

Key Expenditure

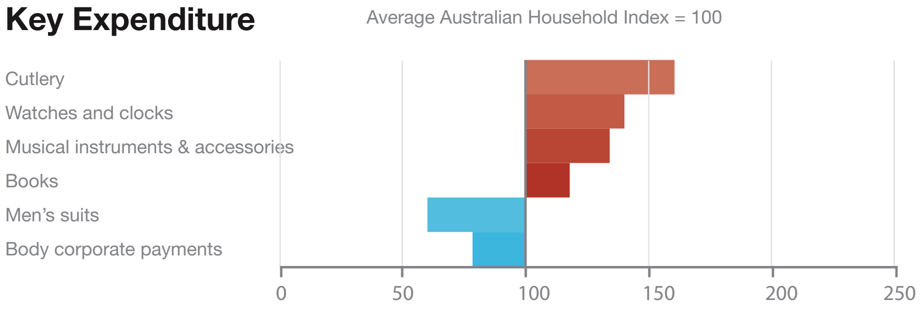

Population Profile

64% of the 96,905 people that live in this segment live in Major Cities, 31% live in Inner Regional areas and 5% in Outer Regional.

Location Map

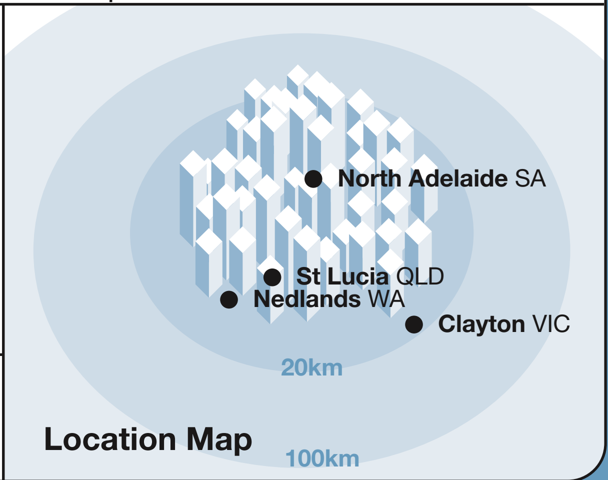

Pathfinder Solutions © Copyright 2003 /// phone: +61-3-9670 3030 /// email: info@pathfindersolutions.com.au

PATHFINDER SEGMENTS OF AUSTRALIA

21 New Housing Estates

Welcome to Pleasantville

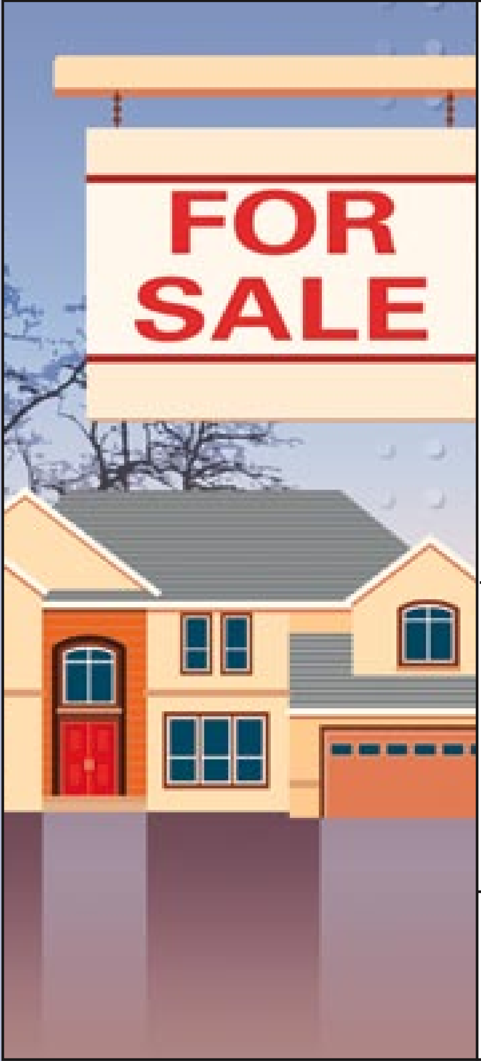

Segment Description

Here we find the new housing developments targeted at young, slightly above average income families.

This major city and regional segment has become more and more fashionable. From 1991 to 2001 it had a growth spurt of 22% in people from the second top income quartile and a decrease of 18% of those in the lowest income quartile. You are 1.3 times as likely to find household income levels in the $60,000 to $75,000 per year bracket.

Inhabitants are likely to be aged 30-39 and have pre-school aged kids. And there are lots of them around, with a quarter of people in the segment being under 14. Four to five person households are common.

They're paying off mortgages with monthly repayments being around $800-$1600. Their homes are new, and only around a third has lived there for over five years.

They've pretty much all got jobs – unemployment is low and cars are the favoured transport option with public transport getting the thumbs down.

Spending is high on vehicle expenses, kitchen furniture and ambulance insurance.

Defining Features

1. Only 1/3 of people have been in their home for more than 5 years
2. 1 in 5 households have moved within the last year
3. Ages 30- 39 are over represented in this segment
4. The proportion of children in pre-schools is high
5. Unemployment levels in this segment are low
6. Over 2 million people reside in this segment

Key Expenditure

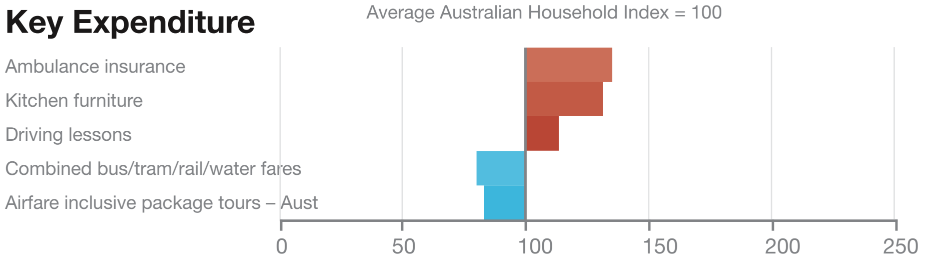

Population Profile

63% of the 2,092,041 people that live in this segment live in Major Cities, 25% live in Inner Regional areas, and 10% live in Outer Regional areas.

Location Map

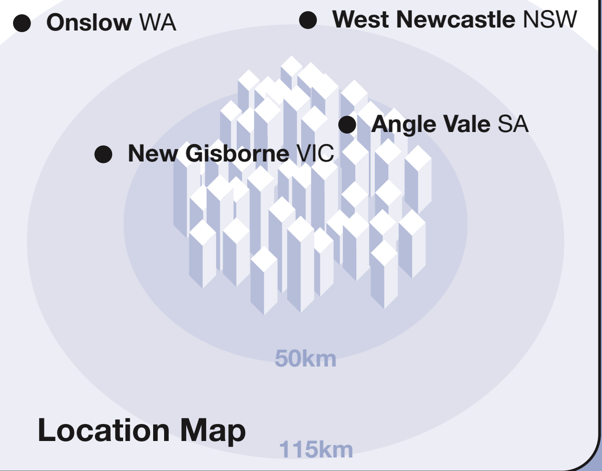

Pathfinder Solutions © Copyright 2003 /// phone: +61-3-9670 3030 /// email: info@pathfindersolutions.com.au

PATHFINDER SEGMENTS OF AUSTRALIA

22 Maturing Housing Estates

Earlier date housing estates

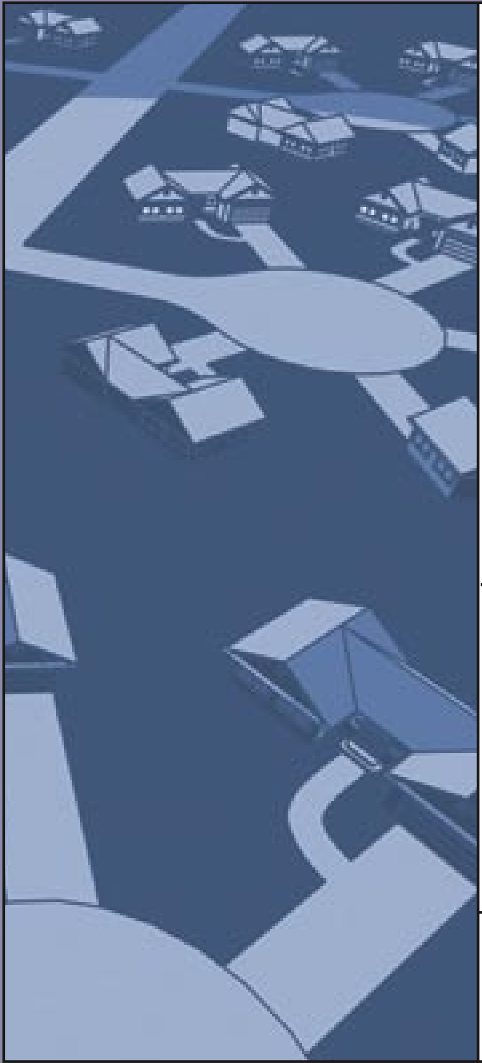

Segment Description

These are the housing estates which no longer smell of fresh paint.

People in this major city and regional segment are making changes to their properties - installing pools at two to three times the average, installing garages, verandas and pergolas (peaking in 1993). Around half (47%) have lived in their homes for five or more years.

These folk are less bright-eyed than those in the newer housing estates and are 10% more likely to have a split partnership. Their kids are still young with 26% being 14 and under.

They're likely to have two cars, a mortgage with repayments being between $600 and $800 per month and do not use public transport, preferring their own wheels.

You are 1.3 times as likely to find household income levels in the $60,000 to $75,000 per year bracket.

Spending is high on kitchen furniture, non-electrical household appliances and AV equipment.

Defining Features

1. Half of the people have been in their home for more than 5 years
2. 1 in 6 households have moved within the last year
3. A quarter of the population is aged under 14 years old
4. There are no dominant ethnicities in this segment, and over 70% of residents were born in Australia
5. The $62,000 – $78,000 p.a. income range is over represented.

Key Expenditure

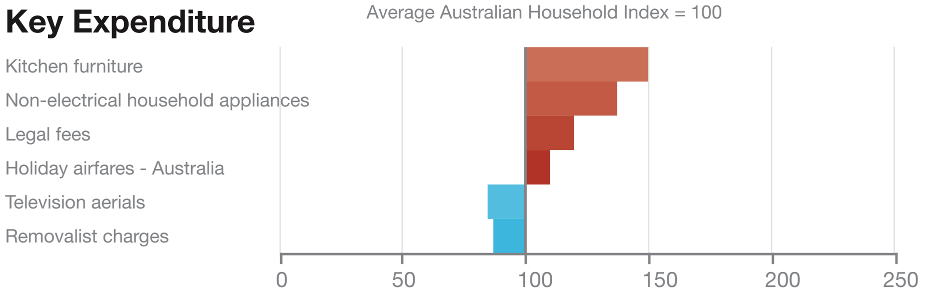

Population Profile

75% of the 694,050 people that live in this segment live in Major Cities, and 18% live in Inner Regional areas.

Location Map

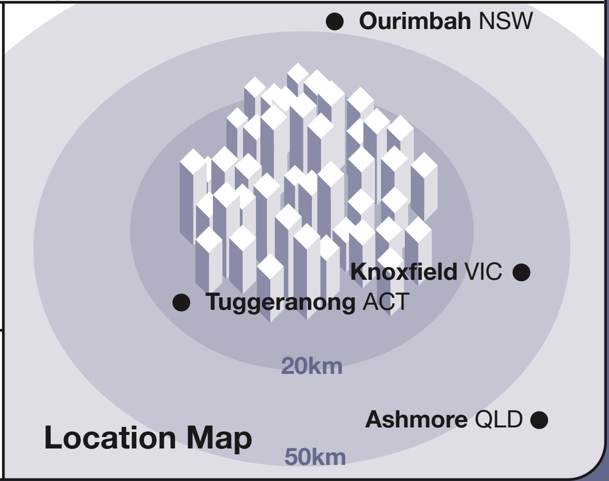

Pathfinder Solutions © Copyright 2003 /// phone: +61-3-9670 3030 /// email: info@pathfindersolutions.com.au

PATHFINDER SEGMENTS OF AUSTRALIA

23 Aging Suburban Areas

Cuppa and a sticky bun

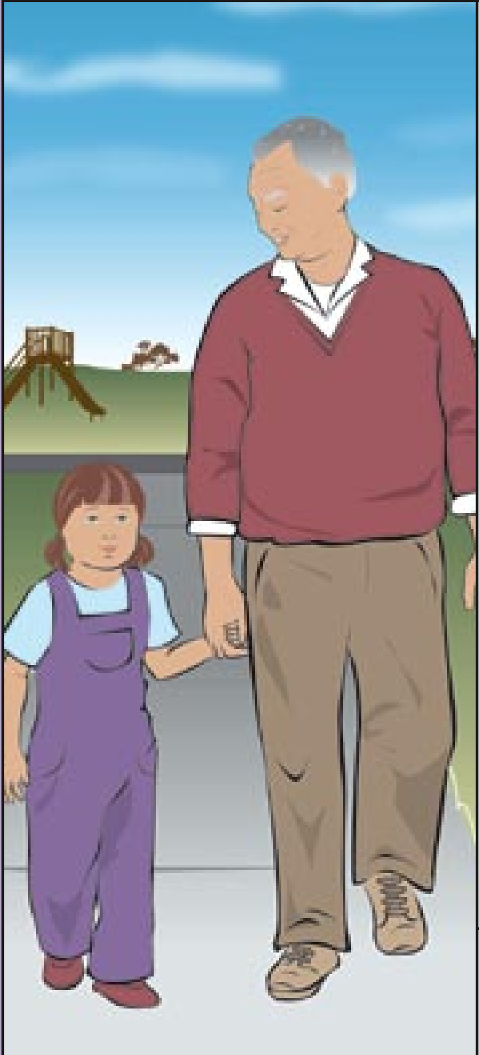

Segment Description

A quarter of the people in this predominantly major city segment are over 65.

They mainly live in homes built in the 1940s and 50s however since 1988 there has also been a significant rise in the development of flats and dual occupancy homes in these areas. Currently, around 20% are flats or townhouses.

There are lots of people living alone (over 30%) and widowers make up 12% of the population. Around 5% live in non-private residences.

They earn an average amount in general; however those earning $10,000-$15,000 per year are over represented, probably due to the pension.

Most spend time watching their grandkids, Optus and Football.

They spend their money (probably hidden in the teapot) on men’s cardigans and pullovers, table linen, public transport fares and repairs of household goods.

Defining Features

1. This segment has double the proportion of people aged over 65
2. Around 3/4 of primary and secondary school children attend Catholic schools
3. Monthly loan repayments are close to average in all bands except for the lowest band which is 37% over represented
4. Many of the original homes were built in the 1940's and 1950's

Key Expenditure

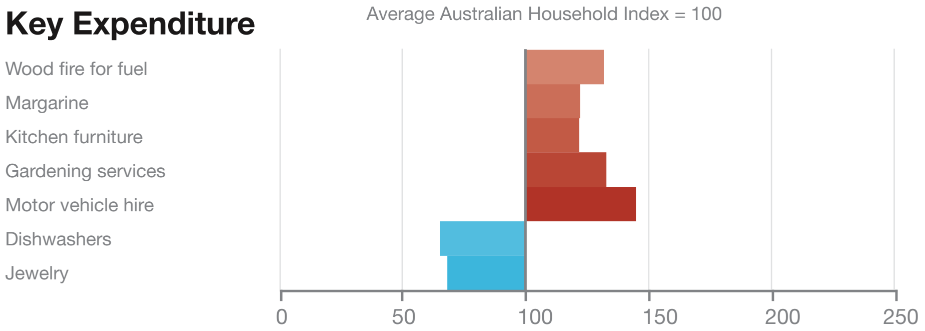

Population Profile

92% of the 331,023 people that live in this segment live in Major Cities, and 8% live in Inner Regional areas.

Location Map

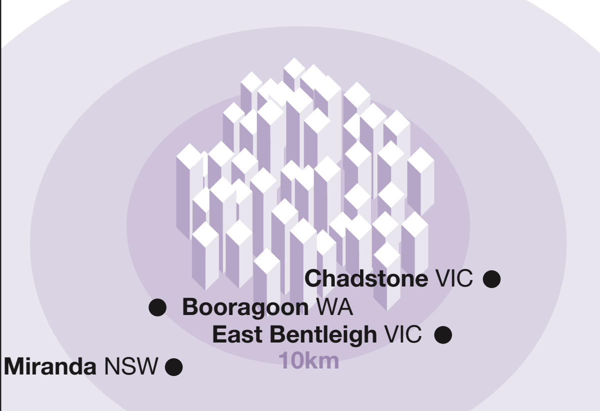

Pathfinder Solutions © Copyright 2003 /// phone: +61-3-9670 3030 /// email: info@pathfindersolutions.com.au

PATHFINDER SEGMENTS OF AUSTRALIA

24 Established Italian Migrants

The mamas and the papas

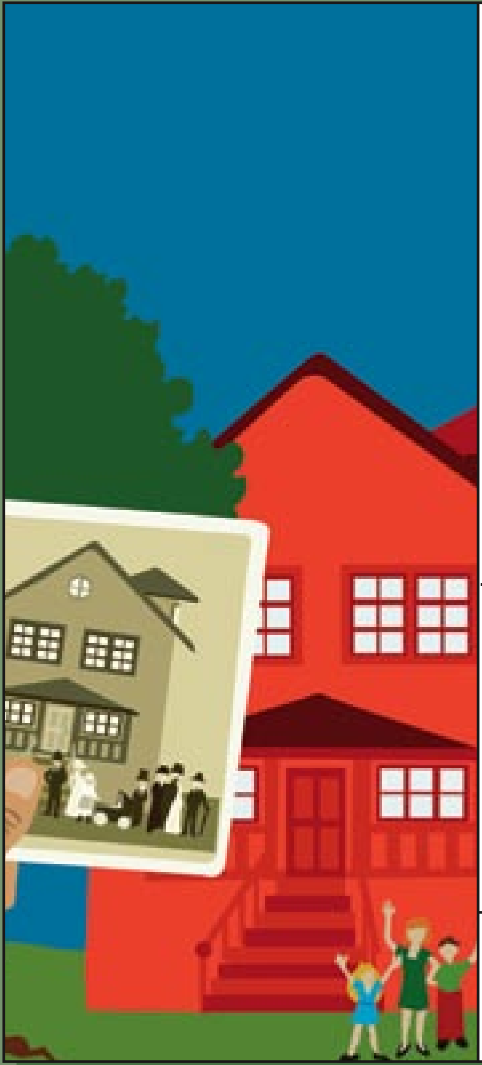

Segment Description

The Italian-born population is prevalent in this mainly major city segment and many have been here since the 50s.

There are very few heathens amongst them with the highest proportion of Christians and over 40% being Catholic.

Many original migrants had little education themselves and conscientiously send their kids to Catholic primary and secondary schools.

Household income levels are average across all bands, a great proportion being employed in manufacturing. They spend money on enhancing their properties and cars (often three or more cars per household, with Alpha's over represented!). Over half own their homes outright.

There are many 65-74 year olds in this group, as well as families with non-dependent kids.

They also spend their money on fish and seafood, mains gas, men's cardigans and pullovers, table and kitchen linen, LPG, vehicle insurance, overseas holidays, travel insurance and school fees.

Defining Features

1. 1 in 5 residents of this segment speak Italian at home
2. 40% are Catholic
3. Couple families with non dependent children are over represented
4. Many homes have 3 or more cars
5. More than half of the households own their homes outright

Key Expenditure

Average Australian Household Index = 100

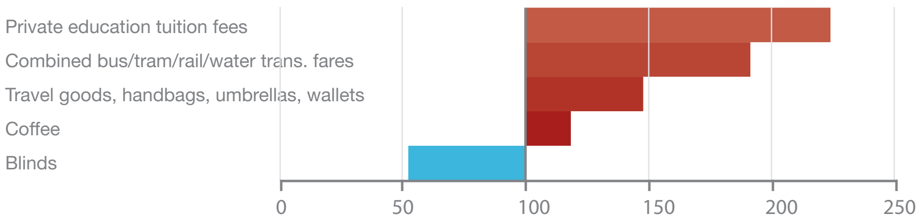

Population Profile

94% of the 381,433 people that live in this segment live in Major Cities.

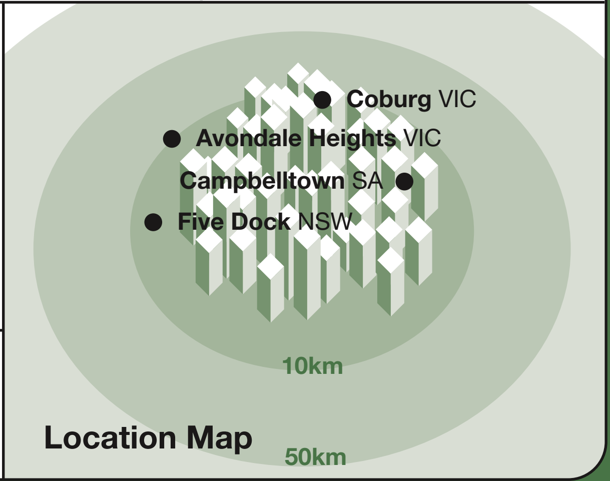

Location Map

Pathfinder Solutions © Copyright 2003 /// phone: +61-3-9670 3030 /// email: info@pathfindersolutions.com.au

PATHFINDER SEGMENTS OF AUSTRALIA

25 Settled Trades & Manufacturing

Toolkit territory

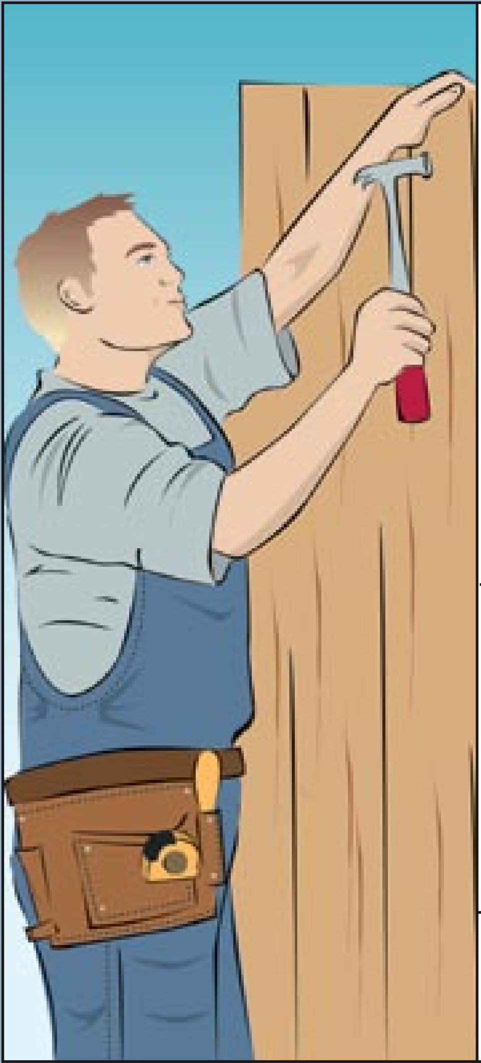

Segment Description

This segment is losing its richer inhabitants and gaining those with lower incomes.

Many of the people living here are employed in manufacturing, trades and other intermediate jobs: One in four males work as a tradesman or in a related job, and one in three women work in a clerical or sales and service role, or in a physical labouring job. All management, professional or associate professional roles are under-represented. There are lots of migrants from South Eastern Europe or South East Asia and around 18% speak a language other than English at home.

There's a declining relative income and where home improvements were high in the late 80s and early 90s, over the past five years there's been a decline.

Households are 1.3 times as likely to be earning between $60,000 and $75,000 per year.

Mortgages are around one in three.

Defining Features

1. 16% of the workforce is employed in manufacturing and trades
2. 18% of people speak a language other than English at home
3. Over the last 10 years households in the top income quartile have declined by 27% and incomes in the second lowest quartile have increased by 17%
4. South Eastern Europe and South East Asian birthplaces are common
5. 60% of people live at the same address as 5 years ago

Key Expenditure

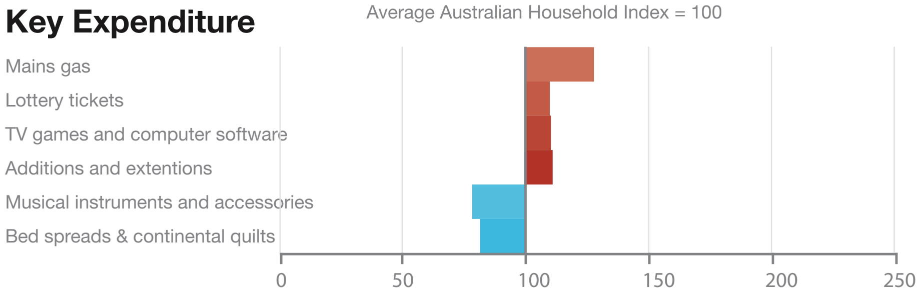

Population Profile

96% of the 670,117 people that live in this segment live in Major Cities.

Location Map

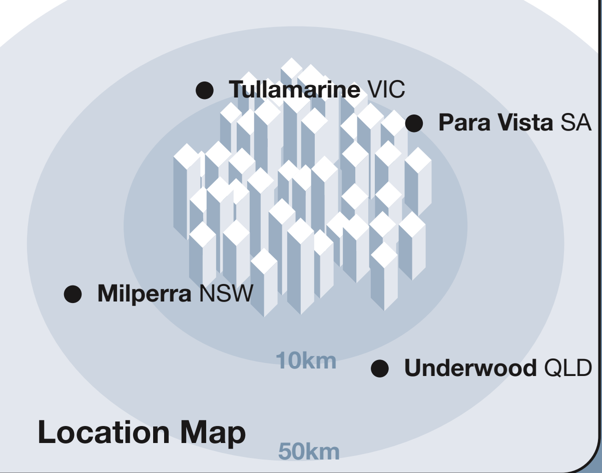

Pathfinder Solutions © Copyright 2003 /// phone: +61-3-9670 3030 /// email: info@pathfindersolutions.com.au

PATHFINDER SEGMENTS OF AUSTRALIA

26 Moving Down

From riches to rags

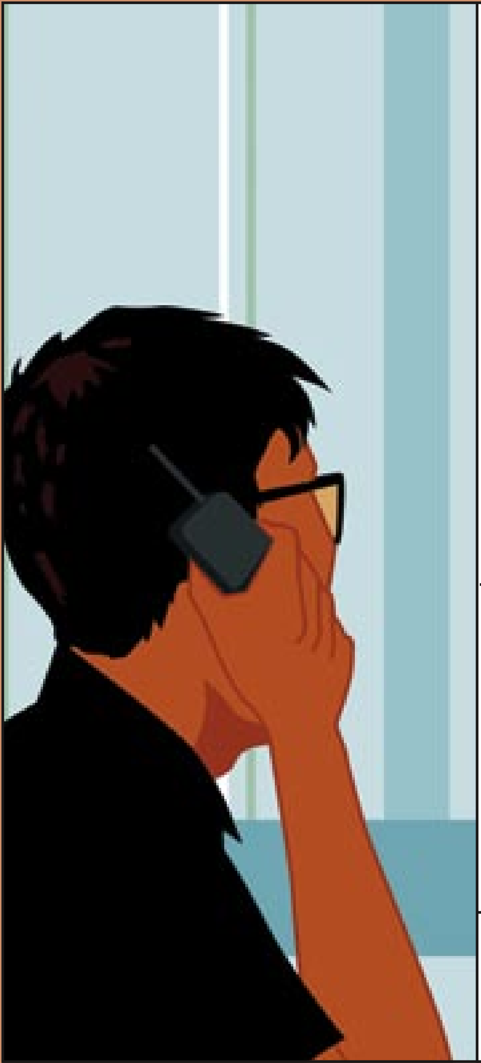

Segment Description

The rich kids have moved out of these playgrounds.

These mainly major city segments have had serious downward shifts in relative income. The top earning bracket has declined by 37% since 1991and those in the lowest bracket have increased by 43%.

That's a massive shift, and it's due mainly to a 70% increase in people aged 65 moving there from 1991 to 2001. There are also 30% more families with non-dependent kids and students aged 15-24 living at home.

Lots of kids are dropping out of school here at year 11 to pursue work in manufacturing or communication services and many people are employed in trades, elementary sales and intermediate jobs. Unemployment is at an average level. Household incomes are average across all bands.

Interests include Pay TV, Sunday Newspapers, football, gambling, motor car racing, puzzles and crosswords.

Defining Features

1. Although the income distribution for this segment is average, there have been significant decreases over the last 10 years
2. There has been a 70% increase in the proportion of people aged over 65
3. The presence of families with older children and non dependent children are well above average
4. 3/4 of the households who rent, pay less than $200 a week and 57% of households with mortgages pay less than $1000 per month.

Key Expenditure

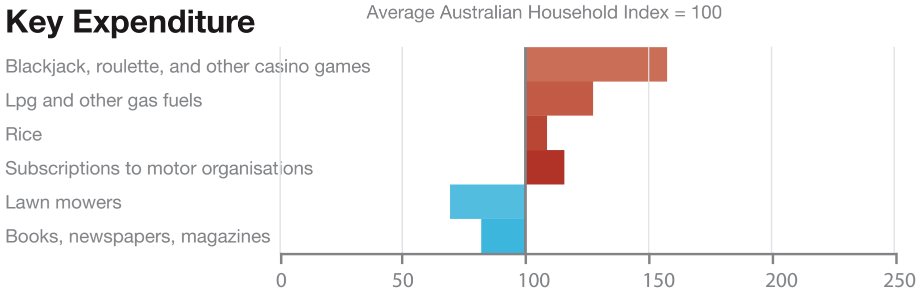

Population Profile

93% of the 662,911 people that live in this segment live in Major Cities.

Location Map

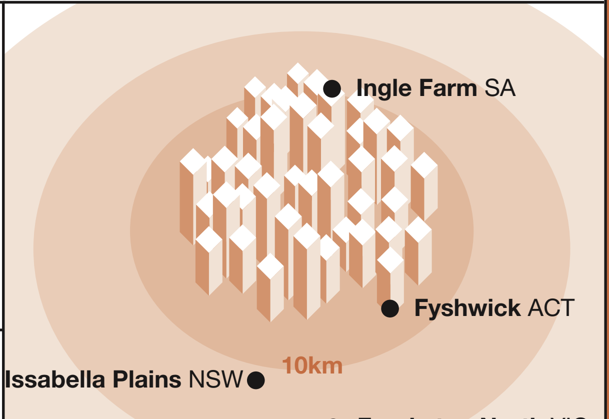

Pathfinder Solutions © Copyright 2003 /// phone: +61-3-9670 3030 /// email: info@pathfindersolutions.com.au

PATHFINDER SEGMENTS OF AUSTRALIA

27 Hospital Precincts and Aged Care

Lawn bowls and telly

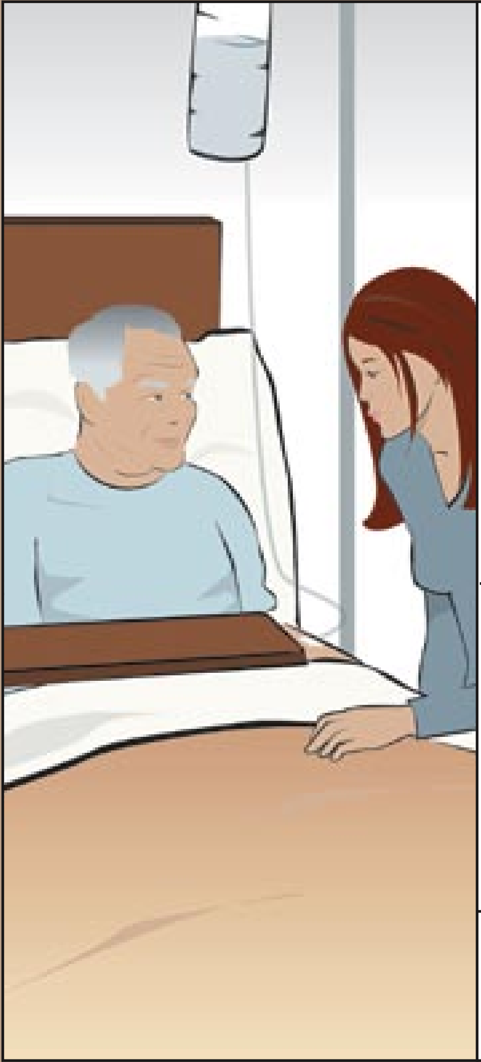

Segment Description

This segment incorporates the old timers who live in residential care. The segment also includes staff that work in hospitals and live on site.

The set comprises around 32% who have moved from their original residences into maintained venues.

Household incomes are 1.3 times as likely to be less than $15,000 per year, but otherwise household incomes are average across all bands.

Development of commercial buildings such as nursing homes has been significant over the past ten years.

There is no great expenditure here from these people who delight in the simple things such as watching television and playing lawn bowls.

Naturally, this segment watches pay TV and lawn bowls whenever they can.

Defining Features

1. This segment has 4 times the national average of people aged 85+
2. 14% of women work in healthcare
3. Household incomes are average across most bands (although over represented in the $5,000-$15,000 p.a. band)
4. 32% of residents in this segment live in non-private accommodation

Key Expenditure

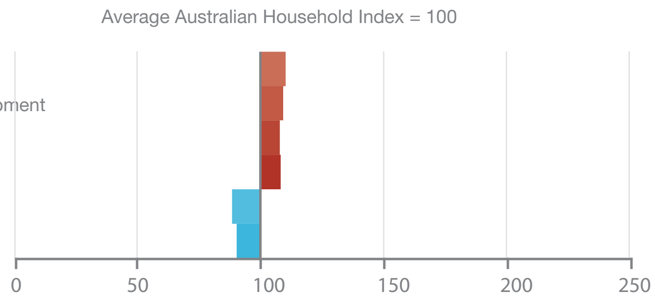

Population Profile

51% of the 267,263 people that live in this segment live in Major Cities, 26% live in Inner Regional, 18% live in Outer Regional and 5% live in Remote areas.

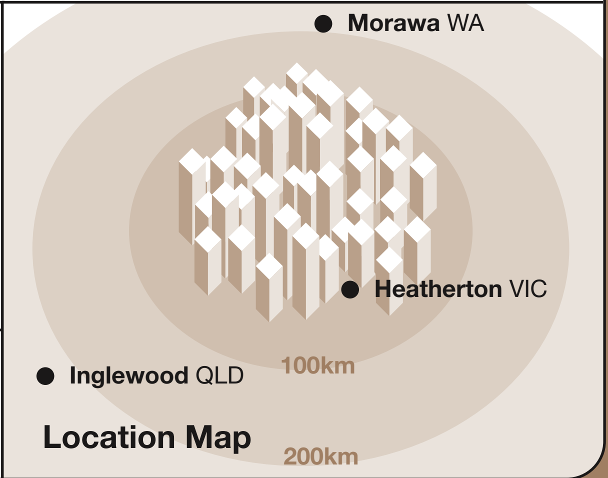

Location Map

Pathfinder Solutions © Copyright 2003 /// phone: +61-3-9670 3030 /// email: info@pathfindersolutions.com.au

PATHFINDER SEGMENTS OF AUSTRALIA

28 Living Alone

Me, myself, I

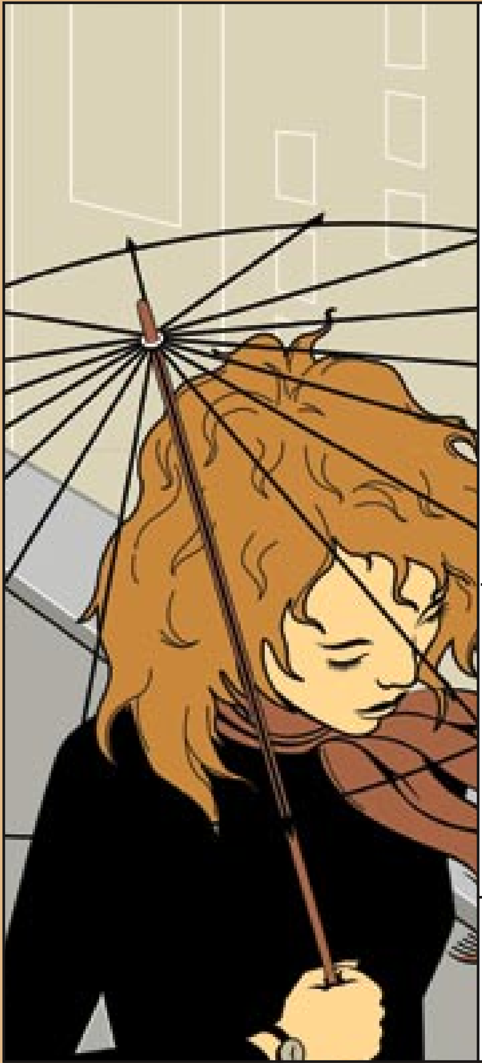

Segment Description

Around a third of the people in this segment opt to live alone.

You are 1.3 times as likely to find people aged 70 plus in this segment. Personal incomes are average across most bands with the exception of the very highest band, which is under represented.

Household income levels of less than $15,000 per year are over represented, and they have one car or no car.

They don't usually own a home but are likely to rent a flat, unit or apartment, with rent payable being in the $50 to $150 per week bracket.

From 1988-1996 there was a boom in commercial development to suit these types of people, with an increase in dual occupancy developments over the past five years.

The preferred mode of transport is walking, as they are less likely to own a motor vehicle. Travel by taxi is also over represented in this segment.

Defining Features

1. Around 1/3 of people in this segment live alone
2. 17% of the families in this segment are single parent families
3. Flats, units and low rise apartments are common
4. 18% (1.4 times the average) of people in this segment are aged over 65
5. 75% of people were born in Australia

Key Expenditure

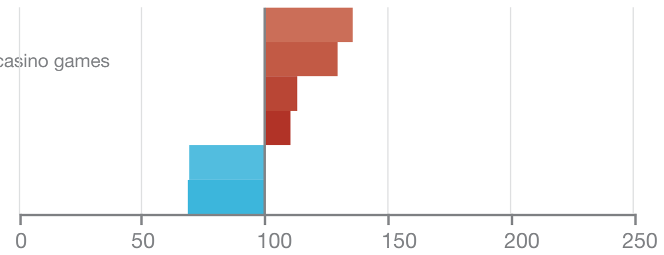

Population Profile

61% of the 391,885 people that live in this segment live in Major Cities, 23% live in Inner Regional, 11% live in Outer Regional and 5% live in Remote and Very Remote areas.

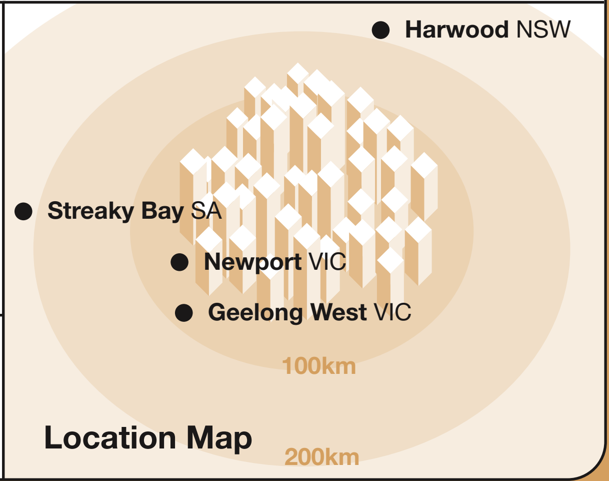

Location Map

Pathfinder Solutions © Copyright 2003 /// phone: +61-3-9670 3030 /// email: info@pathfindersolutions.com.au

PATHFINDER SEGMENTS OF AUSTRALIA

29 Multicultural Mix

The melting pot

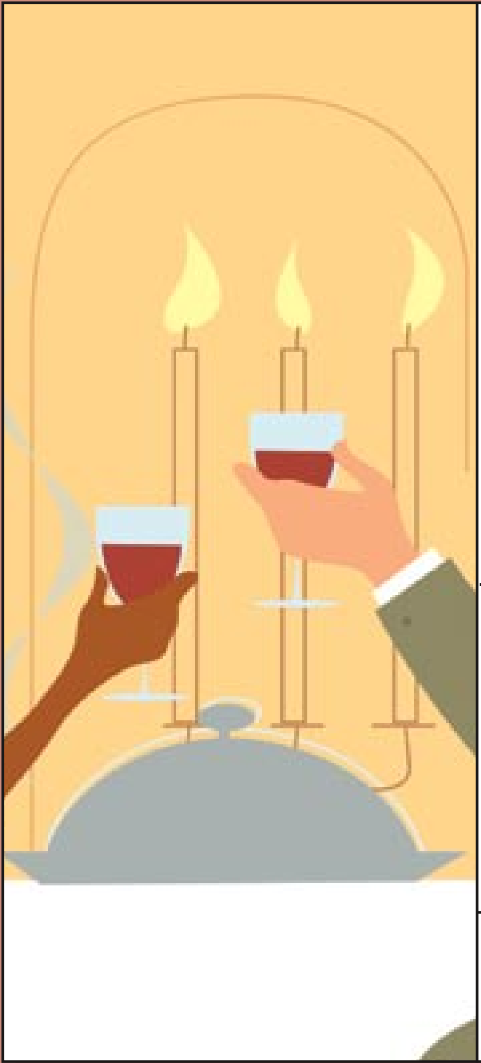

Segment Description

Hailing from countries such as Italy, Greece, Turkey and Vietnam, this segment comprises almost 43% who were born overseas. Most of this diverse group arrived on our fair shores over 15 years ago (60%) and over half speak a language other than English at home.

Many follow Catholic, Greek Orthodox and Oriental Christian, Islam or Buddhist faiths.

They love a good, big family and there are often five or more to a household. Catholic schooling is preferred and TAFE education is common. Many adults never attended or completed school themselves.

Upholding the stereotype, many live in flats or houses attached to shops. Over half fully own their home and monthly mortgage repayments of $1,500 to $2,000 are common.

Unemployment is a problem at 20% above the national average. Household income levels are however average across all bands. Otherwise, manufacturing accounts for 23% of jobs with labouring and intermediate jobs also being common.

Spending is high on food of every kind as well as furniture, betting at the TAB, sports lessons, travel goods and private education tuition fees.

Defining Features

1. 55% of people in this segment speak a language other than English at home
2. 60% of people born overseas arrived in Australia more than 15 years ago
3. Many were born in Southern Europe, the Middle East and Asia.
4. More than half the homes are fully owned
5. Unemployment levels are 20% above the national average
6. Around 20% of adults in this segment didn't attend school, or finished before year 8

Key Expenditure

Average Australian Household Index = 100

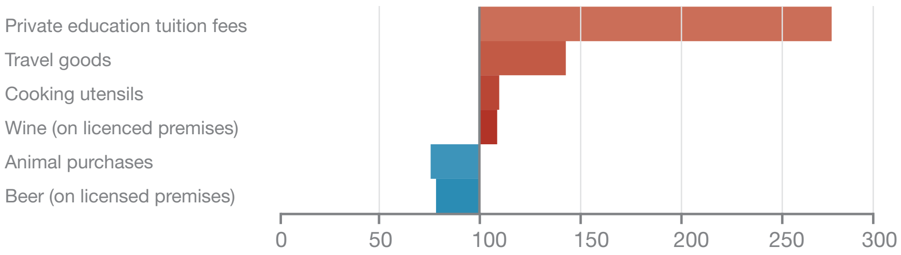

Population Profile

99% of the 162,561 people that live in this segment live in Major Cities.

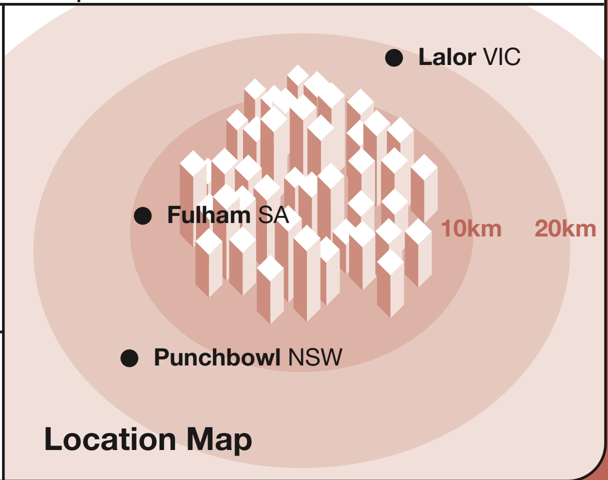

Location Map

Pathfinder Solutions © Copyright 2003 /// phone: +61-3-9670 3030 /// email: info@pathfindersolutions.com.au

PATHFINDER
SEGMENTS
OF AUSTRALIA

30 Established UK Migrants

Jolly good ol' chap!

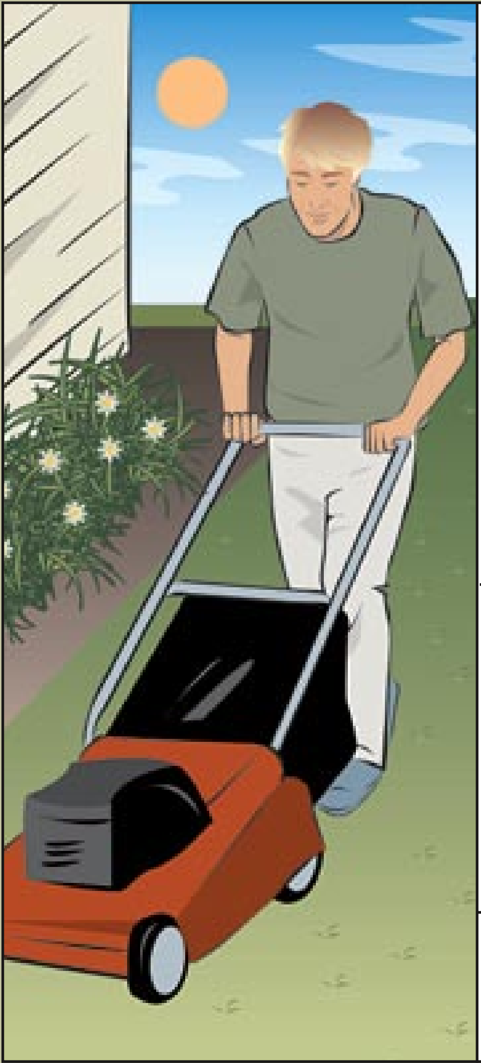

Segment Description

UK born chaps and lasses can be found in major cities and regional areas, over three quarters of whom arrived in Australia at least 15 years ago.

They've been pipped from their top posts it seems with those with top incomes declining 25% from 1991 to 2001. Those in the second and third top brackets have, however, risen significantly (12% and 6% respectively).

God is of little interest to them with almost a quarter of this group having no religion - double the Australian average.

Household incomes are fairly average, but these guys are not the richest of the rich or the poorest of the poor with the highest and lowest income bands being under-represented. Many have mortgages (36%) with repayments usually below $800 per month. They love fixing up their homes, adding carports, garages, pergolas and verandas.

Many work in manufacturing, but around a quarter of the chaps are more likely to be found working in the trades. They spend their pounds and pence on meat, outdoor furniture, carpets, overseas holidays and pets.

Defining Features

1. 15% of people were born in the UK
2. Over 80% of those born overseas arrived in Australia at least 15 years ago
3. Almost 1/4 of this segment have no religion
4. Incomes are around the average although both the highest and lowest bands are under-represented

Key Expenditure

Average Australian Household Index = 100

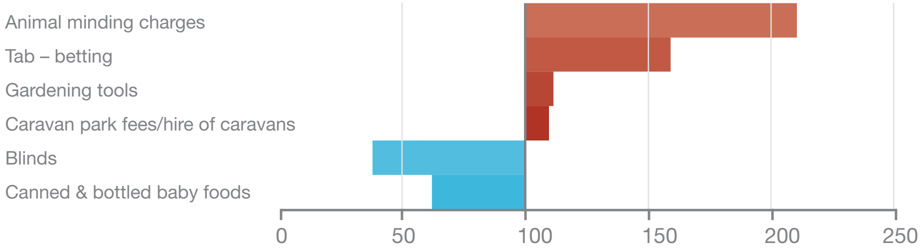

Population Profile

57% of the 237,021 people that live in this segment live in Major Cities, 32% live in Inner Regional and 10% live in Outer Regional areas.

Location Map

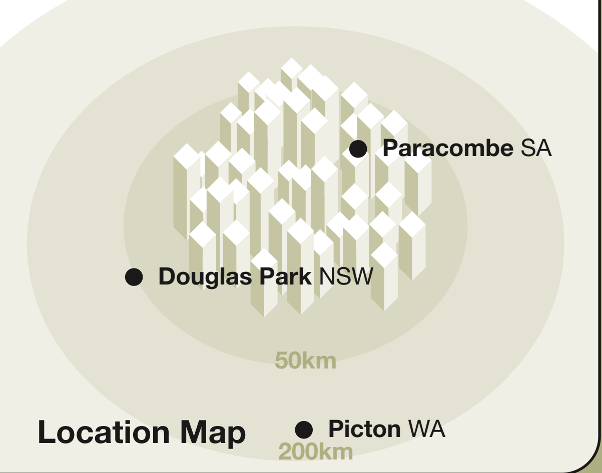

Pathfinder Solutions © Copyright 2003 /// phone: +61-3-9670 3030 /// email: info@pathfindersolutions.com.au

PATHFINDER SEGMENTS OF AUSTRALIA

31 Repeat Movers

Itchy feet

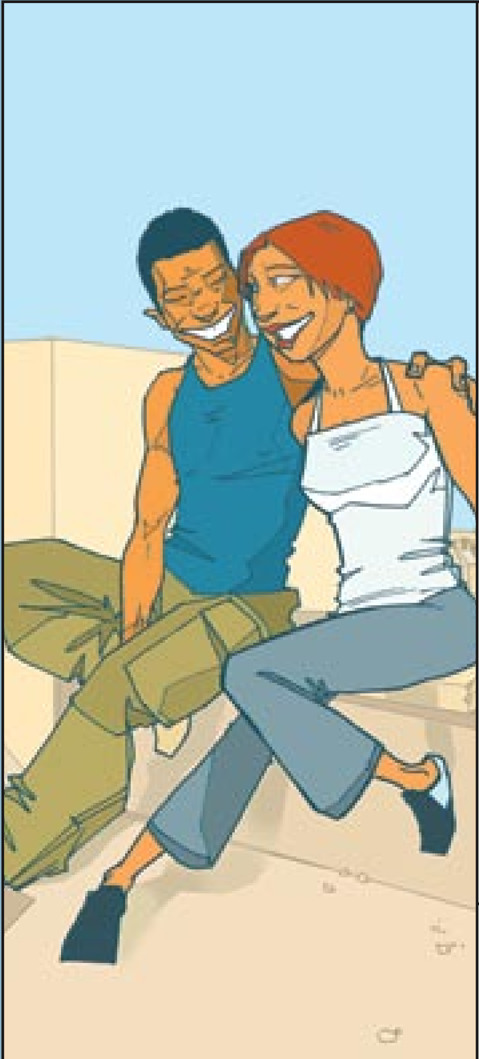

Segment Description

These guys can't stay still! Around a quarter of them were at a different address the previous year.

They're a sporty, active bunch preferring the pub and the gym to other recreational activities. Not surprisingly the strongest age groups are from 20 to 29, however the over 70s are also over represented. They are likely to be a combination of divorced, separated, widowed, never married, de facto or single parents. Many are students and over half the segment live in flats, units, apartments or townhouses in share arrangements or alone.

It's a renter's world with most forking out below $150 per week. Public housing counts for around 10%. Household incomes are 1.5 times as likely to be below $15,000 per year and the unemployment rate in this group is 36% above the national average. Around 11% were born overseas and many of these are recent arrivals.

They're more likely to get to work by public transport, motorbikes, bicycles or travelling in a car as a passenger than owning their own car.

Defining Features

1. Ages 20 –29 are very strong as are the over 70's
2. More than 3/4 of the residents live in units or townhouses
3. Unemployment rate is 36% above the national average
4. The proportion of single people is very high (includes divorced, separated and widowed)
5. There is a strong presence of both group and lone person households

Key Expenditure

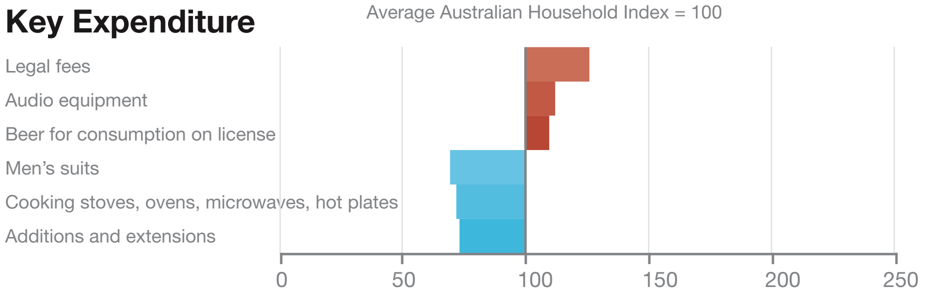

Population Profile

62% of the 655,640 people that live in this segment live in Major Cities,
22% live in Inner Regional, 13% live in Outer Regional and 3% live in Remote areas.

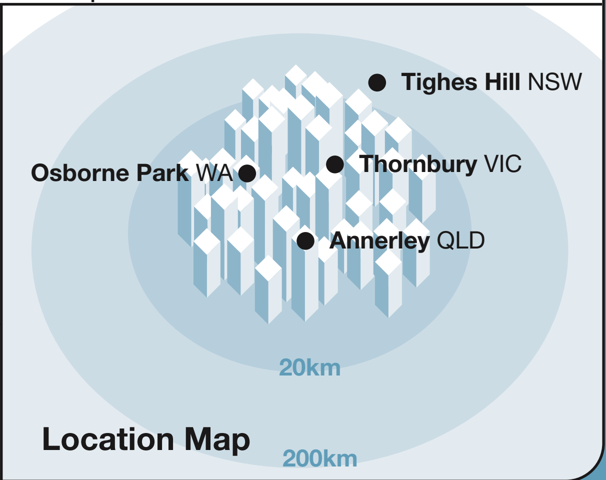

Location Map

Pathfinder Solutions © Copyright 2003 /// phone: +61-3-9670 3030 /// email: info@pathfindersolutions.com.au

PATHFINDER SEGMENTS OF AUSTRALIA

32 High Rise Rentals

Going up?

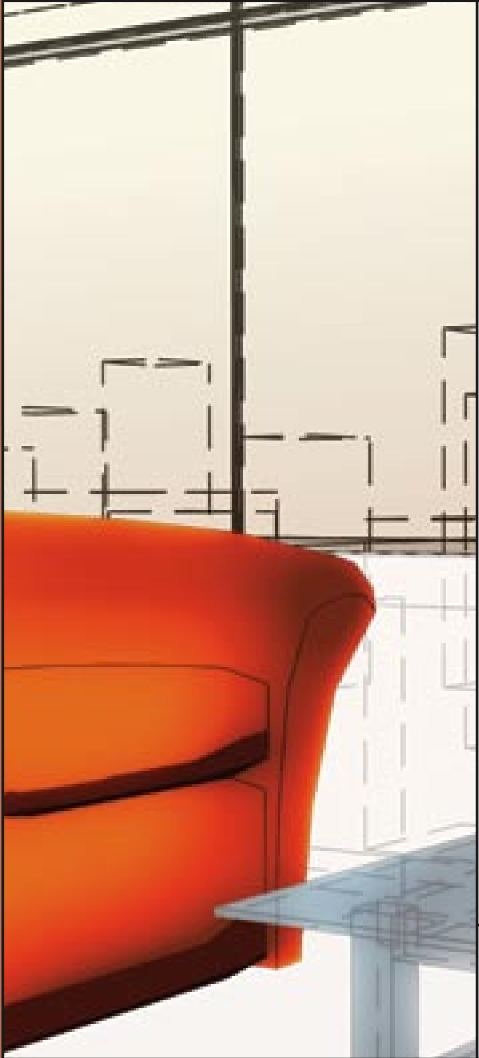

Segment Description

This segment is mainly Australian migrants who live in flats and apartments in and around major cities. More than double the national average of TAFE and University students live here. Almost 40% were born overseas and 34% speak a language other than English at home, 8% of which speak Chinese.

They are mainly aged between 20 and 34, an above average number having been divorced or separated. In fact, there's a very low rate of married people, although there is an above average rate of de facto couples, group and lone person households.

Over three quarters live in flats and apartments with a mixture of government and private rental. Weekly rents are mainly around $200 to $250. Unemployment is 60% above national levels here. Household incomes are 1.5 times as likely to be less than $15,000 per year.

Interestingly, a significant proportion of this segment has a bachelor and postgraduate degree, which shows, once again, that "the piece of paper doesn't mean much". People are generally employed in industries such as hospitality, cultural & recreational services, business, property and financial services.

Use of public transport is high and more than a quarter of households have no car.

Defining Features

1. Around 3/4 of the housing in this segment is made up of flats and apartments
2. There is a mixture of state and private rentals (10% and 50% respectively)
3. The number of people with bachelor degrees is 40% over the average
4. 40% of people were born overseas and more than 2/3 of the migrants have arrived in Australia in the last 10 years
5. There are strong skews towards 20 –34 year old age groups

Key Expenditure

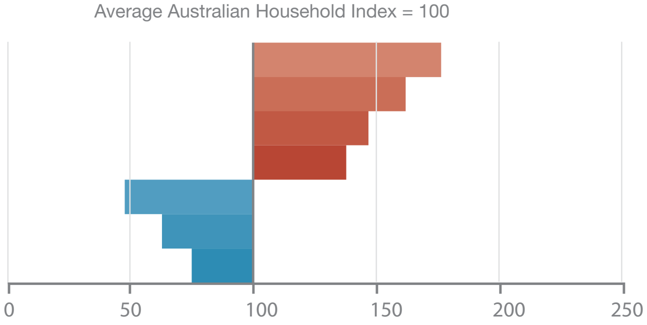

Population Profile

90% of the 491,829 people that live in this segment live in Major Cities, and 10% live in Inner and Outer Regional areas.

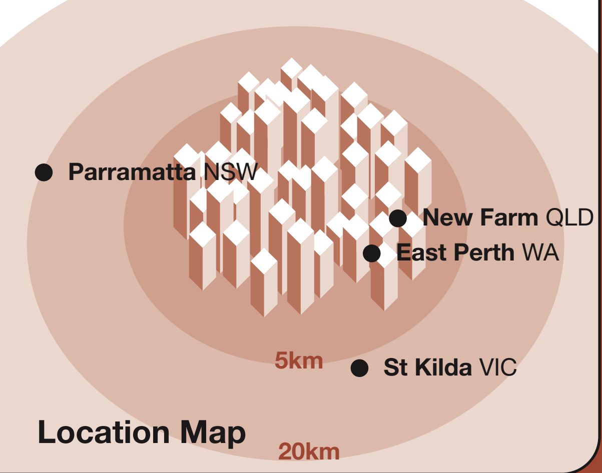

Location Map

Pathfinder Solutions © Copyright 2003 /// phone: +61-3-9670 3030 /// email: info@pathfindersolutions.com.au

PATHFINDER SEGMENTS OF AUSTRALIA

33 Middle Eastern Migrant Enclaves

Multicultural hubs

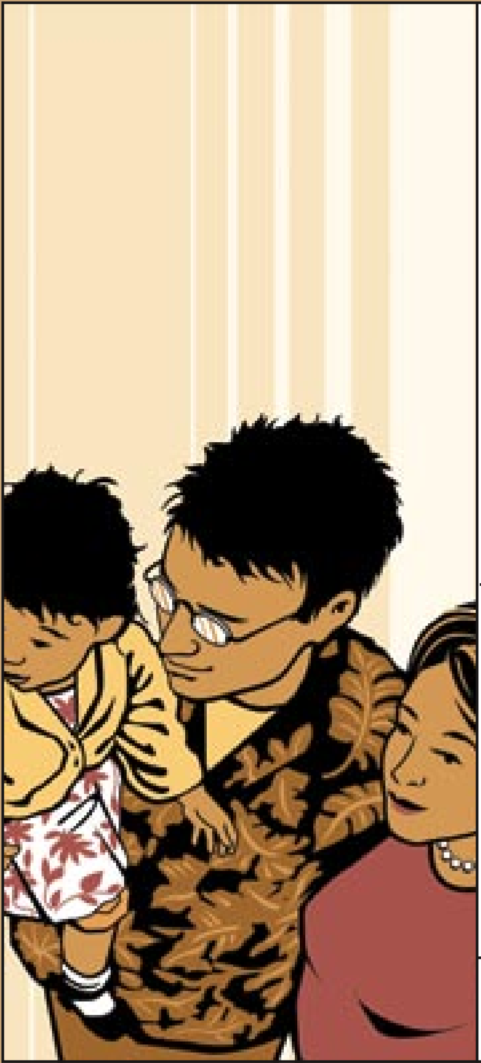

Segment Description

This is a young, multicultural group with 44% having been born overseas and a huge 60% speaking a language other than English at home – 24% speaking Arabic. More than 16% were born in the Middle East. There are lots of people under 25 years of age.

Significant numbers of these migrants arrived in Australia between 1990 and 1996. Islam accounts for 21% of the population and Buddhism, Hinduism, Oriental Christian, and Catholic faiths are common.

They generally send their kids to non-government primary schools and Catholic secondary schools. University study is not as commonly pursued as TAFE study.

Household incomes are generally average across all bands; however unemployment is high at 53% above national rates. People mainly have jobs in transport, construction, manufacturing, communications, trades, intermediate skilled jobs and labouring.

They love Pay TV and to watch sport, but interest in participating in active sports is low at around 5%.

Defining Features

1. This segment has high ethnicity with 60% speaking a language other than English at home (24% speak Arabic)
2. 44% of people were born overseas
3. All age groups under 25 are over represented
4. Private school and Catholic school attendances are over represented.
5. Unemployment rates are 53% above the average
6. Household incomes are average
7. They have very strong magazine and Pay TV subscription rates

Key Expenditure

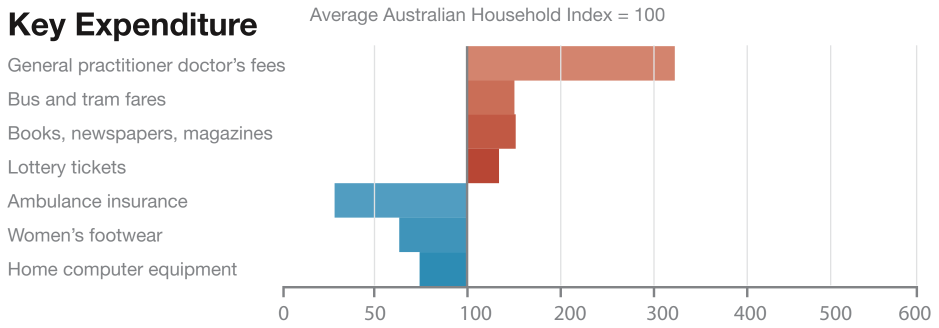

Population Profile

99% of the 152,686 people that live in this segment live in Major Cities.

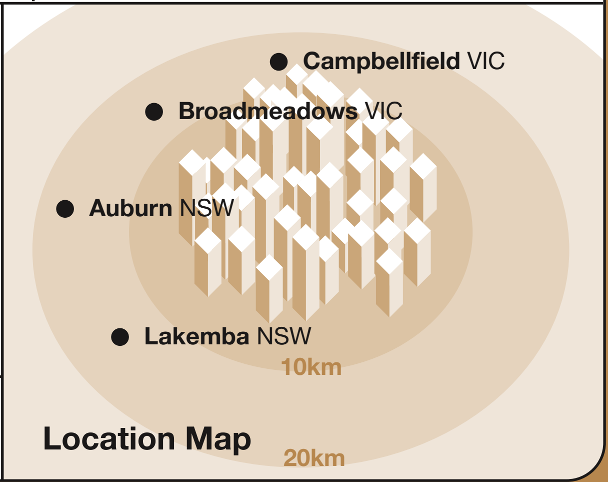

Location Map

Pathfinder Solutions © Copyright 2003 /// phone: +61-3-9670 3030 /// email: info@pathfindersolutions.com.au

PATHFINDER SEGMENTS OF AUSTRALIA

34 Young Single Parent Families

Splitsville

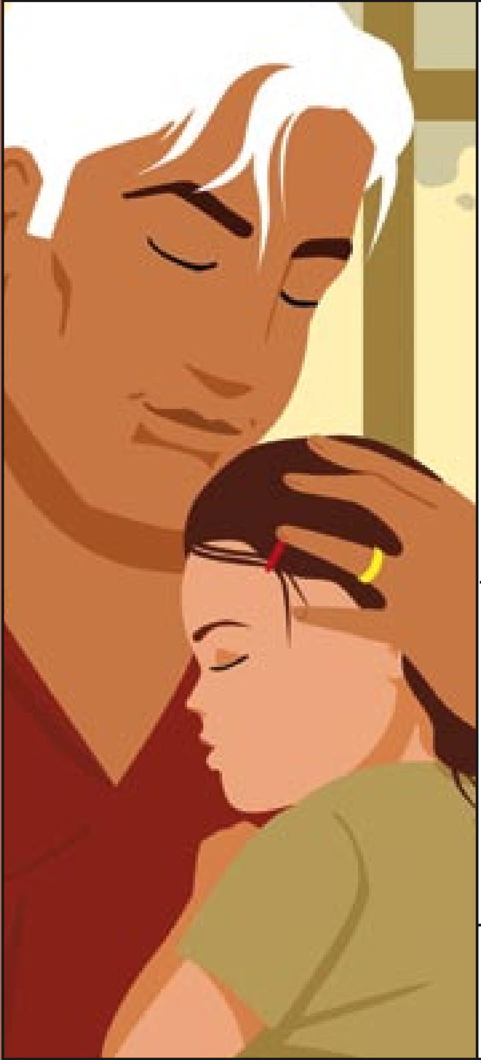

Segment Description

Over a quarter of families in these areas are single parent. A high proportion are separated or divorced and have kids under 15 years of age. You are much less likely to find people speaking languages other than English at home.

Dropping out of school at year 9 or 10 is common as are yearly household incomes of around $20,000-$40,000. Mortgages are prevalent with monthly loan repayments generally around $200 to $800, and 83% of people live in separate houses.

Unemployment rates are high at 40% above average. There's not enough money for home extensions and improvements, with the exception of the occasional addition of carports and garages. They have above average expenditure on items such as milk and sausages - hardly the lap of luxury!

Crafts, country music and fishing are common interests.

Defining Features

1. Around 3/4 of all families are single parent families
2. It is common for schooling to have finished in year 9 or 10
3. Unemployment rates are 40% above the national average
4. Age groups under 14 years are slightly over represented, and the oldest groups are under represented
5. Overseas born are not skewed towards any particular country and overall are under represented
6. Household incomes are strong in the lower bands

Key Expenditure

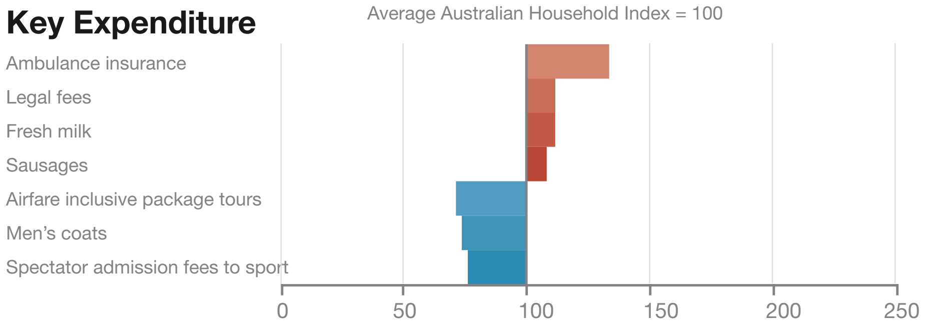

Population Profile

71% of the 213,626 people that live in this segment live in Major Cities, 19% live in Inner Regional and 8% Outer Regional areas.

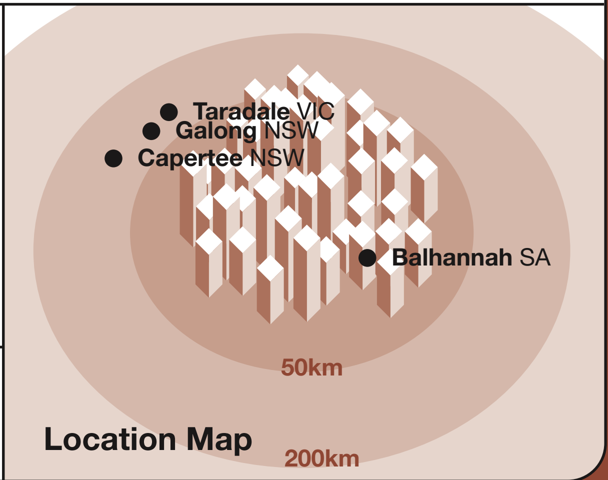

Location Map

Pathfinder Solutions © Copyright 2003 /// phone: +61-3-9670 3030 /// email: info@pathfindersolutions.com.au

PATHFINDER SEGMENTS OF AUSTRALIA

35 Vietnamese Migrant Enclaves

Little Saigon

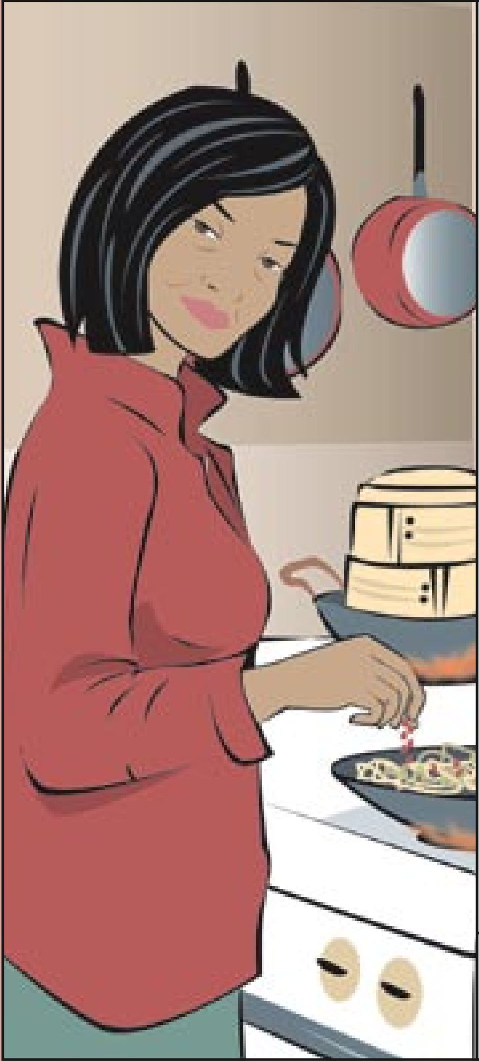

Segment Description

These are high migrant areas with a heavy Vietnamese presence where around 45% were born overseas and well over half speak a language other than English at home.

These major city segments contain a mixture of Buddhist, Oriental Christian and Islamic faiths and a great proportion arrived in Australia between 1986 and 1995.

These are big families of commonly five to six people, mainly couples with both dependent and non-dependent children. To round it off they love pets, with around a quarter of households having a family pet.

Women have particular trouble getting work and the general unemployment rates are almost double the average. Household incomes of $15,000 to $20,000 per year are over represented. Extensions and enhancement to property are very low; however dual occupancy development was high between 1998 and 2000.

Defining Features

1. Over a quarter of all of Australia's Vietnamese born residents live in this segment
2. 17% of people speak Vietnamese at home
3. There are many couple families with both dependent and non-dependent children
4. Households with 5 or more people are common
5. 46% own their homes outright, and subsequently this segment is quite stable
6. Household incomes are roughly average, although are under represented in the top two income bands

Key Expenditure

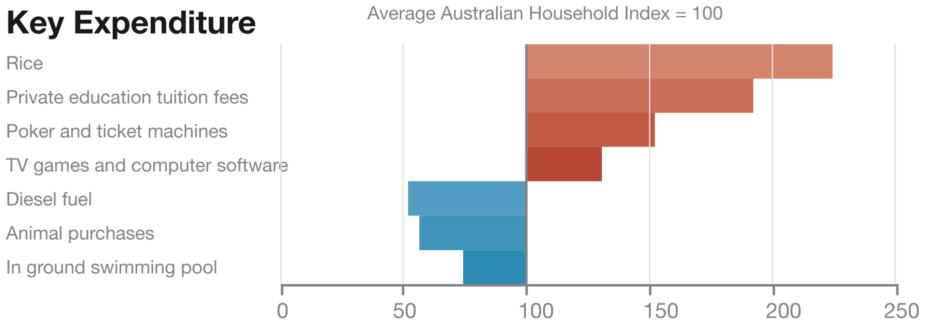

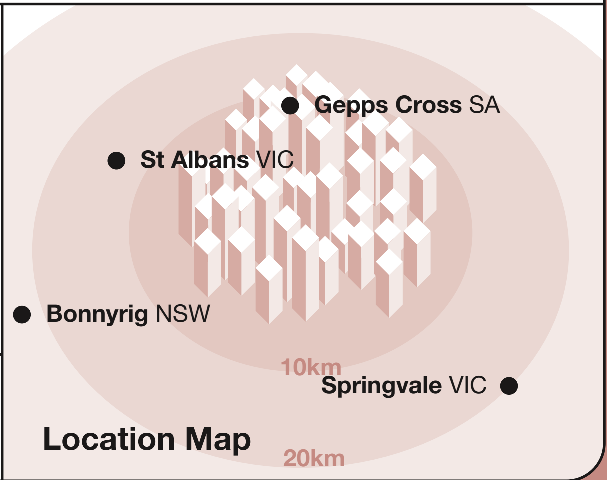

Location Map

Population Profile

All 321,627 people that live in this segment live in Major Cities.

Pathfinder Solutions © Copyright 2003 /// phone: +61-3-9670 3030 /// email: info@pathfindersolutions.com.au

PATHFINDER SEGMENTS OF AUSTRALIA

36 Unskilled Battlers

Just getting by

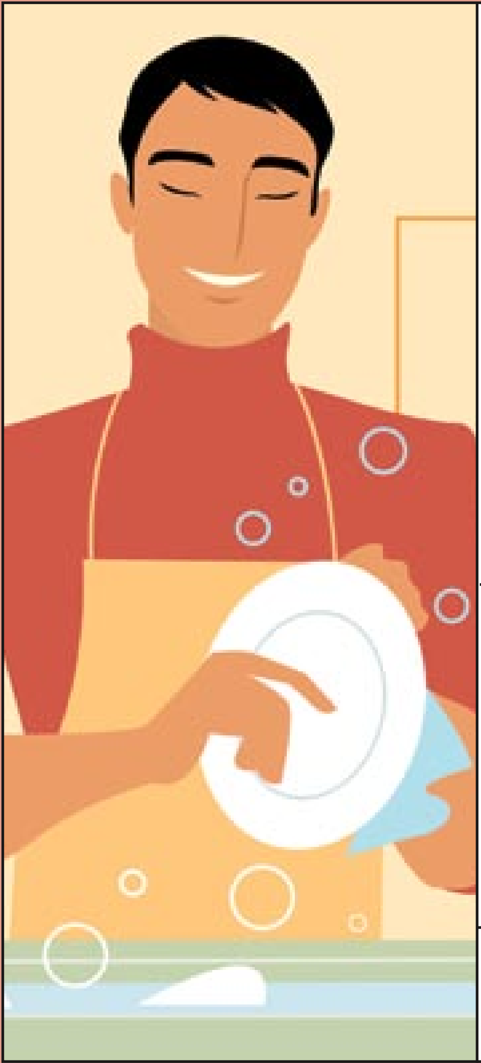

Segment Description

Found in major cities and regional areas, this is a bunch that struggle to get by.

You are 1.3 times as likely to find people aged 65 plus in this segment, and indigenous Australians are over represented.

Most are unemployed (64% more than average) and many are single parent families (the rate is 29% higher than the norm).

Personal incomes of between $6,000 and $15,000 per annum are over represented, and about a third of households earn less than $30,000 a year. Many left school in year 10 or before. Labouring, elementary clerical, sales and service, intermediate production and trades are the most common jobs.

Monthly loan repayments up to $800 are common, as are weekly rent payments under $150.

Building activity, extensions and home improvements in these areas have remained low for the past 10 years.

Pay TV, lawn bowls and country music are the major drawcards for these people.

Defining Features

1. Around 1/3 of households have an average income below $20,000 p.a.
2. Education levels are strongly skewed towards completion of schooling at year 10 or earlier (25% finished at year 9 or below)
3. Unemployment rates are 64% above the national average
4. Rents and mortgage payments are strong in the lowest bands
5. Occupations are typically non-skilled
6. Over 3/4 of all households have one or no car

Key Expenditure

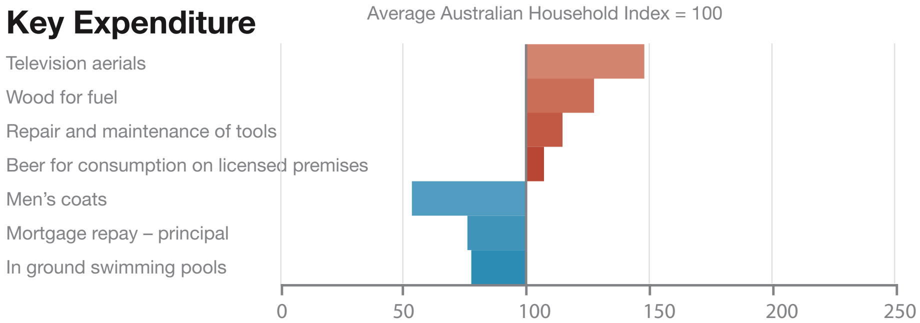

Population Profile

50% of the 445,750 people that live in this segment live in Major Cities, 30% live in Inner Regional and 19% live in Outer Regional areas.

Location Map

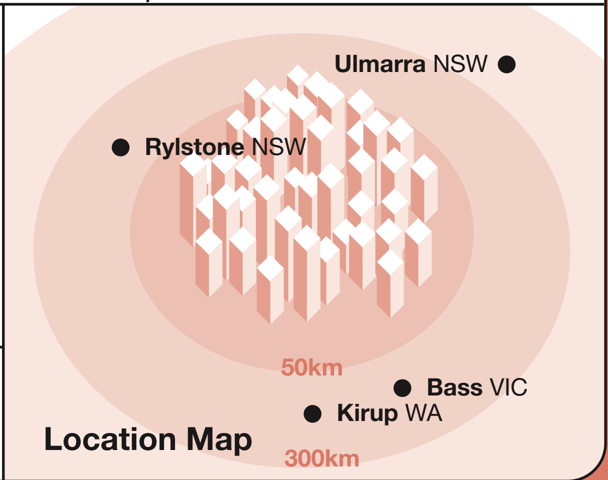

Pathfinder Solutions © Copyright 2003 /// phone: +61-3-9670 3030 /// email: info@pathfindersolutions.com.au

PATHFINDER SEGMENTS OF AUSTRALIA

37 Single Parent Public Housing

Struggling Solo

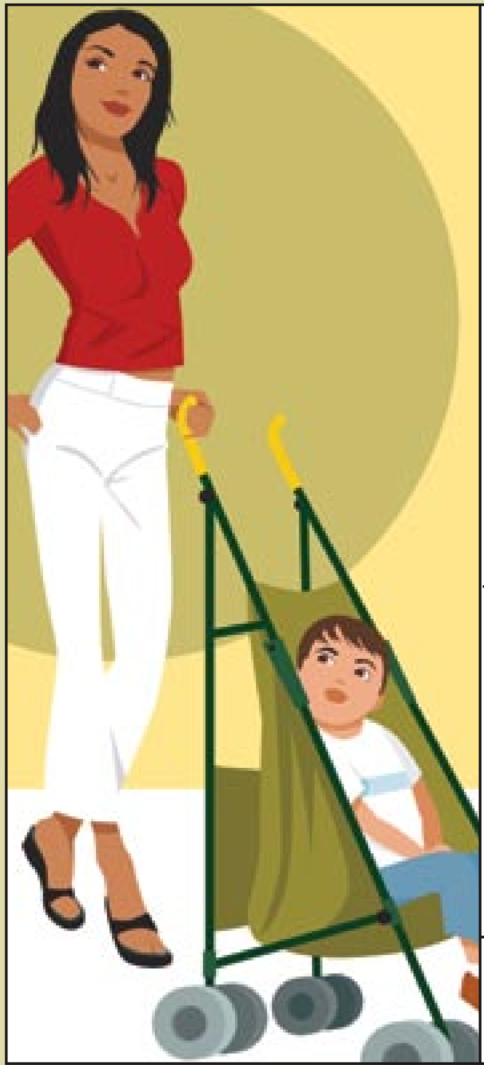

Segment Description

Divorcees and separated couples are common in these major city and regional areas. Indigenous people are over represented here.

Around a third of families have just one parent, and around a third live in Public Housing, and pay rent of less than $100 per week. Housing in flats and apartments is most common.

Kids are young – mainly under nine years of age. Many drop out of school at around Year 8, 9 or even below.

Unemployment is over double the national average and if people have jobs, they are not highly skilled and earn them typically less than $15,000 per year.

Public telephones are used a lot and money is often spent on audio equipment, heating oil and gambling.

Defining Features

1. 1/3 of families in this segment are single parent families
2. 17% of residents are under the age of 9
3. Income levels are typically below $30,000 p.a.
4. 17% of people over the age of 15 are divorced or separated
5. Nearly 3/4 of all people finished high school at year 9 or earlier
6. There is above average attendance at full time TAFE
7. This segment accounts for over 1,000,000 Australians

Key Expenditure

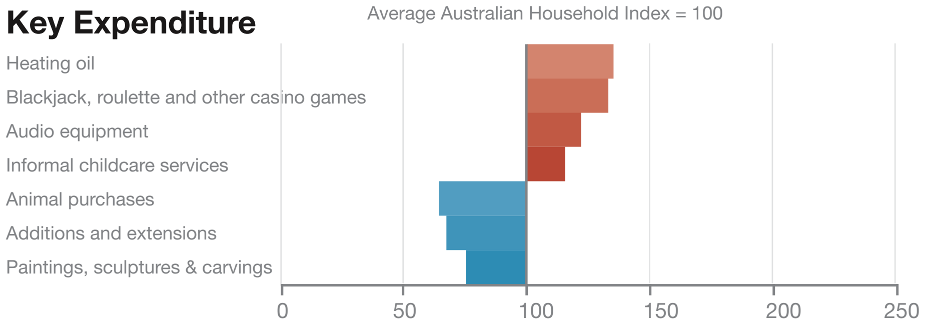

Population Profile

69% of the 1,031,967 people that live in this segment live in Major Cities, 19% live in Inner Regional and 9% live in Outer Regional areas.

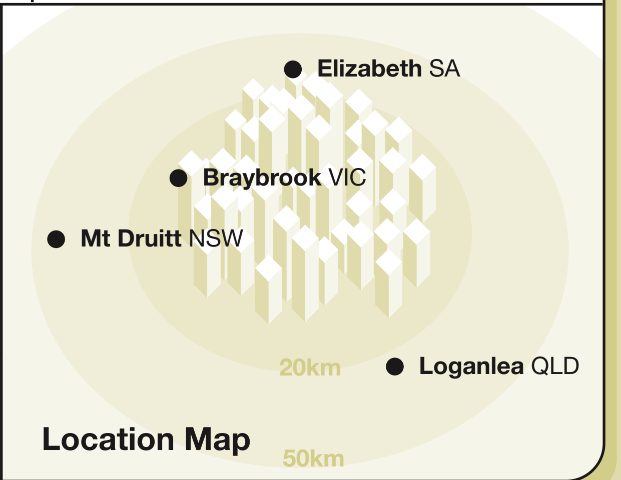

Location Map

Pathfinder Solutions © Copyright 2003 /// phone: +61-3-9670 3030 /// email: info@pathfindersolutions.com.au

PATHFINDER SEGMENTS OF AUSTRALIA

38 Thriving Regional Living

Working on the land

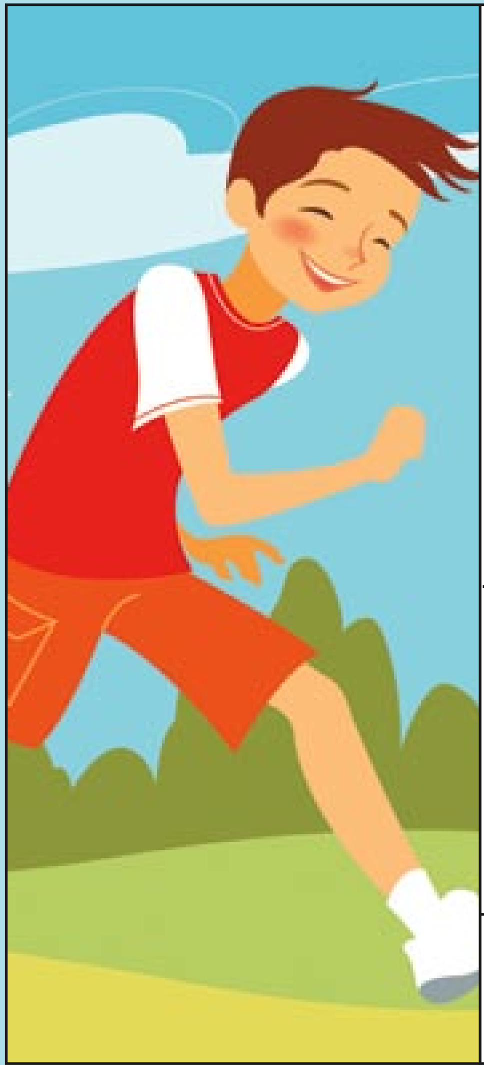

Segment Description

This segment thrives on the land with regional allotments of generally around two hectares in size.

Most of these people are born in Australia with under 5% speaking a language other than English at home.

They're mostly big families of four to six who stick together - most are couples with kids aged generally up to 19 years. There aren't many people aged from 20 to 29 or over 60 here, and there are low levels of full-time University students. Many leave school in Year 10 or 11 to pursue certificate qualifications in agriculture, engineering or education.

Many have a mortgage with monthly repayments of around $800 to $999. Most households have at least two vehicles, and spend a lot on petrol. Households earning between $60,000 and $100,000 per year are over represented here.

There was a boom in extensions here in the mid 1990s. People spend money on lawnmowers, pools, outdoor furniture and other outside improvements.

Defining Features

1. Family incomes above $52,000 p.a. are over represented
3. Regional allotments around 2 hectare in size are common
4. There is a strong presence of couple families with children
5. The most common monthly mortgage repayment is the $800 - $1000 band

Key Expenditure

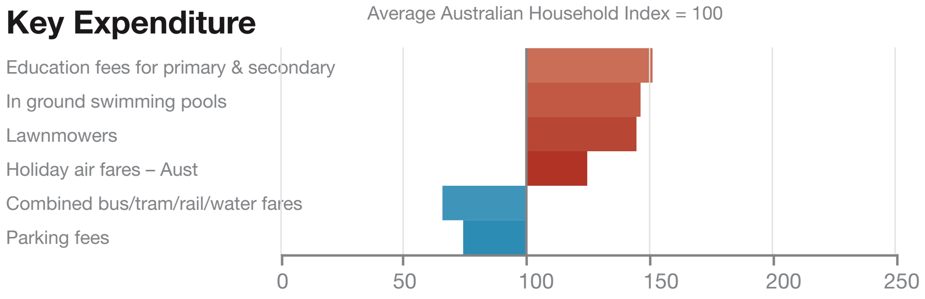

Population Profile

55% of the 510,224 people that live in this segment live in Inner Regional areas, 21% live in Outer Regional, 16% live in Major Cities and 8% live in Remote areas.

Location Map

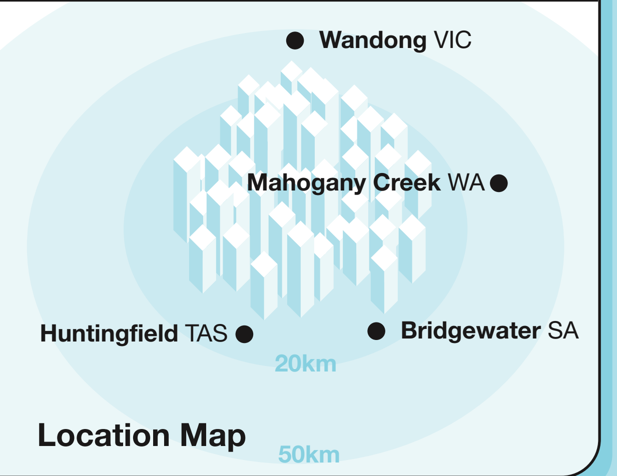

Pathfinder Solutions © Copyright 2003 /// phone: +61-3-9670 3030 /// email: info@pathfindersolutions.com.au

PATHFINDER SEGMENTS OF AUSTRALIA

39 Small Farms & Regional Lifestyle

The good country life

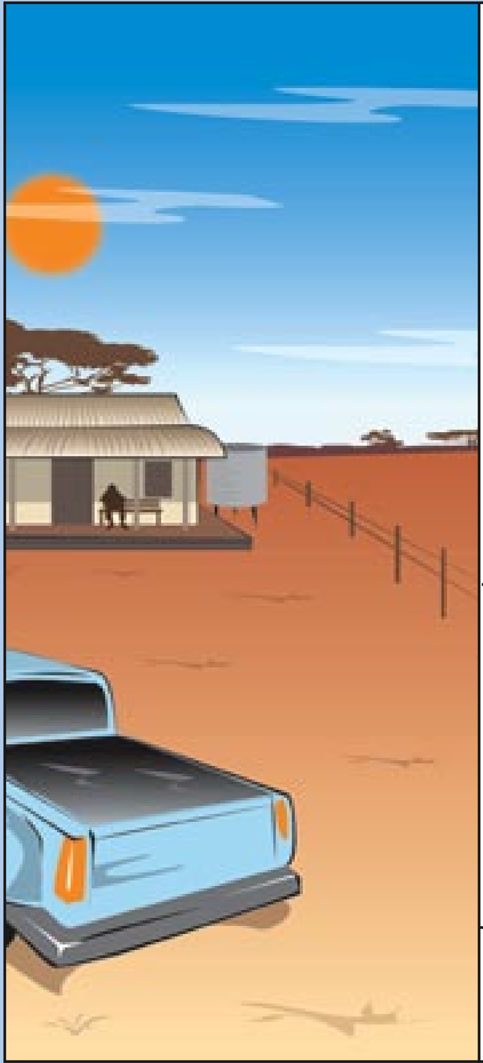

Segment Description

Most of the people in this mainly regional segment are happy, married families with kids aged 10-14 and parents around 40-54. Divorce and separation rates are low with over 60% of people being married.

They live on farms and stay there – most having lived at the same address for over five years. There is virtually no such thing as renting and almost all homes are separate houses that are owned outright or mortgaged. In the last 10 years, lots of homes have been built and extensions taken place. A quarter of homes have three or more vehicles.

Education levels are pretty standard with more dropping out between Years 9 and 11. Around 15% work in agriculture and many in this segment work in utilities such as gas, water and electricity. Around 10% work from home.

Household incomes are generally average across most bands, although those earning less than $15,000 per year are under represented.

Defining Features

1. 15% of people work in agriculture
2. There has been strong growth in home building over the past 10 years
3. Over 60% of people aged over 15 are married
4. The 10-14 and 40 – 54 year age bands are over represented
5. Almost all homes are separate houses, and are either owned outright or currently being purchased

Key Expenditure

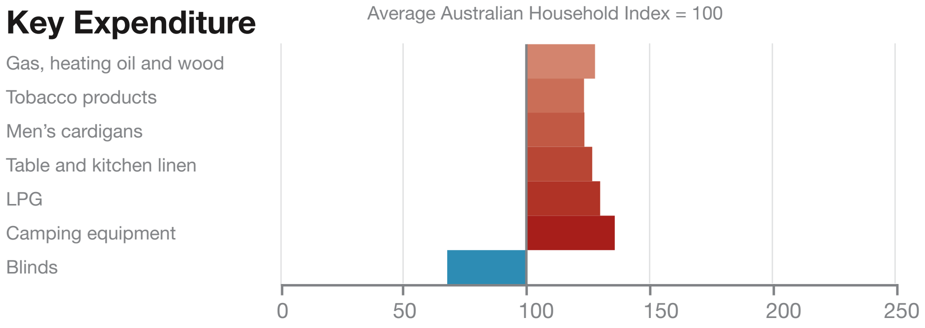

Population Profile

71% of the 123,414 people that live in this segment live in Inner Regional areas, 20% live in Outer Regional, and 6% live in Major Cities.

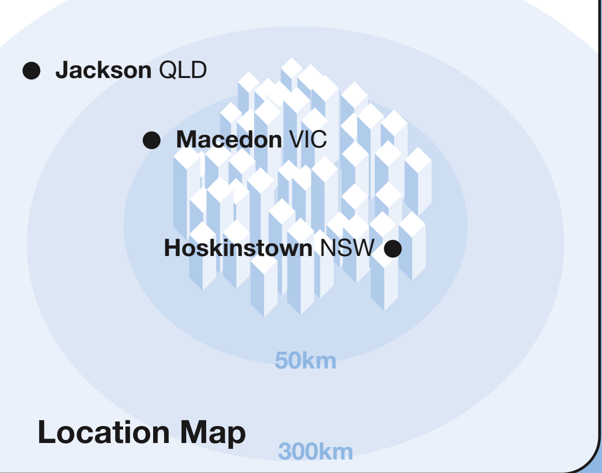

Location Map

Pathfinder Solutions © Copyright 2003 /// phone: +61-3-9670 3030 /// email: info@pathfindersolutions.com.au

PATHFINDER
SEGMENTS
OF AUSTRALIA

40 Rising Country

Young bumpkins'

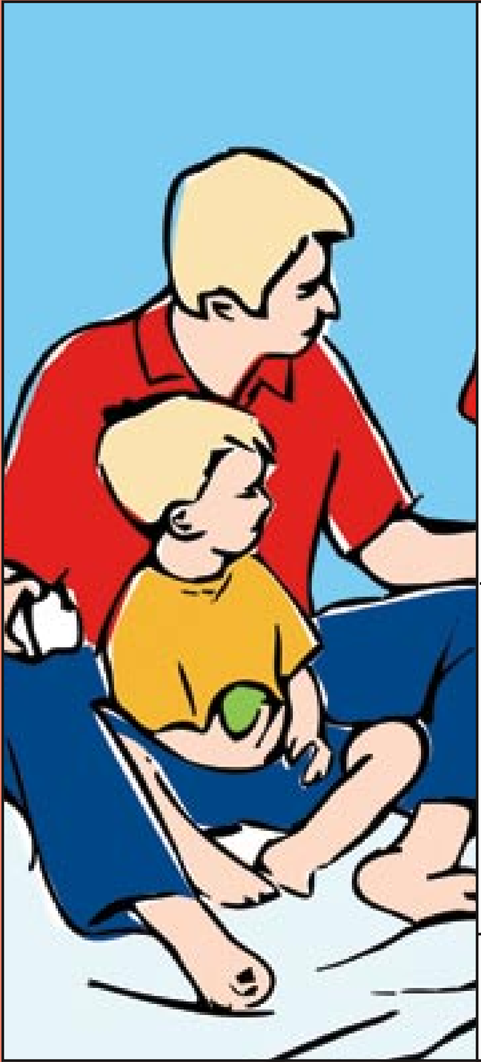

Segment Description

These guys are your standard Aussie country stock.

Less than 10% speak a language other than English at home and the proportion of those born in Australia is 15% above the national average.

Young families with parent's aged 40-49 and kids aged 10-14 are common. They're not interested in studying, with levels at TAFE and University well below national levels. If they do study, it's qualifications in agriculture, health and education that they're likely to walk away with.

Many homes are owned outright or being mortgaged with monthly repayments usually around $400 to $600. Household incomes are generally average across most bands, although those earning more than $75,000 per year are definitely under represented.

Many households have two to three cars.

Defining Features

1. Very few people speak a language other than English at home
2. Household incomes follow national averages (normally country areas have lower incomes)
3. 2 – 3 car households are common
4. Qualifications in agriculture, health and education are over represented
5. 50% more than the national average work at home

Key Expenditure

Diesel fuel
Removalist charges
Tyres & tubes
Bacon
Rice
Jewellery
Rail fares

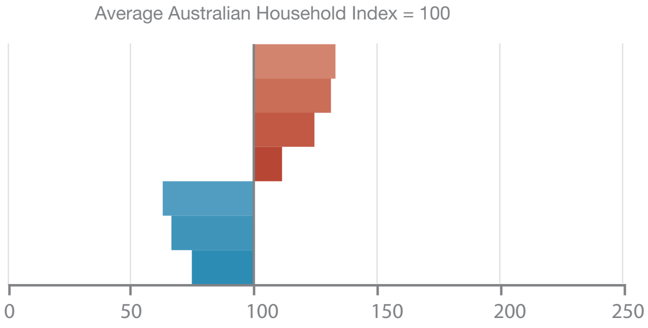

Population Profile

54% of the 380,588 people that live in this segment live in Inner Regional areas, 23% live in Outer Regional, and 16% live in Major Cities.

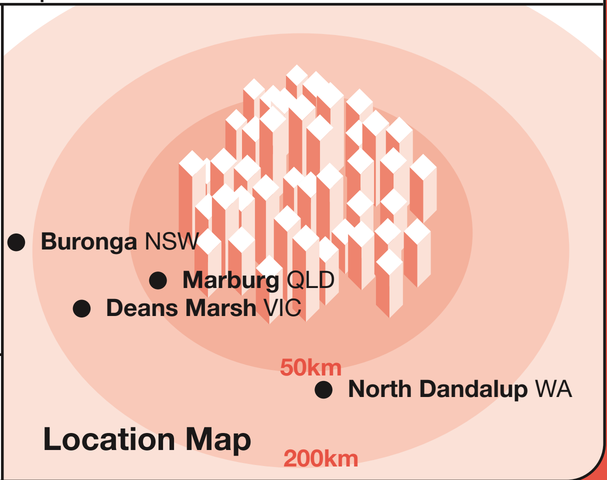

Location Map

Pathfinder Solutions © Copyright 2003 /// phone: +61-3-9670 3030 /// email: info@pathfindersolutions.com.au

PATHFINDER SEGMENTS OF AUSTRALIA

41 Dairy Farming

The cream of the crop

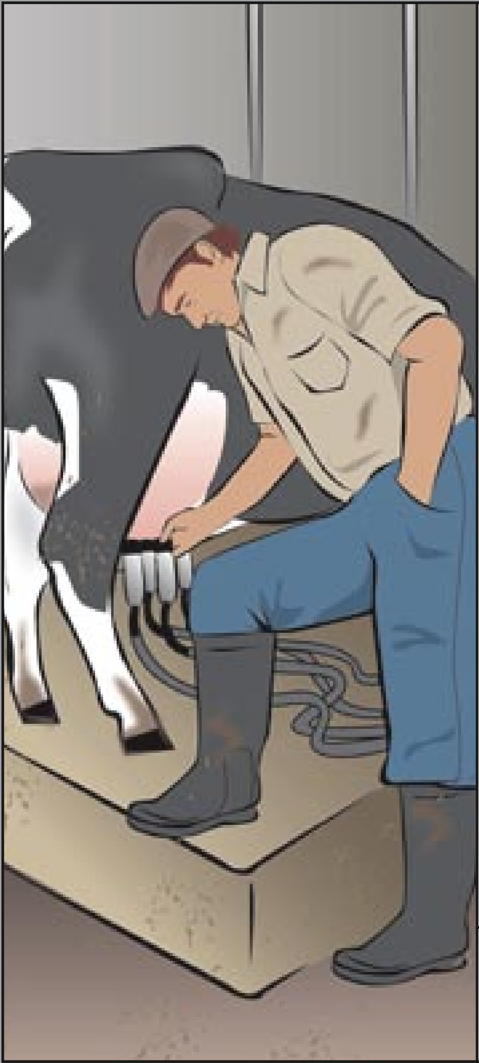

Segment Description

According to the agricultural census, this segment accounts for 35% of the total land area used for dairying in Australia, and 42% of all dairy farms can be found here.

These country folk are very unlikely to have been born out of Australia and are mostly employed in agriculture (40%).

Household income levels are average across most bands, although you are likely to find numbers of households with negligible or no income, as well as income in the $30,000 to $35,000 per annum bracket.

These are stable families with low rates of separation and divorce. Almost half the homes are owned outright and well over half the population were at the same address five years ago.

There are lots of kids aged 10 to 14 and adults aged 55 to 59. Most of the kids attend government run primary and secondary schools. Most adults completed Year 11 or an earlier level of schooling.

Over a quarter of homes have 3 or more vehicles.

Commercial and new home development has been on the rise since 1995.

Defining Features

1. Around 40% of the labour force is employed in agriculture
2. Weekly household income is skewed towards the $31,000 - $36,000 p.a. range
3. Ages 10-14 and 55-59 are over represented.
4. Most children attend government run primary and secondary schools.
5. This segment has higher proportions of people born in Australia, and 92% of homes speaks English only
6. Of those paying mortgages, 36% are below $600 per month

Key Expenditure

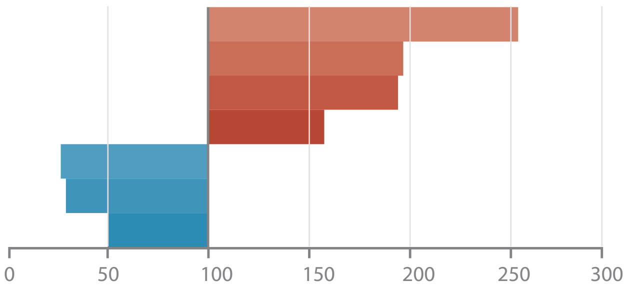

Population Profile

82% of the 101,710 people that live in this segment live in Inner Regional areas, and 18% live in Outer Regional areas.

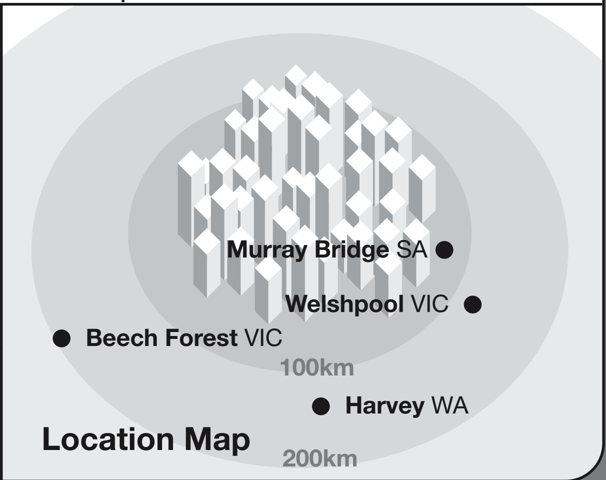

Location Map

Pathfinder Solutions © Copyright 2003 /// phone: +61-3-9670 3030 /// email: info@pathfindersolutions.com.au

PATHFINDER SEGMENTS OF AUSTRALIA

42Regional Mix

Country blend

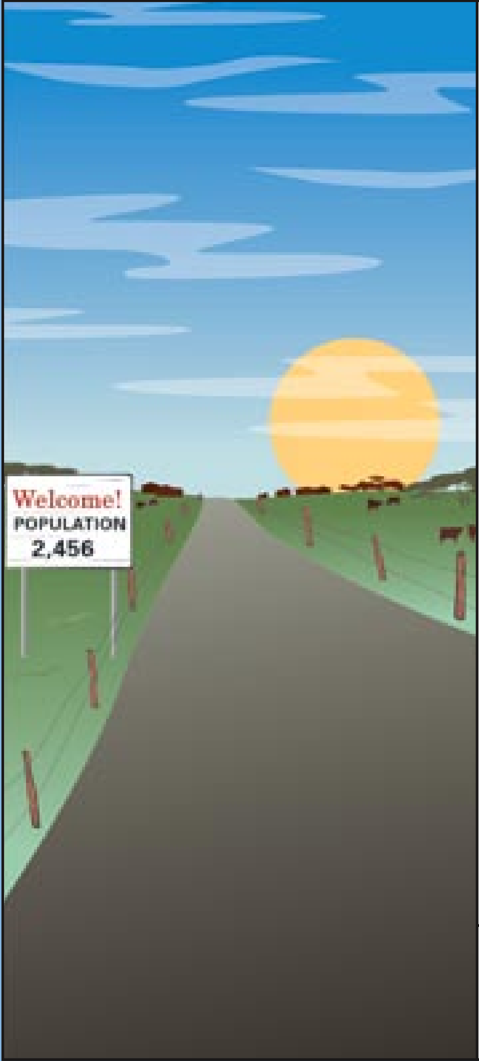

Segment Description

These mostly regional areas contain a mixture of tradespeople, intermediate skilled workers and labourers, with an over representation of Aboriginal and Torres Straight Islanders.

Household income tends to be average across most income bands, although unemployment rates are 24% above average.

The financial extremes of this group flattened out between 1991 and 2001 with the lowest income bracket declining by 9% and the highest declining by 28%.

Land values are relatively low. Spending is high on fuels for heating, and TV aerials. There have been steady levels of commercial building and home extensions over the past 10 years, with reduced levels of home building since 1996.

There is strong interest in pay TV, country music and fishing.

Defining Features

1. This segment is found predominantly in regional areas
2. A mixture of tradespeople, intermediate skilled workers and labourers predominate
3. Unemployment rates are 24% above the national average
4. Land values are relatively low
5. Although Indigenous people are over represented, they still only account for 3% of the population here
6. Young adults and the very elderly are under represented

Key Expenditure

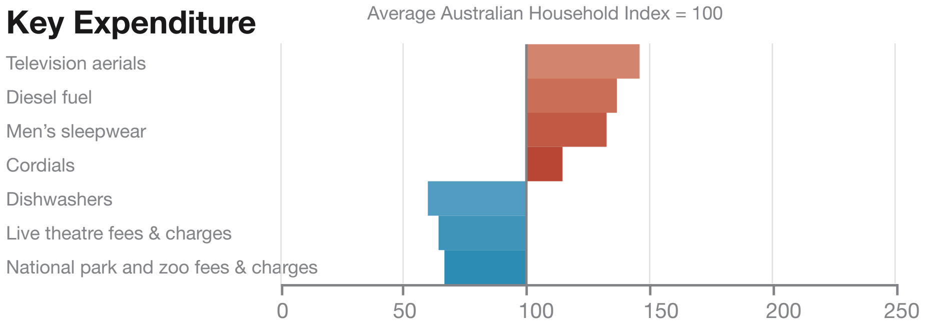

Population Profile

52% of the 204,020 people that live in this segment live in Inner Regional areas, 32% live in Outer Regional, and 14% live in Major Cities.

Location Map

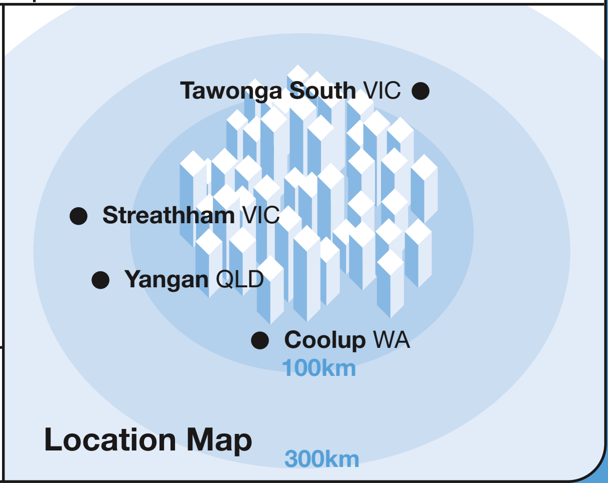

Pathfinder Solutions © Copyright 2003 /// phone: +61-3-9670 3030 /// email: info@pathfindersolutions.com.au

PATHFINDER SEGMENTS OF AUSTRALIA

43 Young Regional Families

Aussie battlers

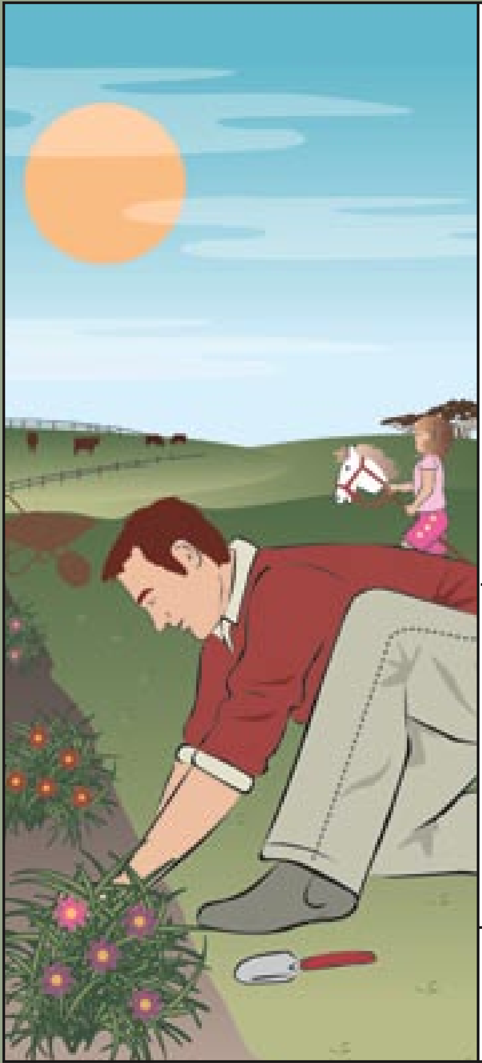

Segment Description

These predominantly regional families are a mix of both single parents and couples with kids generally under 15. It's not a highly multicultural segment with less than 3% of people speaking a language other than English at home.

Education levels are low, with many people having dropped out of school at Years 8, 9 or 10.

Household income tends to be average across all income bands. Most mortgage repayments are less than $800 per month and weekly rents are usually around $50 to $149.

Most of the men are employed in areas such as agriculture, mining and utilities while the women generally work in retail and hospitality.

Home building activity was strong from 1987-1991 but has since tapered off, save for the construction of garages.

Pay TV, crafts and sewing, country music and gardening are all strong interests here.

Defining Features

1. Weekly household incomes are strongest in the $15,000 - $41,000 p.a. range
2. 45% of households are made up of families with children under 15
3. Key occupational groups include tradespeople, intermediate skilled workers and labourers
4. Building activity on houses was strong from 1987-1991 but has tapered off since then
5. Less than 3% of people speak a language other than English

Key Expenditure

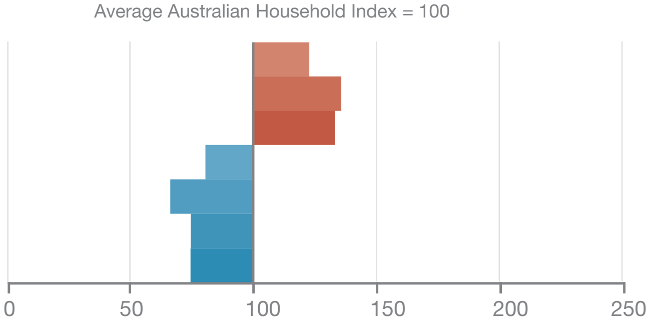

Population Profile

65% of the 296,782 people living in this segment live in Inner Regional areas, with only 24% living in Outer Regional areas.

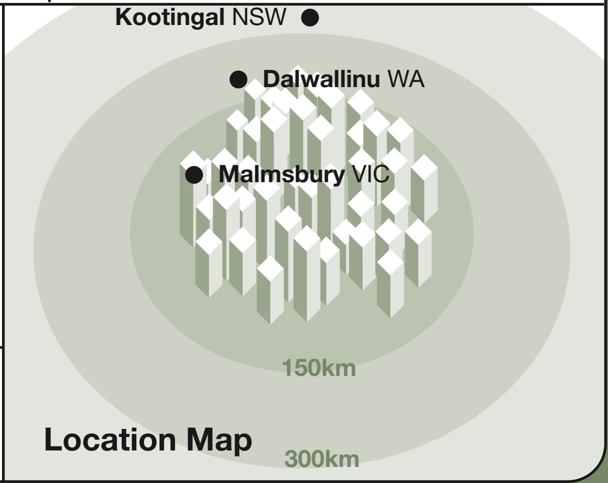

Location Map

Pathfinder Solutions © Copyright 2003 /// phone: +61-3-9670 3030 /// email: info@pathfindersolutions.com.au

PATHFINDER SEGMENTS OF AUSTRALIA

44 Aging Regional Towns

Retirement central

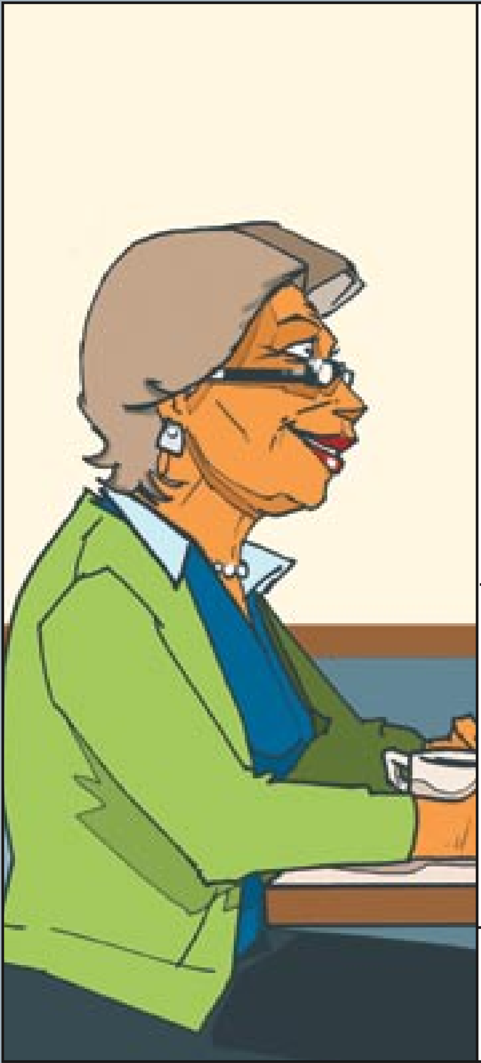

Segment Description

As a result of an aging population and residents reaching retirement age, these towns experienced a 30% decline in the top income bracket between 1991 and 2001.

Many people are over 60 years of age in these mainly regional towns, the proportion of people over 65 increasing by 43% from 1991 to 2001. Around 10% are widowed, and many live alone.

Not many homes are rented or mortgaged – most are owned outright by this stage.

As you might expect, unemployment is 22% above the national average. If working, the most common jobs are in agriculture, mining, utilities and hospitality. There are also many who work in home based businesses.

Building activity levels have been low for the past 15 years, except for a burst of dual occupancy developments between 1989 and 1991.

These people love lawn bowls, Pay TV, grandchildren, charity work, fishing and folk music.

Defining Features

1. All ages over 60 years are over represented, with the proportion of people over 65 increasing by 43% from 1991 to 2001
2. Around 10% of the people are widowed and there are many elderly people living alone
3. Unemployment is 22% above the national average
4. The majority of incomes are less than $26,000 p.a.
5. There are low levels of Internet use in this segment

Key Expenditure

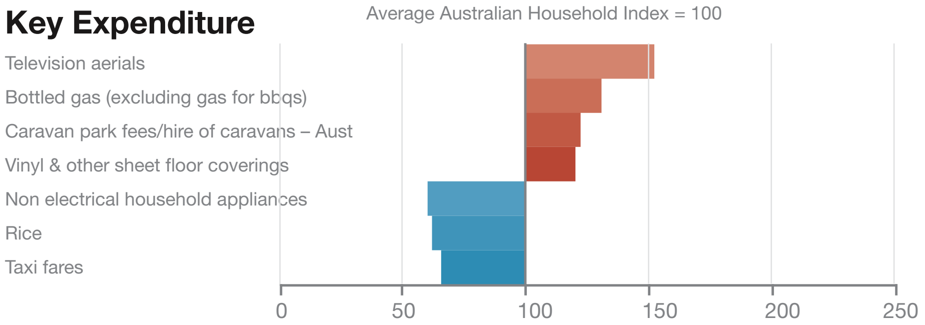

Population Profile

63% of the 522,066 people that live in this segment live in Inner Regional areas, 29% live in Outer Regional, and 4% live in Major Cities.

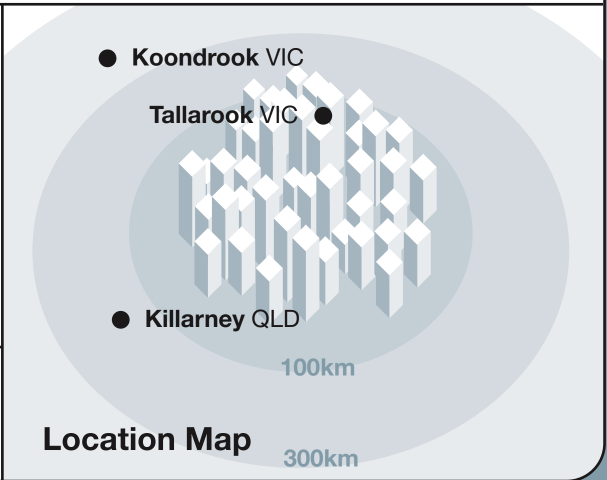

Location Map

Pathfinder Solutions © Copyright 2003 /// phone: +61-3-9670 3030 /// email: info@pathfindersolutions.com.au

PATHFINDER SEGMENTS OF AUSTRALIA

45 Struggling Country Towns

Battling on the outer

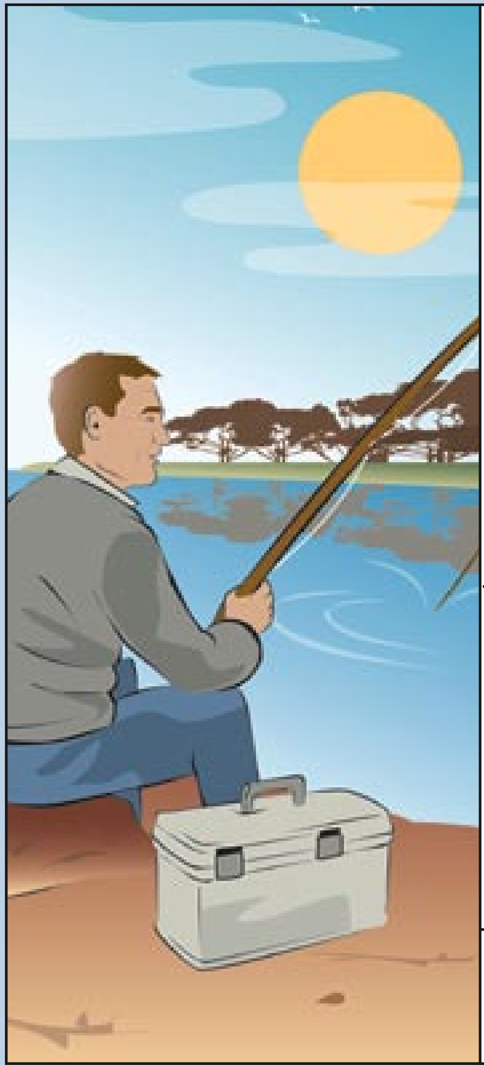

Segment Description

A reflection of the hardship experienced in declining regional towns is the fact that between 1991 and 2001 the top income level declined by 45% and the lowest income quartiles grew around 14%.

Unemployment is rampant at 44% above national rates and there are relatively few professionals, associate professionals or advanced clerical workers. Household incomes of less than $35,000 per year are evident here, with higher incomes of $75,000 plus being vastly under represented.

Internet usage is low and many drop out of school at Years 8, 9 and 10.

Single parent families have risen 46% since 1991, with the proportion of single-parent families being 24% above national rates. Around 26% of people live alone.

There are very few who follow a religion, but those who follow the Christian faith are more likely to be Anglican.

Pay TV, country music, lawn bowls, shooting and fishing are popular pastimes here.

Defining Features

1. Unemployment is 44% above the national average and the proportion of single parent families has increased by 46% from 1991
2. Employment is likely to be within the mining, electricity, gas and water industries, and a high proportion of the females work within retail
3. Household incomes under $41,600 p.a are over represented
4. Education levels past year 10 are uncommon and Internet usage is low

Key Expenditure

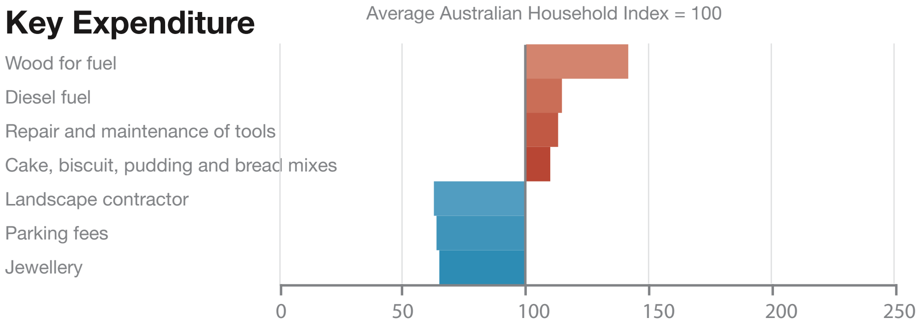

Population Profile

66% of the 345,407 people that live in this segment live in Inner Regional areas, 23% live in Outer Regional, and 9% live in Major Cities.

Location Map

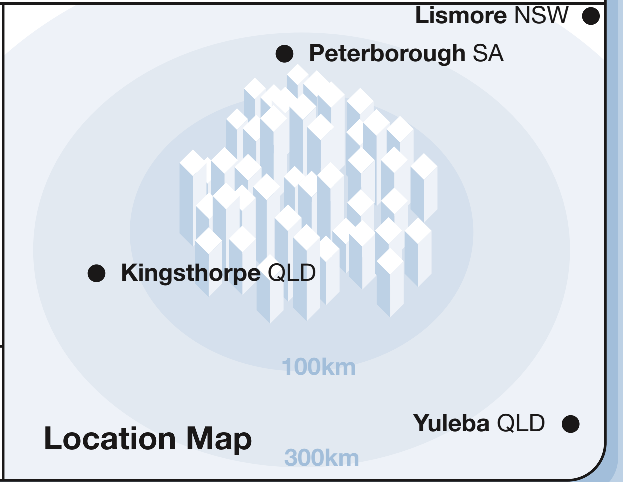

Pathfinder Solutions © Copyright 2003 /// phone: +61-3-9670 3030 /// email: info@pathfindersolutions.com.au

PATHFINDER SEGMENTS OF AUSTRALIA

46 Forestry, Fishing & Farming

The great outdoors

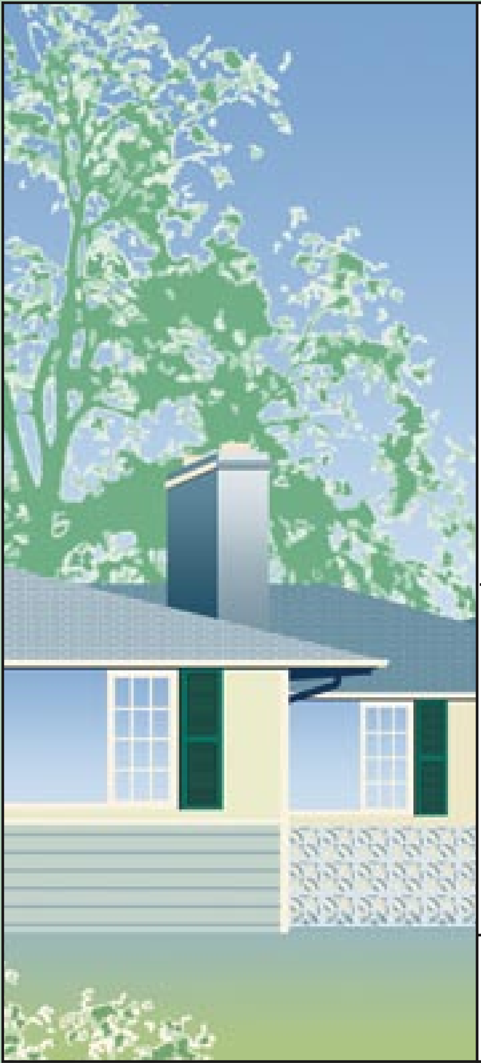

Segment Description

This group love the fresh air with around a quarter being employed in forestry, fishing or farming - around 15% working from home.

These areas are quite underpopulated and isolated with 16% of homes being unoccupied and mail order purchasing being a common way to buy goods. Commercial building has been increasing here since 1994.

Many homes are owned outright and yearly household incomes of $15,000 to $35,000 are over represented when compared with the average across all segments.

Married couples and couples with kids are the norm. There are lots of kids aged 10 to 14 and adults aged between 50 and 64. Many drop out of school before Year 10.

They're right in to country music with over 40% listing it as a favourite.

Defining Features

1. 25% of the workforce in this segment are employed in forestry, fishing or farming
2. Ages 10-14 and 50-64 are over represented and education levels below Year 10 are over represented
3. Married couples and couples with children are over represented
4. Household incomes between $15,600 and $36,400 p.a. are over represented

Key Expenditure

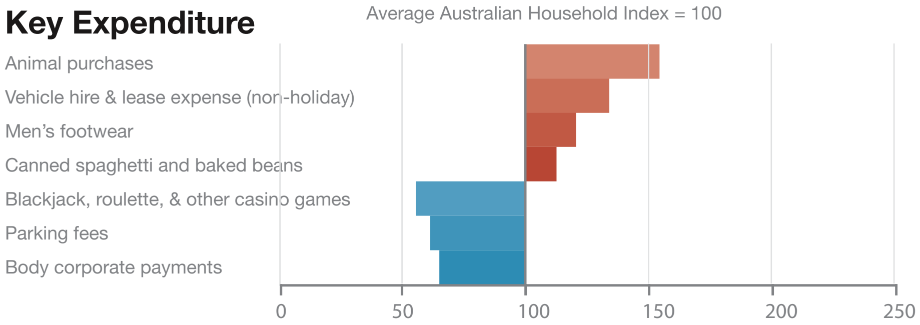

Population Profile

49% of the 147,449 people that live in this segment live in Inner Regional areas, 48% live in Outer Regional, and 3% live in Remote areas.

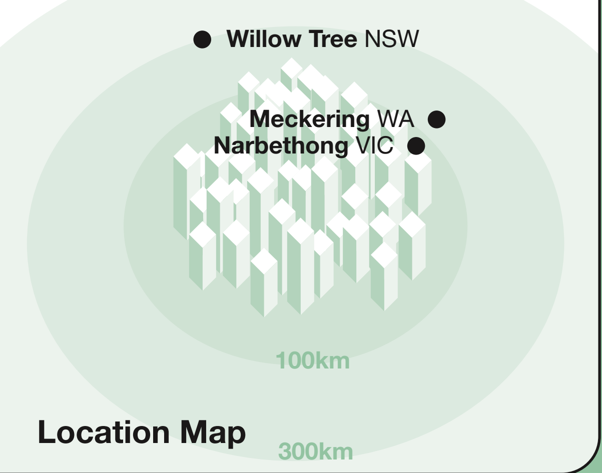

Location Map

PATHFINDER
SEGMENTS
OF AUSTRALIA

47Regional Battlers

Grass roots living

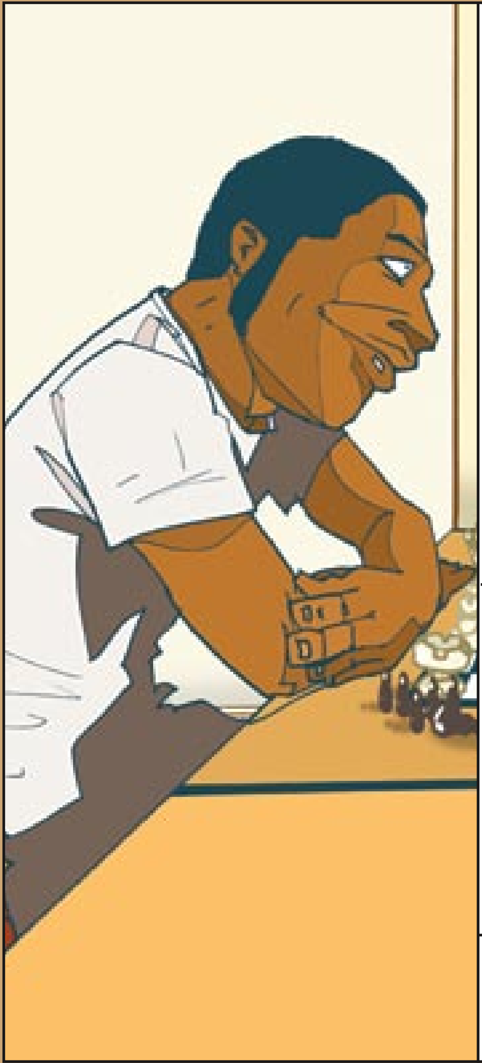

Segment Description

This is basic, country living where most are battling to get by.

Mainly comprised of people aged over 60, many in this segment are separated, divorced and widowed and there are many single parent families and people who live alone.

Few own a car and unemployment is rife at 44% above the national average. Yearly household incomes up to $35,000 are over represented when compared with the average across all segments, with incomes above $50,000 being vastly under represented.

Most people finished school at Year 10 or earlier and are employed as labourers or have intermediate roles in agriculture or transport.

In these areas there has been little activity in home building or extensions in the past 15 years; however there have been high levels of commercial development.

People in these areas get into Pay TV, lawn bowls, gardening and country music.

Defining Features

1. All age groups over 60 years are over represented and there are also high levels of separated, divorced and widowed people as well as one parent families and lone person households
2. All household income levels under $31,200 p.a. are over represented
3. There are low education levels with most people finishing school before Year 10
4. A high proportion of households do not have a car

Key Expenditure

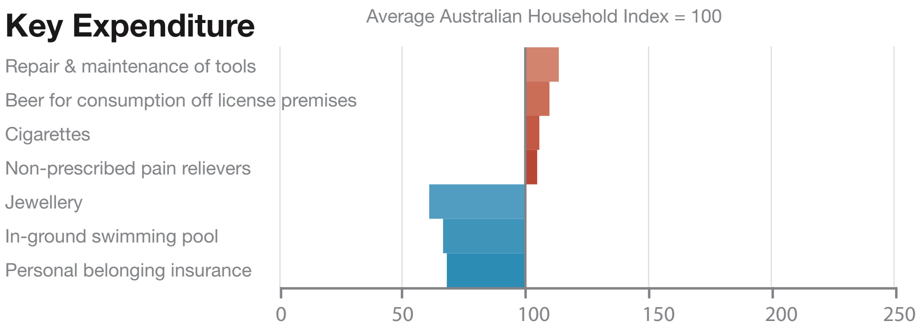

Population Profile

69% of the 81,057 people that live in this segment live in Inner Regional areas, 24% live in Outer Regional, and approx 3% live in Remote areas.

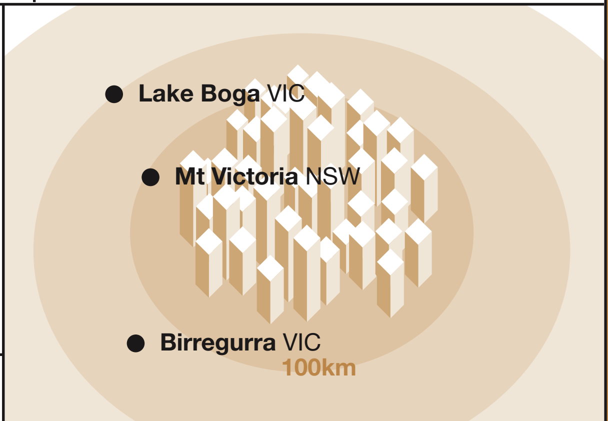

Location Map

Pathfinder Solutions © Copyright 2003 /// phone: +61-3-9670 3030 /// email: info@pathfindersolutions.com.au

PATHFINDER SEGMENTS OF AUSTRALIA

48 Low Income & Aging Coastal

Holiday sleepy hollow

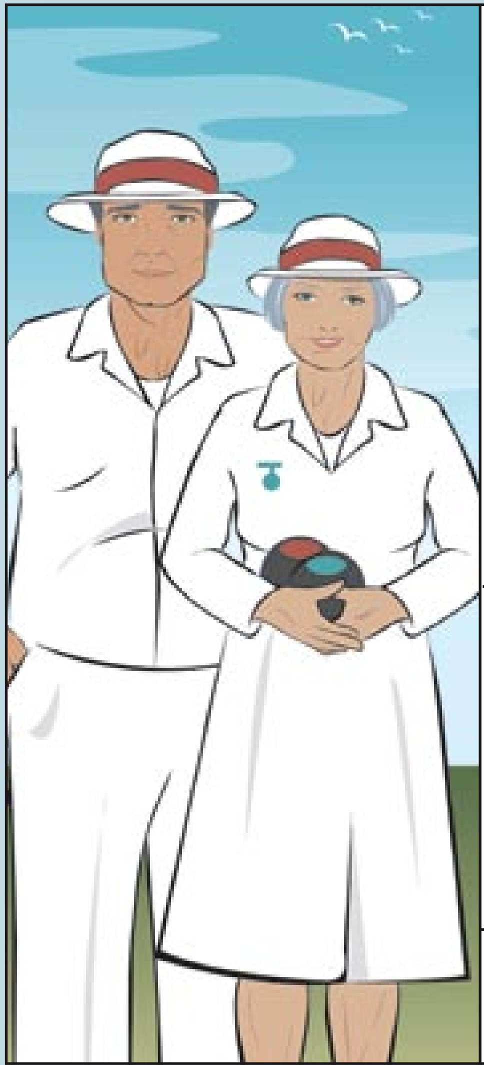

Segment Description

These are your quiet areas – mainly holiday towns which have several houses unoccupied at certain times during the year.

The population is predominantly older people with over a quarter being over 65. Around 10% are widowed. It's a slower lifestyle here and there's not much interest in computers or the Internet.

Yearly household incomes up to $25,000 are over represented when compared with the average across all segments. Household incomes above $50,000 are vastly under represented here.

There is high unemployment; however those who are working are mainly employed in construction or hospitality. This segment has the highest proportion of home-based trade businesses.

People spend time watching television and talking on public phones.

Defining Features

1. 25% of the people living within this segment are over 65 years and 10% are widowed
2. Ages from 0-35 are under represented
3. This segment is over represented in living in caravans and cabins, and rent payments on all dwellings are low
4. Most households have incomes between $10,400 and $36,400 p.a.

Key Expenditure

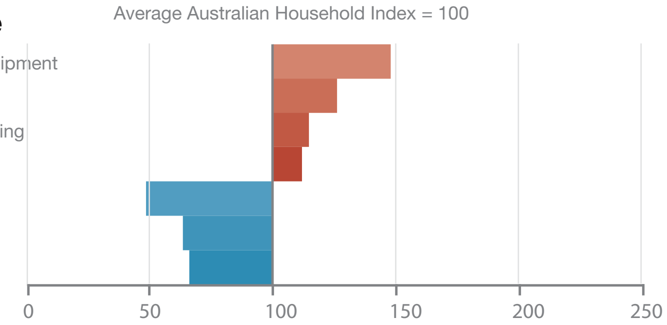

Population Profile

44% of the 539,385 people that live in this segment live in Inner Regional areas, 41% live in Major Cities and 14% live in Outer Regional areas.

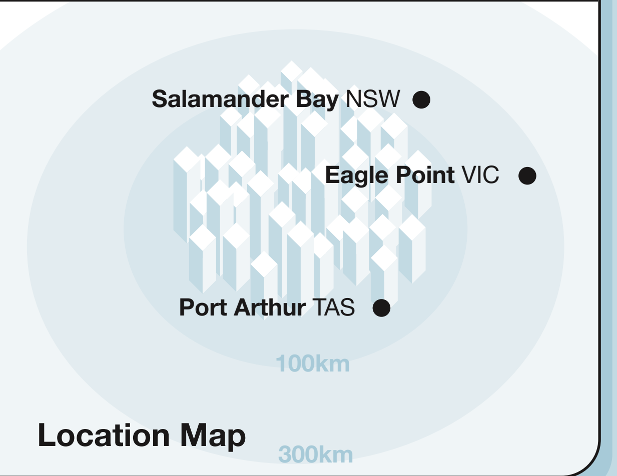

Location Map

Pathfinder Solutions © Copyright 2003 /// phone: +61-3-9670 3030 /// email: info@pathfindersolutions.com.au

PATHFINDER SEGMENTS OF AUSTRALIA

49 Vineyards & Farmlets

Grapes & grazing

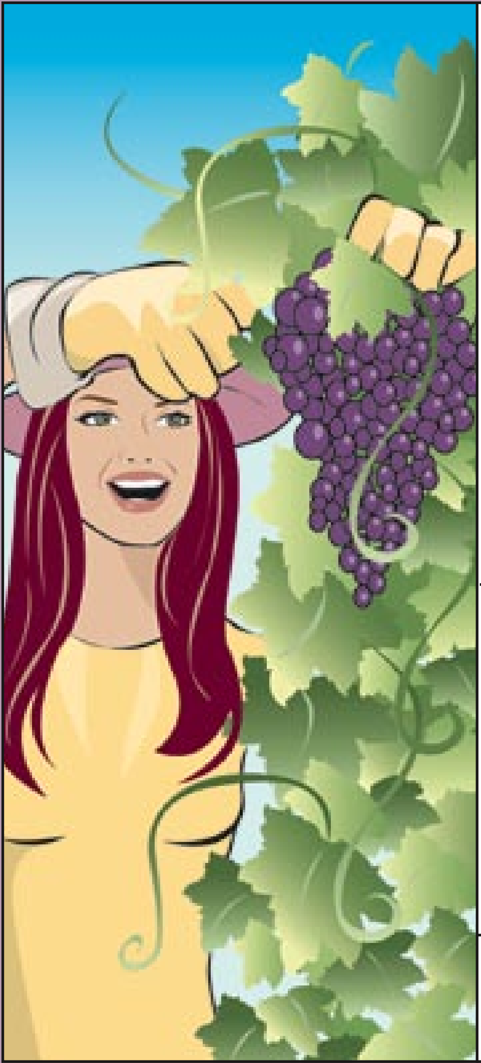

Segment Description

Here we're talking families on farms. According to the agricultural census, 20% of all grape growing properties can be found in this segment.

There is very low unemployment and around 40% of people work in agriculture. That's not just manual labour, as farm managers and administrators are also common. Household income is average across most income bands with the exception of the $30,000 to $35,000 per year income band which is slightly over represented when compared with the average across all segments.

Most are two parent families, with single parent families making up less than 10%. The most popular age group is 50 to 59 and most people were born in Australia (around 80%, which is 10% above average). Around 6% of the population in this segment speak Greek or Italian at home.

This small segment loves the outdoors and travel and has the highest rate of subscription to gardening magazines.

Since the late 80s there have been significant levels of commercial development in these areas, running at more than 3 times the Australian average.

Defining Features

1. People aged between 50-59 are over represented
2. Around 40% of the workforce are in agricultural and household incomes of $31,200-$36,400 are over represented
3. 80% of people were born in Australia and around 6% of the people within this segment speak Greek or Italian at home
4. The average size of a farm in this segment is 570 hectares

Key Expenditure

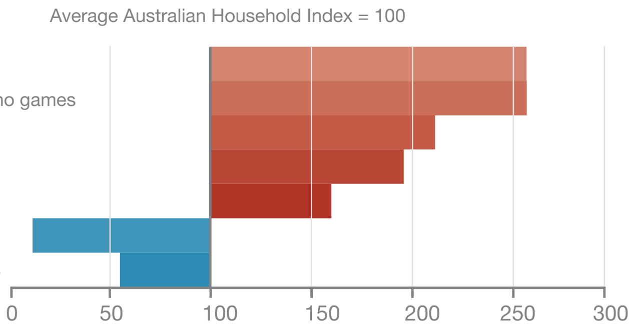

Population Profile

55% of the 28,666 people that live in this segment live in Outer Regional areas, and 45% live in Inner Regional areas.

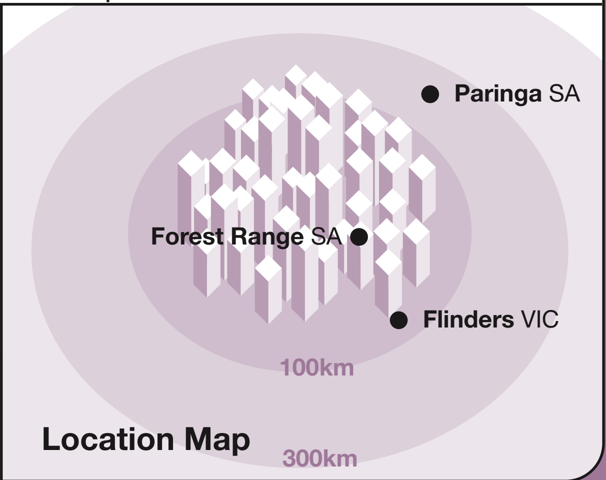

Location Map

Pathfinder Solutions © Copyright 2003 /// phone: +61-3-9670 3030 /// email: info@pathfindersolutions.com.au

PATHFINDER SEGMENTS OF AUSTRALIA

50 Mixed Farming Areas

Regional diversity

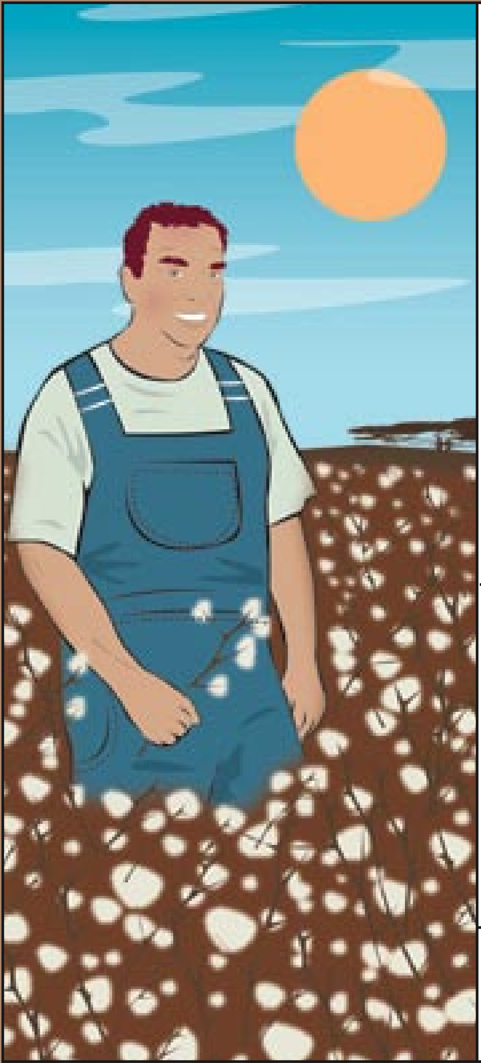

Segment Description

This is a farming segment with 60% of the male workforce being employed in agriculture and more than a quarter working on their own properties. According to the agricultural census, this segment accounts for 53% of the total land area used for cotton farming, and 44% of the total land area used for pig farming in Australia. 36% of all cotton farms across Australia can be found here.

There is hardly any unemployment and over half of homes are owned outright with mortgages being usually less than $600 per month. Yearly incomes are generally from $15,000 to $35,000 per household.

We're talking lots of big, happy families, often of five people or more, with little divorce or separation. Not many (less than 6%) speak a language other than English at home. They're a church-going bunch with Anglican, Lutheran and Presbyterian denominations strongly supported.

Kids are generally sent to government schools for both primary and secondary education and most people have left school by the end of Year 11. Internet usage is low. Three or more vehicles are common.

There was a burst in commercial development here from 1996 to 2000 but apart from that, building activity has been limited. These folk love lawn bowls, gardening and golf. Over 50% love country music.

Defining Features

1. People aged between 5-14 and 55-64 are over represented
2. Couple families with children are common and there are low rates of separation and divorce
3. This segment is also over represented in people born in Australia
4. 60% of the male workforce are employed in agricultural and unemployment is low

Key Expenditure

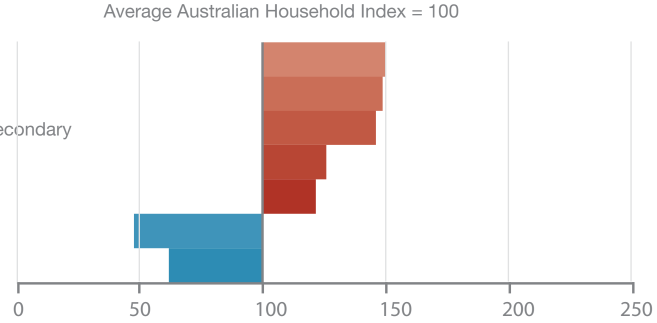

Population Profile

62% of the 202,009 people that live in this segment live in Outer Regional areas, 26% live in Inner Regional areas and 11% live in Remote areas.

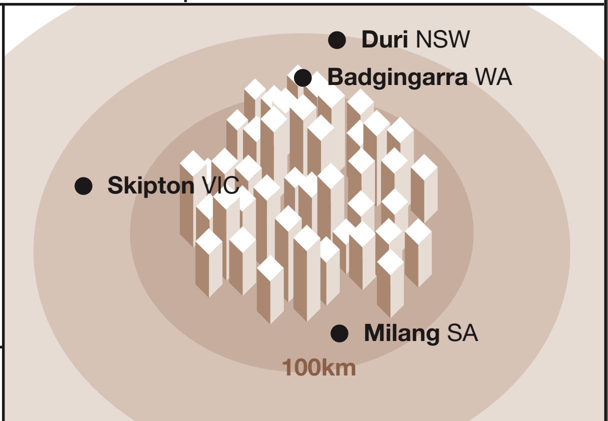

Location Map

Pathfinder Solutions © Copyright 2003 /// phone: +61-3-9670 3030 /// email: info@pathfindersolutions.com.au

PATHFINDER
SEGMENTS
OF AUSTRALIA

51 Wheat Farming

Bread winners

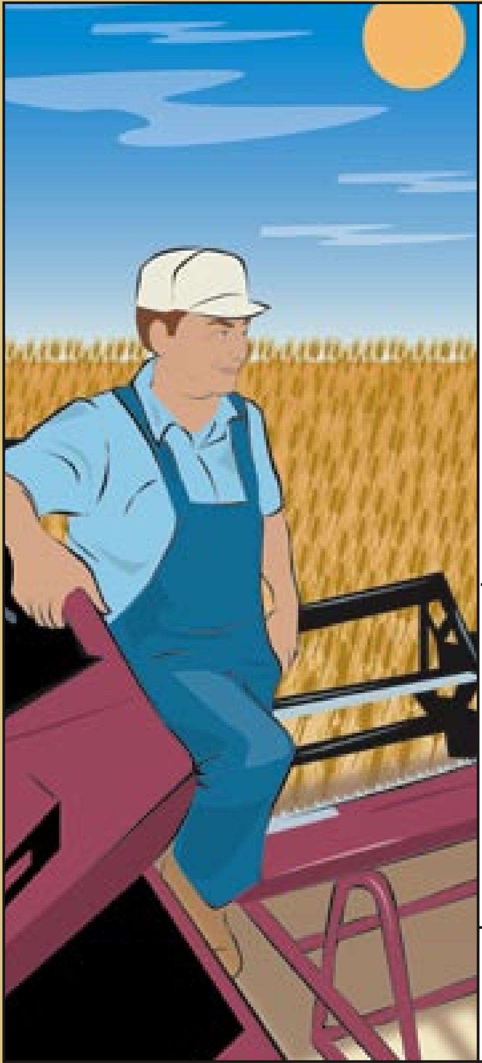

Segment Description

With all that wheat around, naturally these are strong farming areas. According to the agricultural census, this segment accounts for 69% of the total land area used for wheat farming and 51% of the total land area used for poultry farming.

This segment contains 64% of all Australian wheat farms. Those in these regional and remote areas are mainly employed in agriculture (over 60% of males) as farm managers, administrators and labourers.

Most were born in Australia, speak English at home and are married couple families. They like to settle down in one spot and more than 70% have lived in the same place for five years or more. Most people are aged between 5 and 14 or 55 to 64.

Yearly household incomes between $20,000 and $35,000 are over represented when compared across all other segments. About a third of households have three or more vehicles.

Most kids go to government schools (over 80%) and don't continue beyond Year 11. Unemployment is low.

Mail order purchasing is popular out here and these country folk love Pay TV, gardening, country music, lawn bowls and charity work. There is also strong interest in football, fishing, and golf.

Defining Features

1. A high proportion of the people were born in Australia
2. This segment is stable, with 70% of people having lived in the same house for more than 5 years
3. Married couples are over represented, as are household incomes between $20,800 - $36,400 p.a.
4. 60% of males are employed in agriculture with a large number of farm managers, administrators and labourers.

Key Expenditure

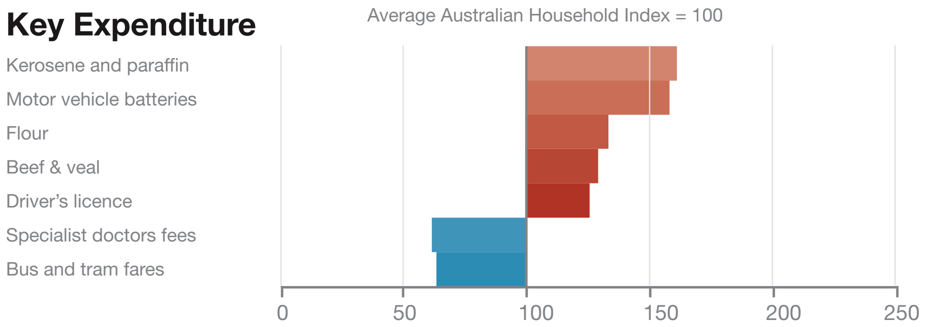

Population Profile

62% of the 202,009 people that live in this segment live in Outer Regional areas, 26% live in Inner Regional areas and 11% live in Remote areas.

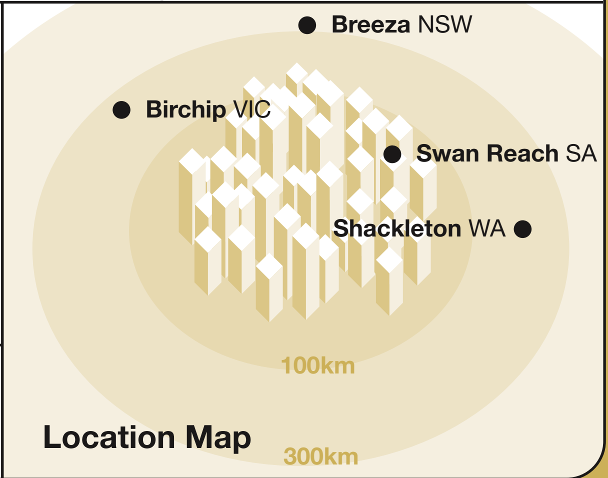

Location Map

Pathfinder Solutions © Copyright 2003 /// phone: +61-3-9670 3030 /// email: info@pathfindersolutions.com.au

PATHFINDER SEGMENTS OF AUSTRALIA

52Sheep Runs

Woolly jumpers

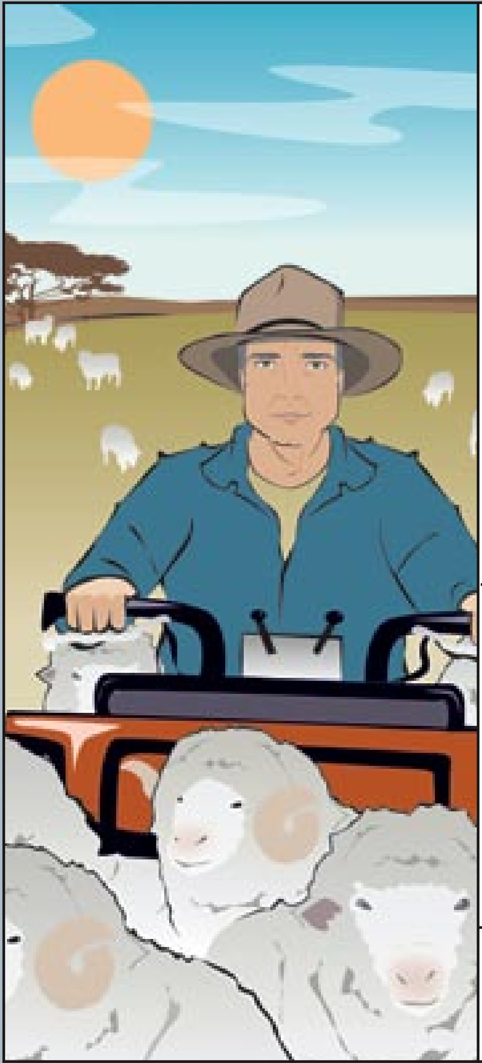

Segment Description

These families live and breathe farming, and around 40% are employed in agriculture. According to the agricultural census, this segment accounts for 42% of the total land area used for sheep farming across Australia. 30% of all sheep farms are found here.

They really have no interest in the Internet and the most common age group is 50 to 69. They're generally out of school by the end of Year 10.

Mainly married couples with kids, and households with five of more members are common. Households generally earn up to $35,000 per year and if renting, they pay less than $150 per week. They drive trucks, motorbikes or walk to work and often own three or four dogs and a number of households (around 8%) have four or more cats!

Around 20% of houses were unoccupied at the time of the Census and there has been very limited building activity of new homes, extensions or other improvements over the past 15 years.

Defining Features

1. Households with more than 5 people are over represented, as are married couples with children
2. Ages 50-69 are over represented and this segment has low levels of education with people finishing at Year 10 or below common
3. Household incomes under $15,600 p.a. and incomes between $15,600-$36,400 p.a. are over represented
4. Rental prices are low and there has been limited building activity over the past 15 years
5. More than 20% of existing dwellings were unoccupied at the time of the Census

Key Expenditure

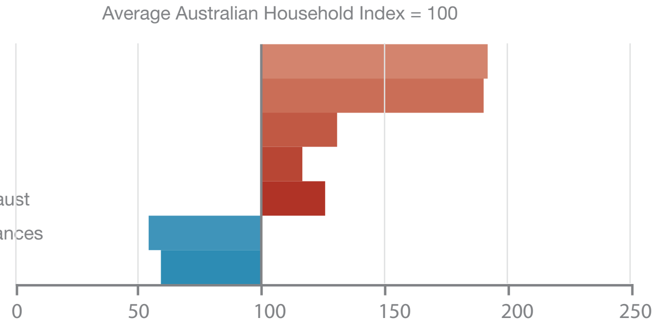

Population Profile

55% of the 85,465 people that live in this segment live in Outer Regional areas, 31% live in Inner Regional areas, 9% live in Remote areas and 5% in Very Remote areas.

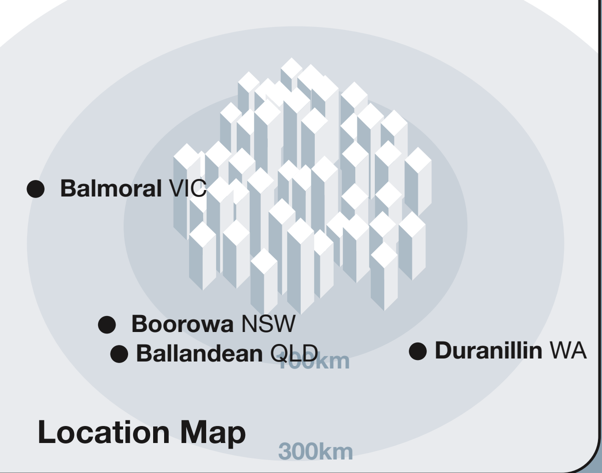

Location Map

Pathfinder Solutions © Copyright 2003 /// phone: +61-3-9670 3030 /// email: info@pathfindersolutions.com.au

PATHFINDER SEGMENTS OF AUSTRALIA

53Market Gardens

Quiet life

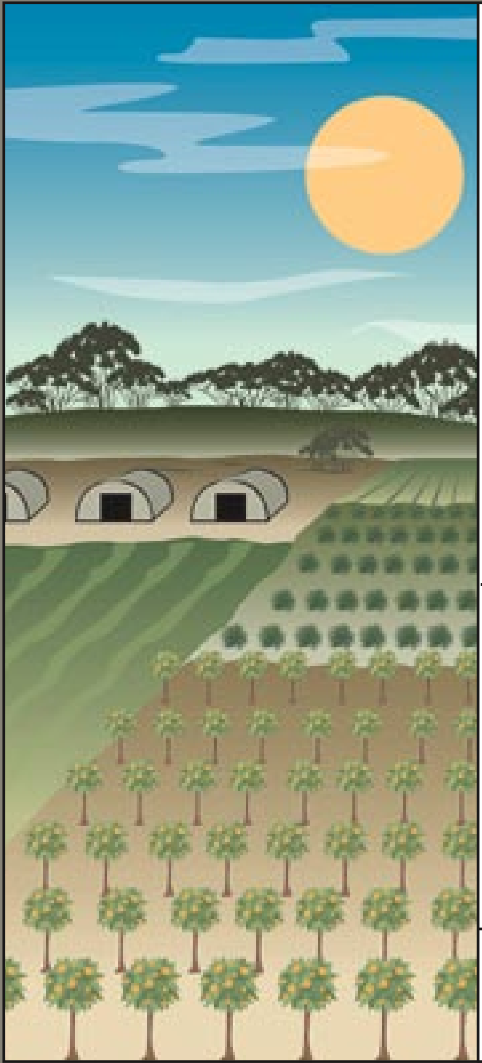

Segment Description

This small regional group comprises those aged 55 to 74 years and around 12% live in caravan parks (possibly due to the transient nature of the people working in and around market gardens). Around 10% are visitors to these areas.

As noted above, the predominant agricultural activity in this sector is market gardening, which represents 5% of total land area used for market gardening across Australia. Around 7% of all market gardens are found here.

Many didn't go to school and if they did, it's likely they dropped out after Year 10 or earlier. Around 40% work in agriculture.

Households generally earn up to $35,000 per year.

Pay TV, horse riding and fishing are popular activities with this segment. Country music and Rugby League are also very popular.

Defining Features

1. 40% of this segment work in the agricultural industry
2. Household incomes between $10,400 - $36,400 p.a. are over represented
3. Indigenous Australians are over represented and 12% of people living in this segment live in a caravan
4. Education levels are low with over representation of people leaving school at the end of Year 10 or earlier
5. 10% of this segments' population are visitors

Key Expenditure

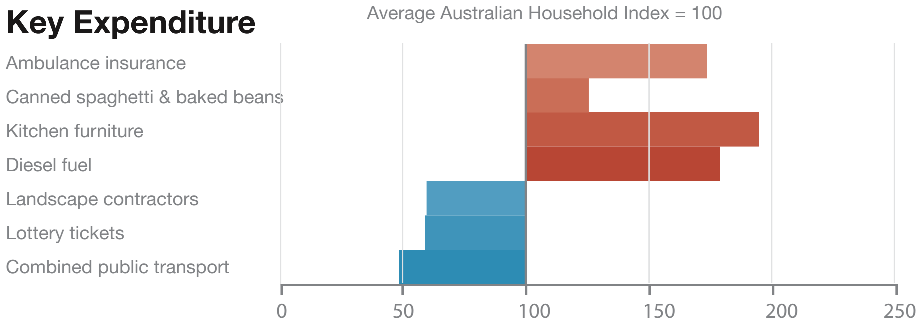

Population Profile

53% of the 34,376 people that live in this segment live in Outer Regional areas, 37% live in Inner Regional areas, 6% live in Remote areas and 4% live in Major Cities.

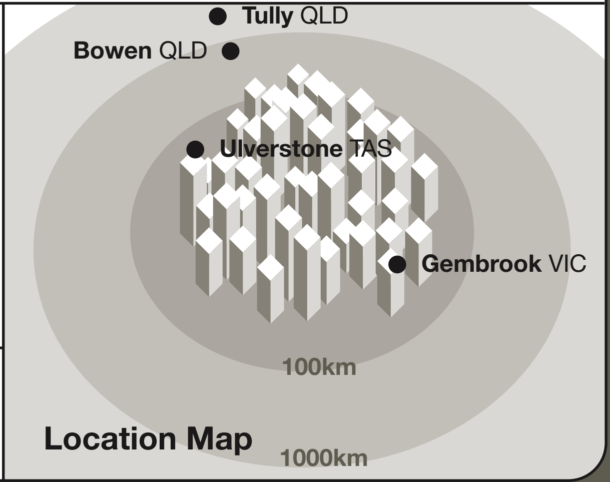

Location Map

Pathfinder Solutions © Copyright 2003 /// phone: +61-3-9670 3030 /// email: info@pathfindersolutions.com.au

PATHFINDER SEGMENTS OF AUSTRALIA

54 Sugar Cane Plantations

Life is sweet

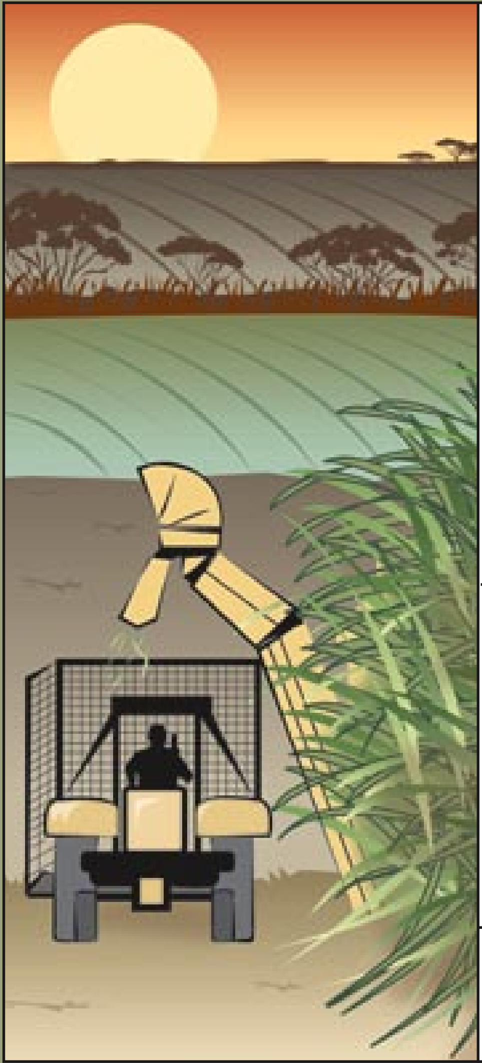

Segment Description

This hot-weather segment is mainly regional, though also comprising some remote areas. According to the agricultural census, this segment accounts for 55% of the total land area used for sugar cane farming across Australia. 55% of all sugar cane farms are also found here.

Lots of people reside in sleep-outs and improvised housing with many having lived in the same place for five or more years.

Around 36% work in agriculture and left school at Year 10 or before. Household incomes between $15,000 and $35,000 per year are over represented. Use of credit unions for banking is at double the national average.

The most popular age groups are from 10 to 14 and 55 to 64 and they are a Christian bunch with above average levels of Catholic and Uniting Church affiliations.

Over the past 10 years there have been significant levels of commercial development, building extensions to homes and home construction.

People out here love adventure sports such as shooting, fishing and motoring. They also dig country music, horse riding and wildlife.

Defining Features

1. Over represented in age groups 10-14 and 55-64, and education to Year 10 level or lower is common
2. There was a burst in building extensions to homes between 1992 and 1999
3. Household incomes between $15,500 - $31,200 are over represented and there is a presence of households with no income
4. Low Internet use

Key Expenditure

Average Australian Household Index = 100

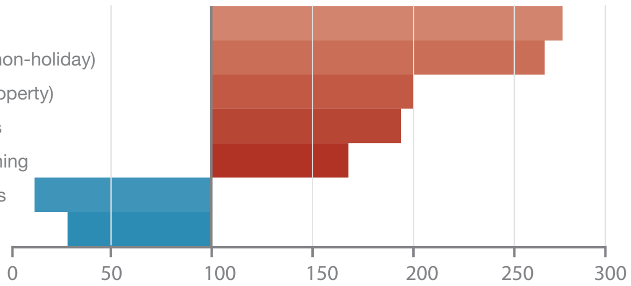

Population Profile

77% of the 49,756 people that live in this segment live in Outer Regional areas, 14% live in Inner Regional areas, and 8% live in Remote areas.

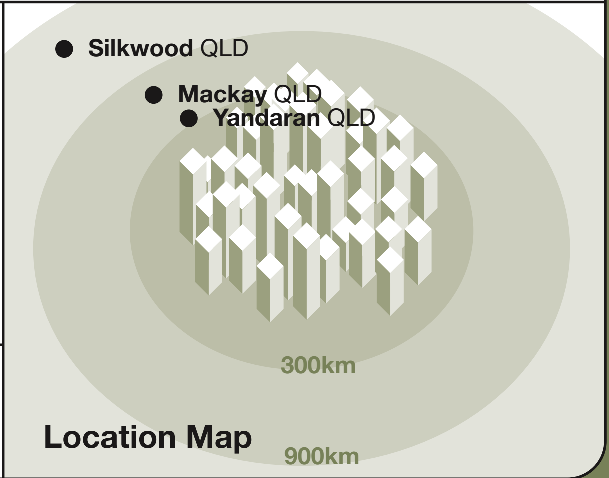

Location Map

Pathfinder Solutions © Copyright 2003 /// phone: +61-3-9670 3030 /// email: info@pathfindersolutions.com.au

PATHFINDER SEGMENTS OF AUSTRALIA

55Cattle Country

Grazing and lazing

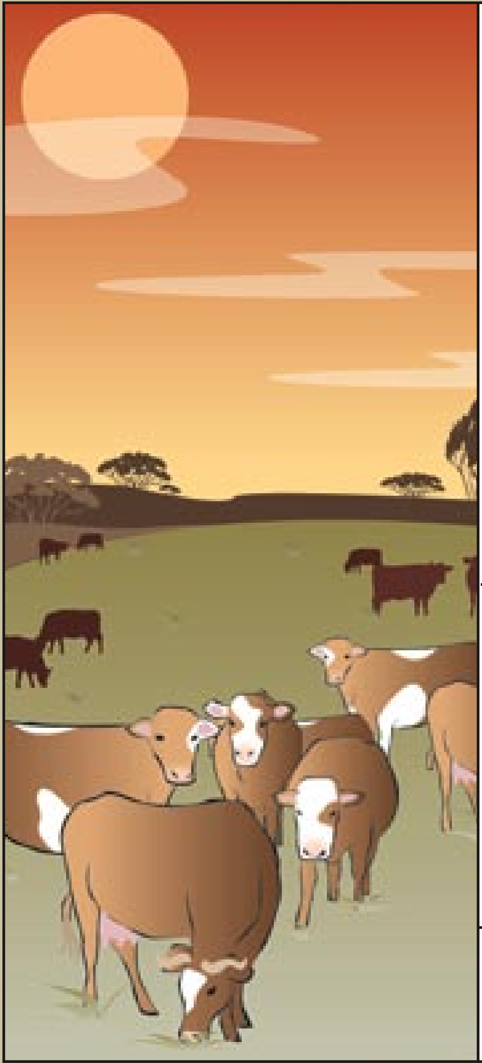

Segment Description

Most of these folk were born in Australia and it's pretty unusual that they speak a language other than English at home. According to the agricultural census, this segment accounts for 27% of the total land area used for cattle farming across Australia. Nearly 28% of all cattle farms are found here.

Around 40% work in agriculture with over 20% working on their own properties.

Household income levels are strongest up to $35,000 per year. There are not many singles, group households or single parent families here. Most families are couples with three or more kids. Those aged between 5 and 14 and 55 and 64 are over represented.

Most kids go to a government primary or secondary school.

Approximately 17% of homes in these areas are unoccupied. Over the past 15 years there have been consistently high levels of commercial building in these areas.

Pay TV, horse riding, fishing and interest in wildlife, gardening and charity work are important interests of this segment. Country music (45%) and folk music (14%) are popular too.

Defining Features

1. Both the age groups 5-14 and 55-64 are over represented in this segment and households with couples and with 5 or more occupants are common
2. Approximately 40% of the labour force work in agriculture, with more than 20% working on their own properties
3. Approximately 17% of homes in these areas are unoccupied

Key Expenditure

Average Australian Household Index = 100

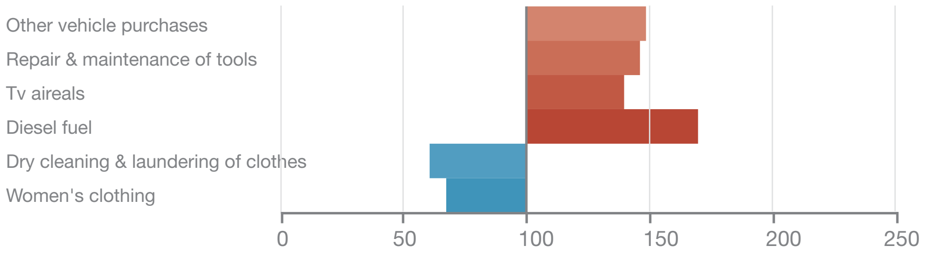

Population Profile

56% of the 166,000 people living in this segment live in Outer Regional areas, and 8% live in Remote areas.

Location Map

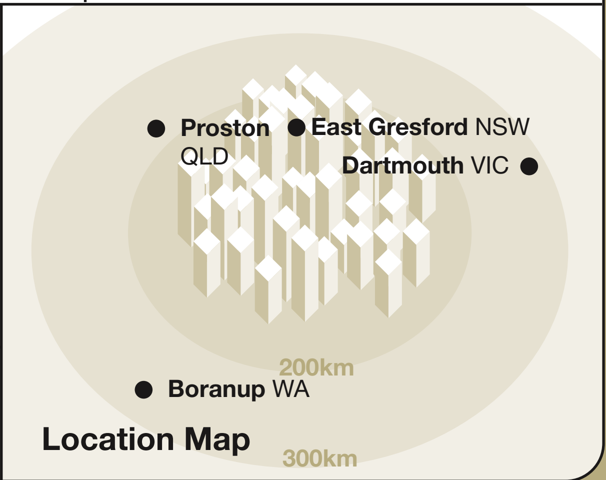

Pathfinder Solutions © Copyright 2003 /// phone: +61-3-9670 3030 /// email: info@pathfindersolutions.com.au

PATHFINDER
SEGMENTS
OF AUSTRALIA

56 Regional & Remote Mining

The hard hats

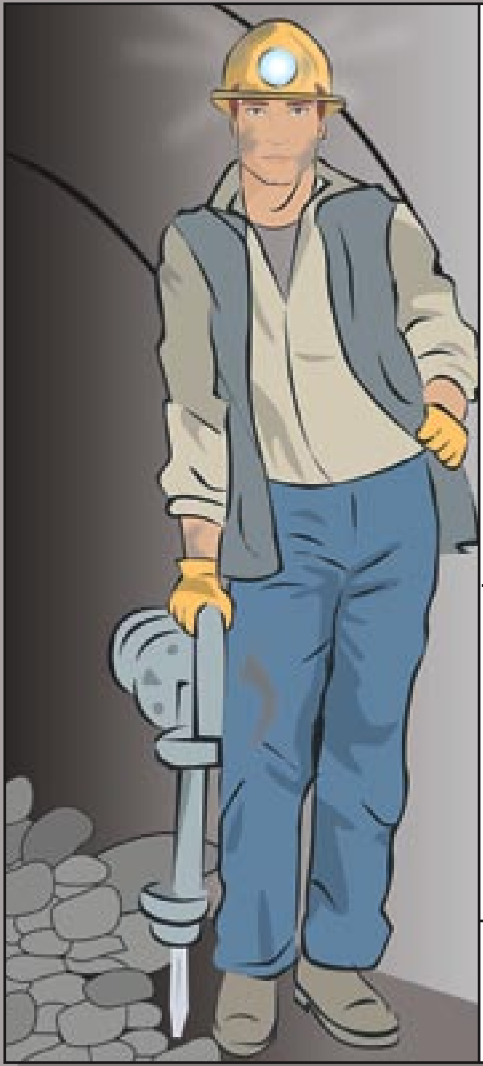

Segment Description

Those who work in mining and extraction areas earn high incomes. In fact in this segment you are twice as likely to find households earning more than $75,000 per year when compared to all segments across Australia.

Often located in outer regional or remote areas, many live in shared accommodation provided by the mining companies. Otherwise, subsidised housing means that monthly housing loans and rent payments are very low. Over 20% of dwellings were unoccupied at the time of the Census reflecting the transient nature of the segment.

Most people here are aged between 25 and 44 years and are employed in transport, production or skilled trades. They take the bus, ride a motorbike, drive a car, ride a bike or walk to work.

This small group can be a bit naughty, with a higher proportion of people who rarely or never pay off their credit card at the end of each month.

There are very high levels of subscription to Pay TV, mail order is popular and many have home PCs and DVD players. Golf and fishing are also popular pastimes.

Defining Features

1. Twice the average level of income above $78,000 p.a.
2. High proportion of indigenous Australians
3. The dominant age range is between 24-44 y.o and 4 times the national average live in non private dwellings due to many people living in shared accommodation provided by the mining companies
4. More than 20% of dwellings were unoccupied at the time of the Census

Key Expenditure

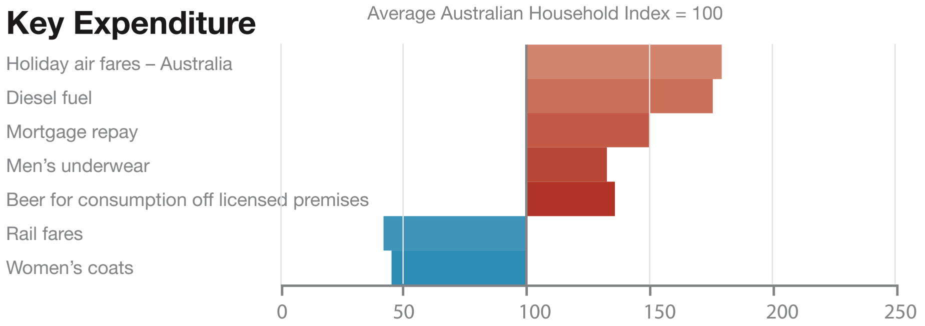

Population Profile

35% of the 61,449 people that live in this segment live in Remote areas of Australia, 28% live in Outer Regional areas, 28% live in Very Remote areas, and 9% live in Inner Regional areas.

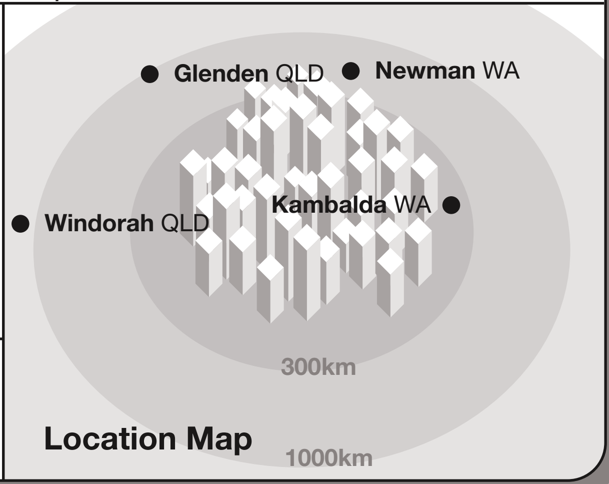

Location Map

Pathfinder Solutions © Copyright 2003 /// phone: +61-3-9670 3030 /// email: info@pathfindersolutions.com.au

PATHFINDER SEGMENTS OF AUSTRALIA

57 Primarily Non-residential

Nobody home!

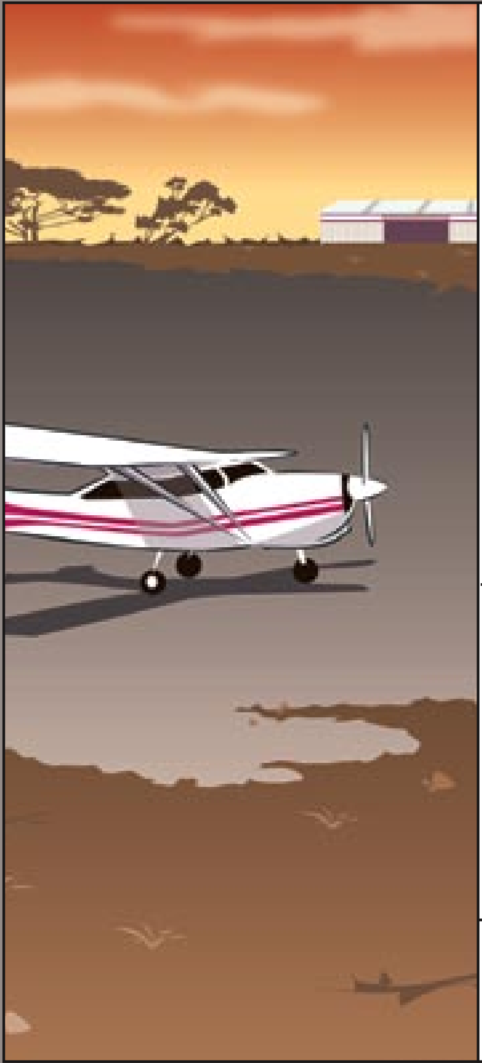

Segment Description

These areas are out in the never reaches, where hardly anybody lives.

They mainly contain commercial, industrial or uninhabited properties.

Around 20% of people who do live in this very small segment live in non-private dwellings and around 18% are overseas visitors.

Highlighting just how much of this great brown land is uninhabited, the total number of people in these areas is less than 1200, while covering more than 800,000 square kilometres of Australia!

Defining Features

1. There are only 78 people over the age of 64 in this small segment
2. One parent families with children under 15 are over represented in this segment
3. 20% of people live in non private dwellings and around 18% of people are overseas visitors
4. The total number of people in these areas is less than 1200, while covering 811,825 square kilometres of Australia

Key Expenditure

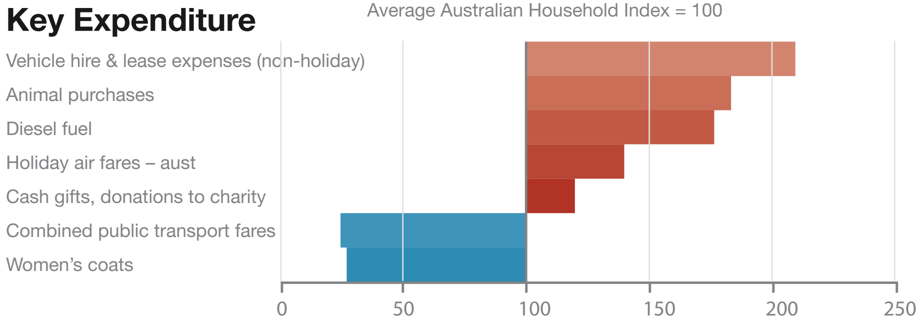

Population Profile

38% of the 1,111 people that live in this segment live in Very Remote areas of Australia, 26% live in Remote areas, 22% live in Outer Regional areas, and 8% live in Major Cities.

Location Map

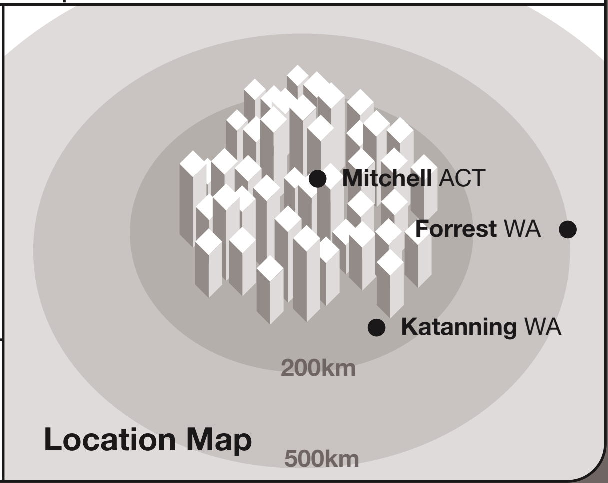

Pathfinder Solutions © Copyright 2003 /// phone: +61-3-9670 3030 /// email: info@pathfindersolutions.com.au

PATHFINDER SEGMENTS OF AUSTRALIA

58 Defence Enclaves

Home base

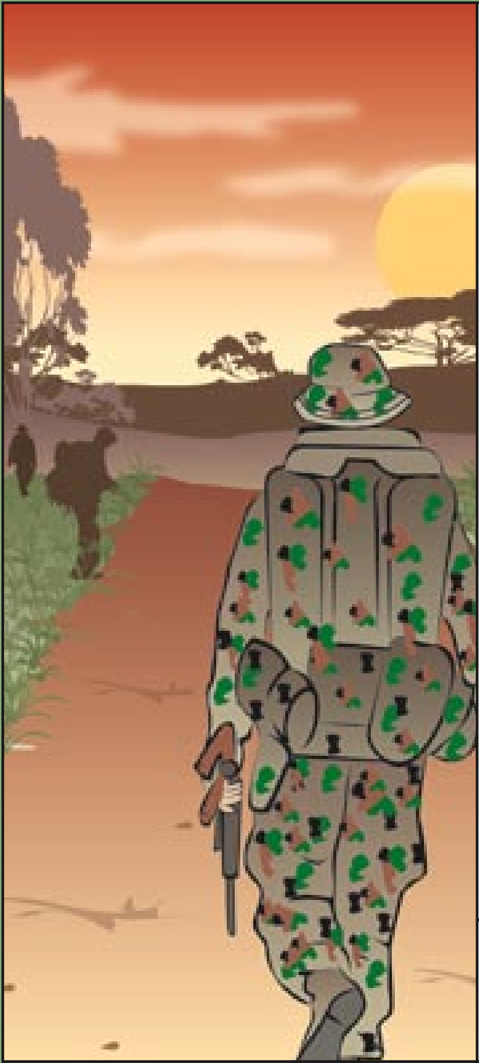

Segment Description

Conjuring up images of men and women in uniforms lined up saluting the flag, this segment is scattered across Australia.

It includes purpose built defence bases, such as Richmond, Tindal, and Jervis Bay. Many of the bases are located in remote parts of Australia, and are in areas that also contain significant proportions of indigenous Australians.

Almost all males in this segment are employed in defence jobs though there are also numbers of tradesmen. Household incomes are strongest in the $35,000 to $50,000 per year band. Personal loans are very popular among this segment.

They're a fit group, with almost a quarter involved in active sport, including volleyball, working out at a gym, skiing and scuba diving.

Watch out if you're not into pop/rock music as 77% of the population lists it as a favourite.

Defining Features

1. Majority of males and females in this segment are employed in defence jobs
2. 74% of people are aged under 34 years of age
3. 90% of people in this segment were born in Australia
4. Significant proportions of indigenous Australians

Key Expenditure

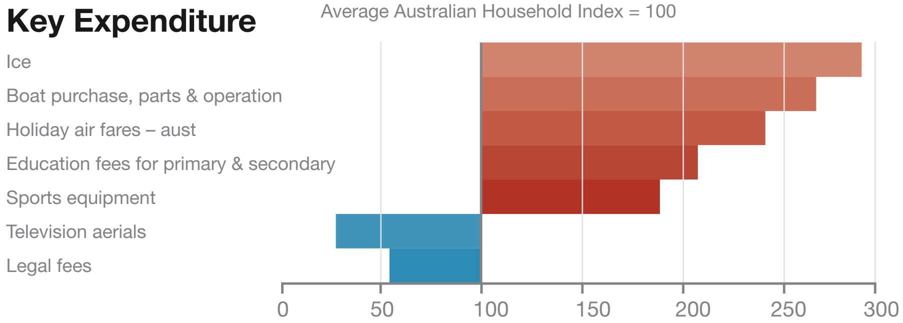

Population Profile

47% of the 61,014 people that live in this segment live in Very Remote areas of Australia, 16% live in Outer Regional areas, 15% in Inner Regional areas, 12% in Major Cities and 7% in Remote areas.

Location Map

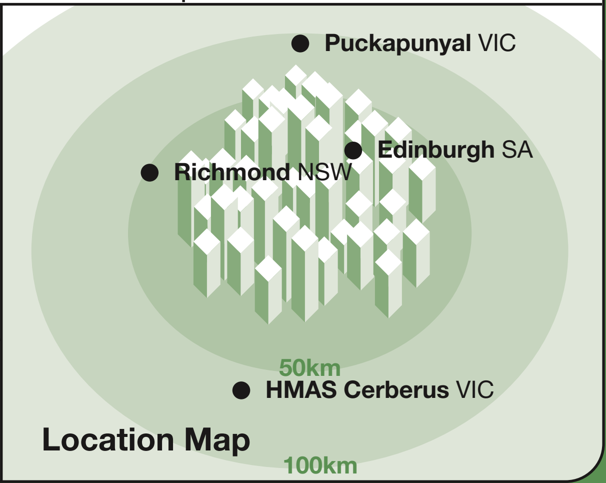

Pathfinder Solutions © Copyright 2003 /// phone: +61-3-9670 3030 /// email: info@pathfindersolutions.com.au

PATHFINDER SEGMENTS OF AUSTRALIA

59 Remote Koori Lands & Stations

The heartland

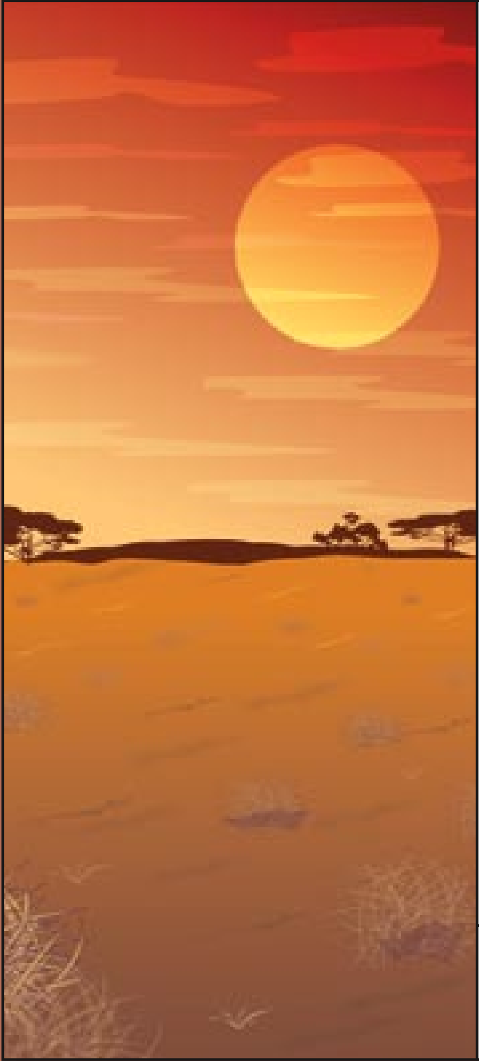

Segment Description

These extremely isolated areas are far away from towns and cities and over two thirds of the population are indigenous Australians.

Tourists make up around 10% of the population. People under 14 and aged between 25 and 29 are most common. A reflection of low life expectance for indigenous Australians is the low percentage aged over 70.

Households are large, usually having six or more occupants. Almost half (45%) speak a language other than English at home, and low levels of education are common with around a quarter having left school at Year 8 or earlier. Around 7% have never gone to school at all.

Household income is over represented in the $35,000 to-$40,000 per year band and very high incomes are vastly under represented. Labouring jobs are common.

Around a third of the inhabitants of this segment walk to work each day.

Defining Features

1. 2/3 of the people living in these areas are indigenous Australians
2. On Census night 10% of people in these areas were visitors
3. Children under 14 are over represented and older age groups, particularly over 70 are under represented, reflecting a lower life expectancy for indigenous Australians
4. Low levels of education are common with 3/4 of all people leaving school before Year 8 and 7% never having gone to school at all

Key Expenditure

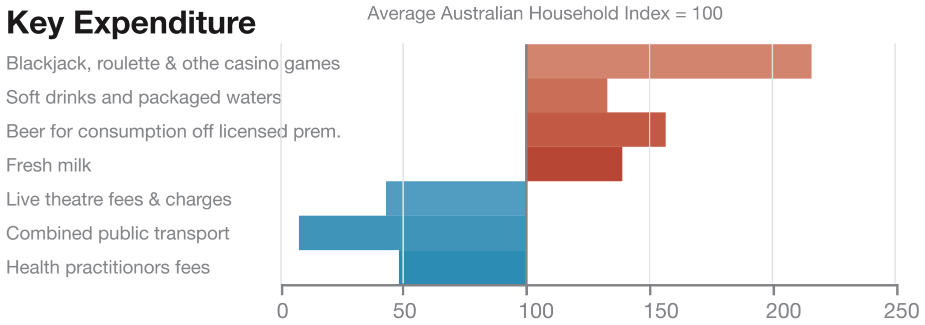

Population Profile

100% of the 46,176 people living in these areas live in Very Remote areas.

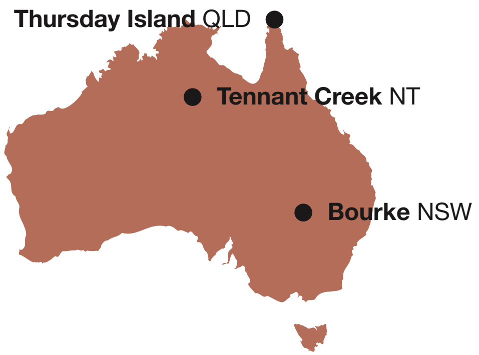

Location Map

Pathfinder Solutions © Copyright 2003 /// phone: +61-3-9670 3030 /// email: info@pathfindersolutions.com.au

PATHFINDER SEGMENTS OF AUSTRALIA

60 Offshore, Shipping & Migratory

Swept away

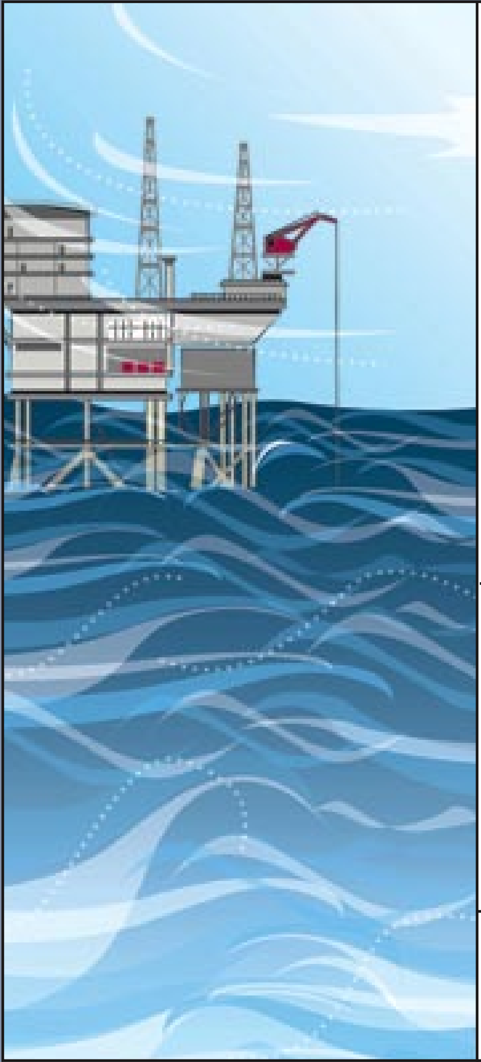

Segment Description

This very small, remote group accounts for those who live away from Australia or those who work in shipping or other maritime activities such as on oil rigs.

Those who work in such professions are likely to earn over $50,000 per year with people earning in excess of $75,000 per year also over represented in this segment.

A great proportion of those included in this segment are overseas visitors.

Defining Features

1. Only accounts for 7994 people (less than 1/20th of 1% of the population!)
2. Dominated by shipping, maritime and oil rig occupations
3. High incomes are over represented
4. Many people are visitors from overseas

Key Variables

Not applicable

Population Profile

100% of the 7994 people living in this segment live in areas the ABS classifies as offshore, shipping & migratory

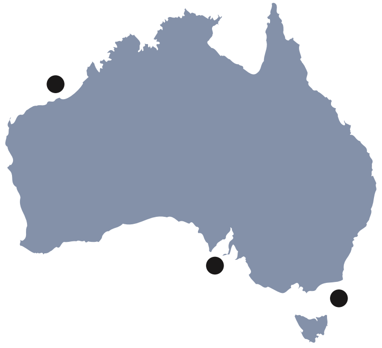

Location Map
